# Supplementary figures and images for: mTOR inhibition in Q175 Huntington’s disease model mice facilitates neuronal autophagy and mutant huntingtin clearance (part 1 of 2)
Source: eLife. 2025 May 20;14:RP104979. doi: 10.7554/eLife.104979 (PMC12092004; doi:10.7554/eLife.104979)

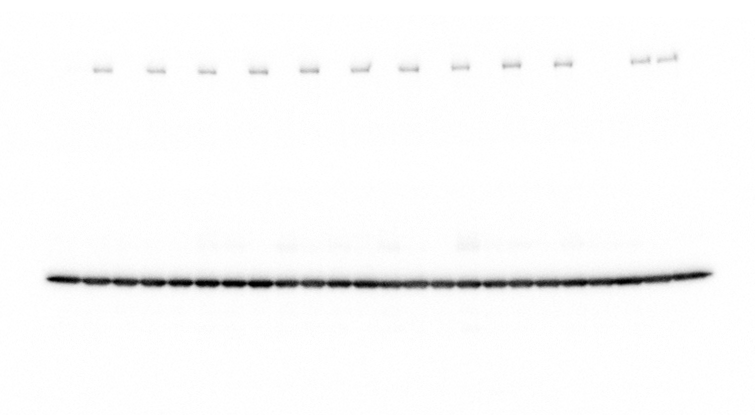

Supplement: Figure 1—source data 1. [file elife-104979-fig1-data1.zip › Figure 1C-Source Data 1/Fig 1C1, Left_Anti-GAPDH (bottom)_Short expos..tif]

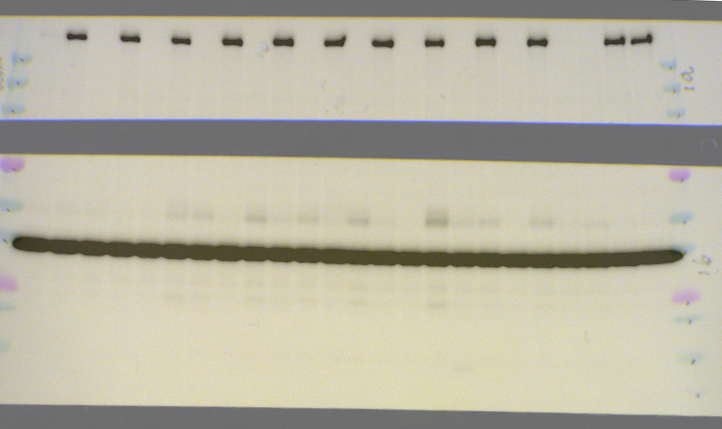

Supplement: Figure 1—source data 1. [file elife-104979-fig1-data1.zip › Figure 1C-Source Data 1/Fig 1C1, Left_Anti-Htt (1574, top) & anti-GAPDH (bottom)_Overlayed.tif]

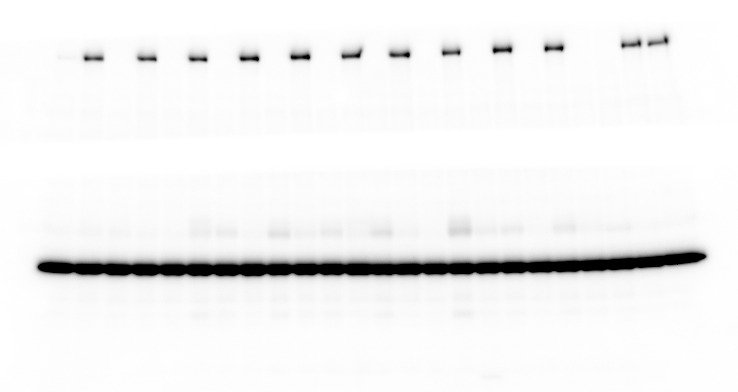

Supplement: Figure 1—source data 1. [file elife-104979-fig1-data1.zip › Figure 1C-Source Data 1/Fig 1C1, Left_Anti-Htt (1574, top)_Short expos..tif]

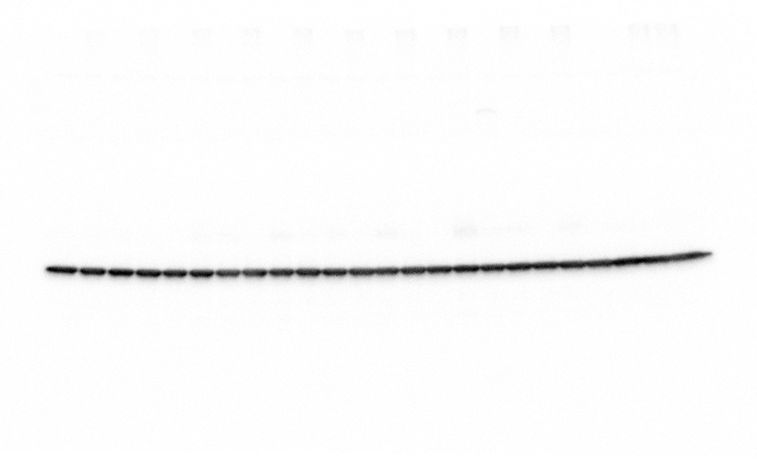

Supplement: Figure 1—source data 1. [file elife-104979-fig1-data1.zip › Figure 1C-Source Data 1/Fig 1C1, Right_Anti-GAPDH (bottom)_Short expos..tif]

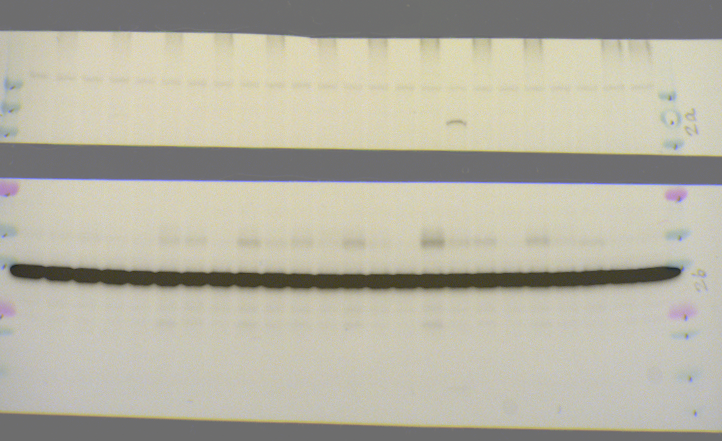

Supplement: Figure 1—source data 1. [file elife-104979-fig1-data1.zip › Figure 1C-Source Data 1/Fig 1C1, Right_Anti-Htt (PHP2, top) & anti-GAPDH (bott)_Overlayed.tif]

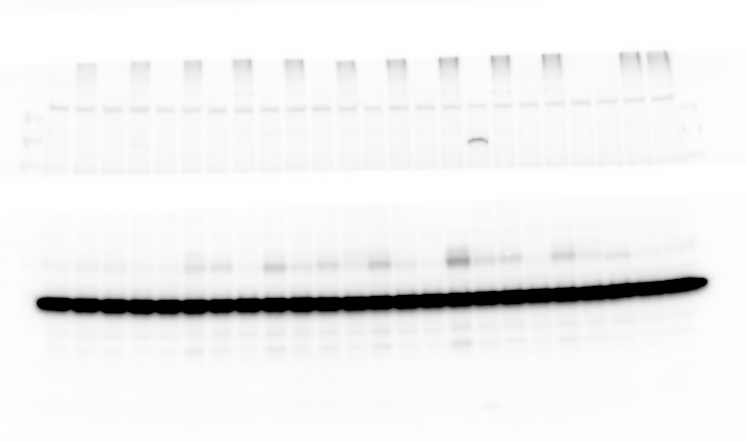

Supplement: Figure 1—source data 1. [file elife-104979-fig1-data1.zip › Figure 1C-Source Data 1/Fig 1C1, Right_Anti-Htt (PHP2, top)_Short expos..tif]

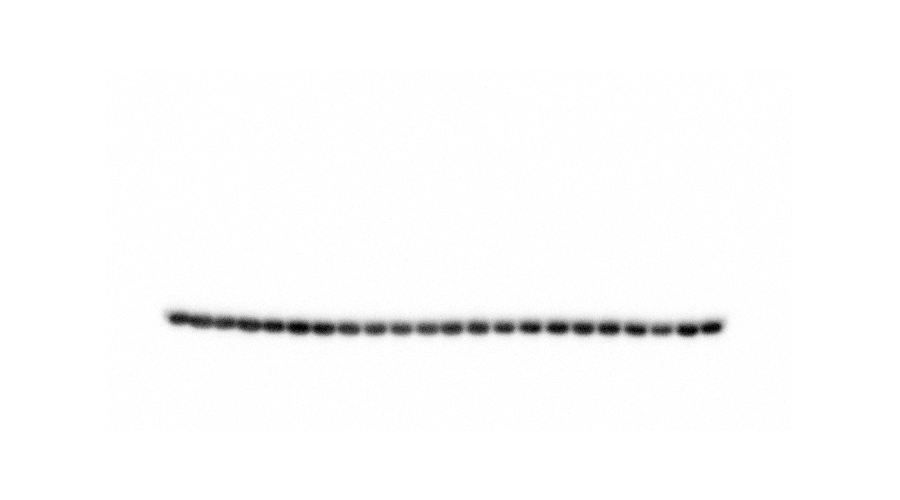

Supplement: Figure 1—source data 1. [file elife-104979-fig1-data1.zip › Figure 1C-Source Data 1/Fig 1C2, Left_Anti-GAPDH.tif]

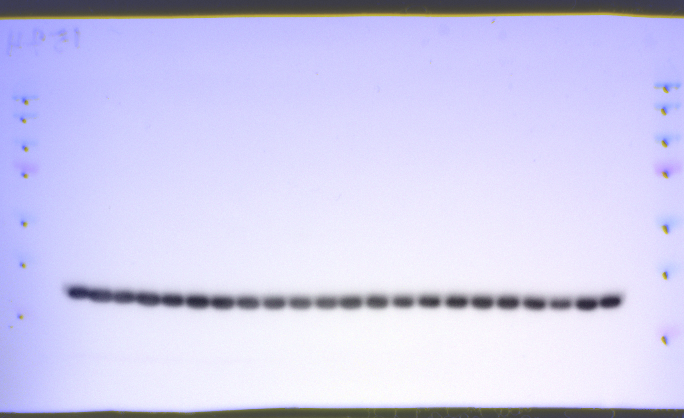

Supplement: Figure 1—source data 1. [file elife-104979-fig1-data1.zip › Figure 1C-Source Data 1/Fig 1C2, Left_Anti-GAPDH_Overlayed.tif]

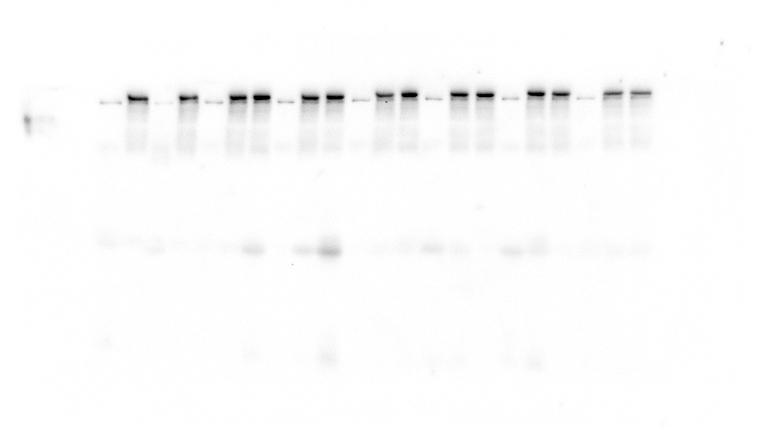

Supplement: Figure 1—source data 1. [file elife-104979-fig1-data1.zip › Figure 1C-Source Data 1/Fig 1C2, Left_Anti-Htt (1574).tif]

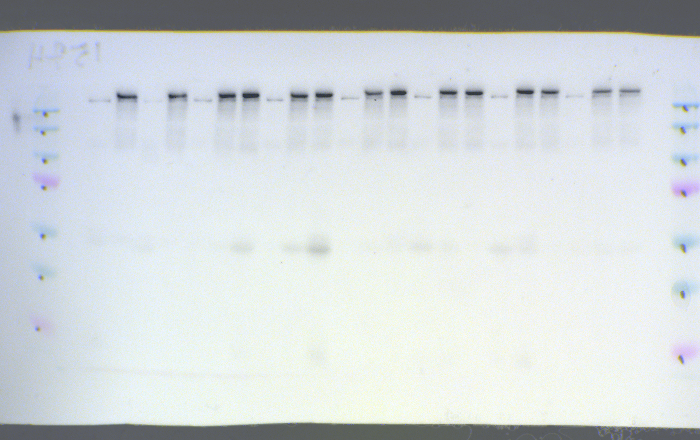

Supplement: Figure 1—source data 1. [file elife-104979-fig1-data1.zip › Figure 1C-Source Data 1/Fig 1C2, Left_Anti-Htt (1574)_Overlayed.tif]

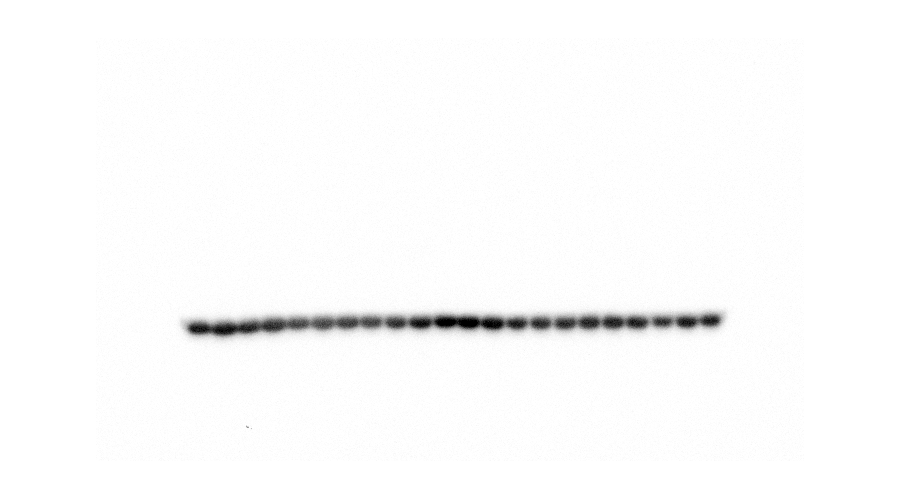

Supplement: Figure 1—source data 1. [file elife-104979-fig1-data1.zip › Figure 1C-Source Data 1/Fig 1C2, Right_Anti-GAPDH.tif]

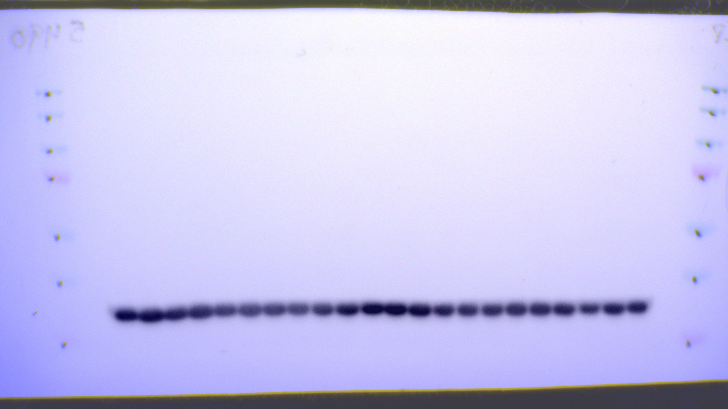

Supplement: Figure 1—source data 1. [file elife-104979-fig1-data1.zip › Figure 1C-Source Data 1/Fig 1C2, Right_Anti-GAPDH_Overlayed.tif]

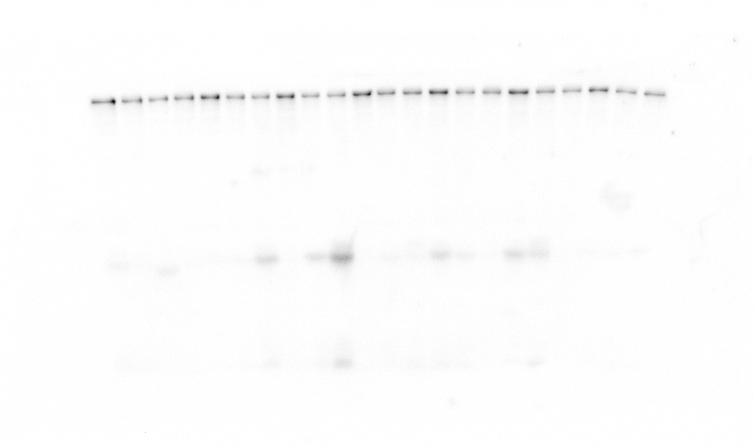

Supplement: Figure 1—source data 1. [file elife-104979-fig1-data1.zip › Figure 1C-Source Data 1/Fig 1C2, Right_Anti-Htt (5490).tif]

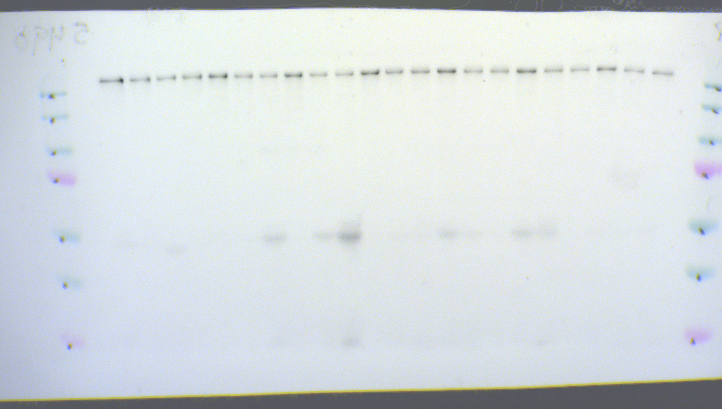

Supplement: Figure 1—source data 1. [file elife-104979-fig1-data1.zip › Figure 1C-Source Data 1/Fig 1C2, Right_Anti-Htt (5490)_Overlayed.tif]

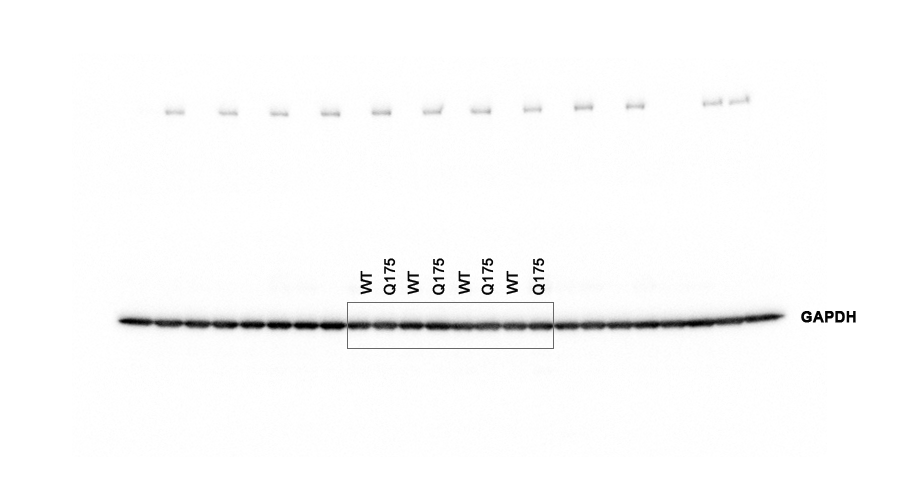

Supplement: Figure 1—source data 2. [file elife-104979-fig1-data2.zip › Figure 1C-Source Data 2/Fig 1C1, Left_Anti-GAPDH (bottom)_Short expos. & Labeled.tif]

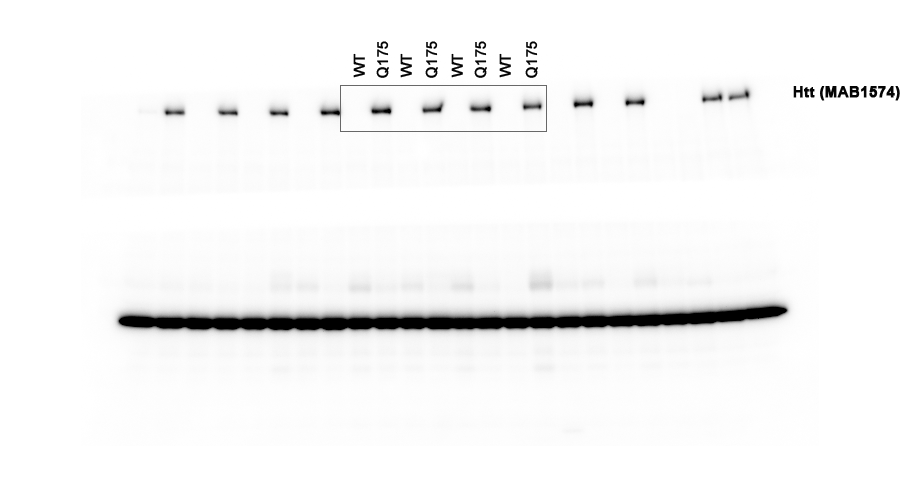

Supplement: Figure 1—source data 2. [file elife-104979-fig1-data2.zip › Figure 1C-Source Data 2/Fig 1C1, Left_Anti-Htt (1574, top)_Short expos. & Labeled.tif]

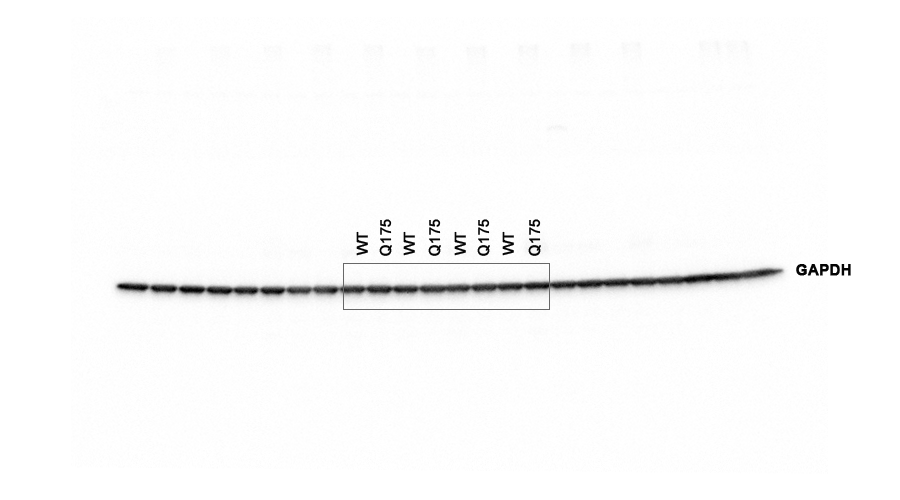

Supplement: Figure 1—source data 2. [file elife-104979-fig1-data2.zip › Figure 1C-Source Data 2/Fig 1C1, Right_Anti-GAPDH (bottom)_Short expos. & Labeled.tif]

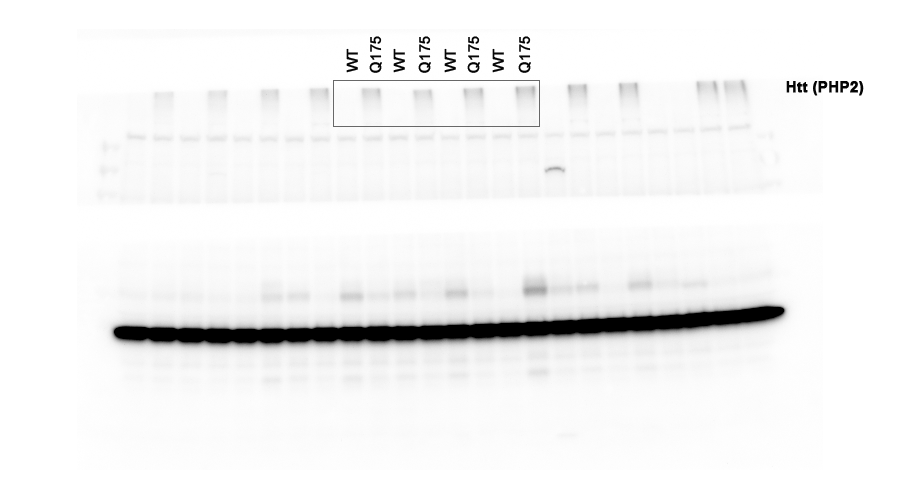

Supplement: Figure 1—source data 2. [file elife-104979-fig1-data2.zip › Figure 1C-Source Data 2/Fig 1C1, Right_Anti-Htt (PHP2, top)_Short expos. & Labeled.tif]

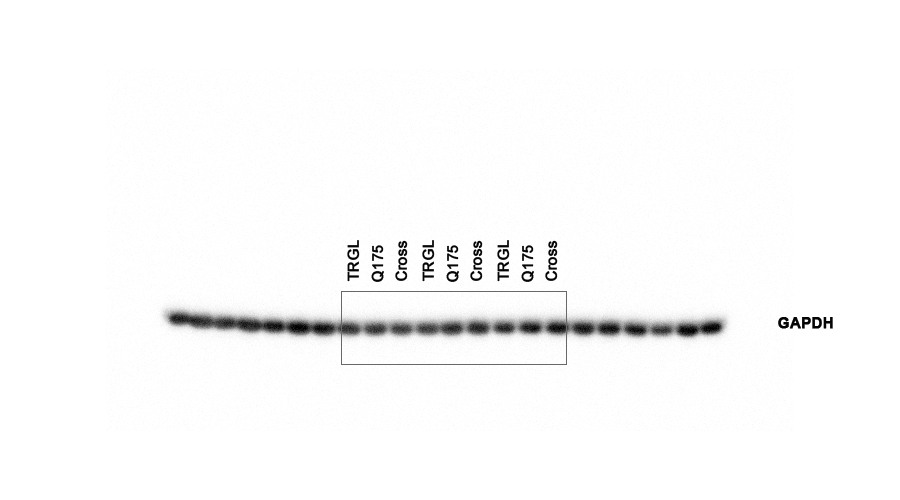

Supplement: Figure 1—source data 2. [file elife-104979-fig1-data2.zip › Figure 1C-Source Data 2/Fig 1C2, Left_Anti-GAPDH_Labeled.tif]

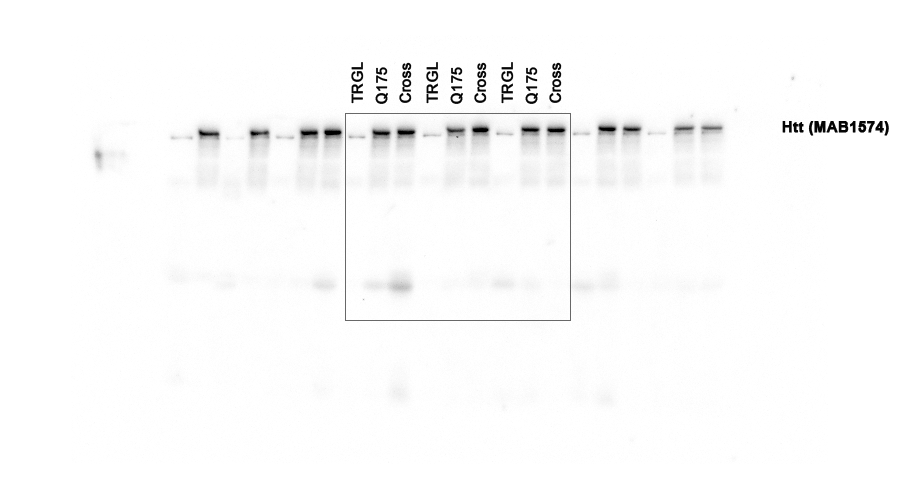

Supplement: Figure 1—source data 2. [file elife-104979-fig1-data2.zip › Figure 1C-Source Data 2/Fig 1C2, Left_Anti-Htt (1574)_Labeled.tif]

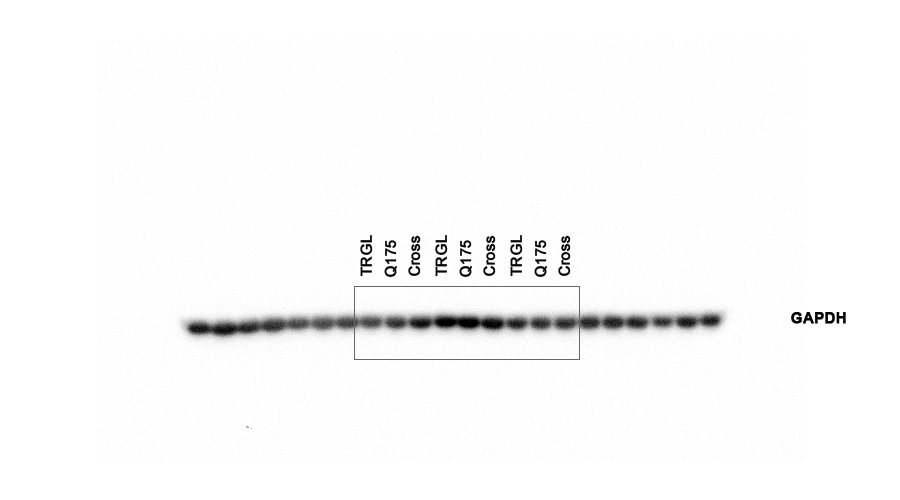

Supplement: Figure 1—source data 2. [file elife-104979-fig1-data2.zip › Figure 1C-Source Data 2/Fig 1C2, Right_Anti-GAPDH_Labeled.tif]

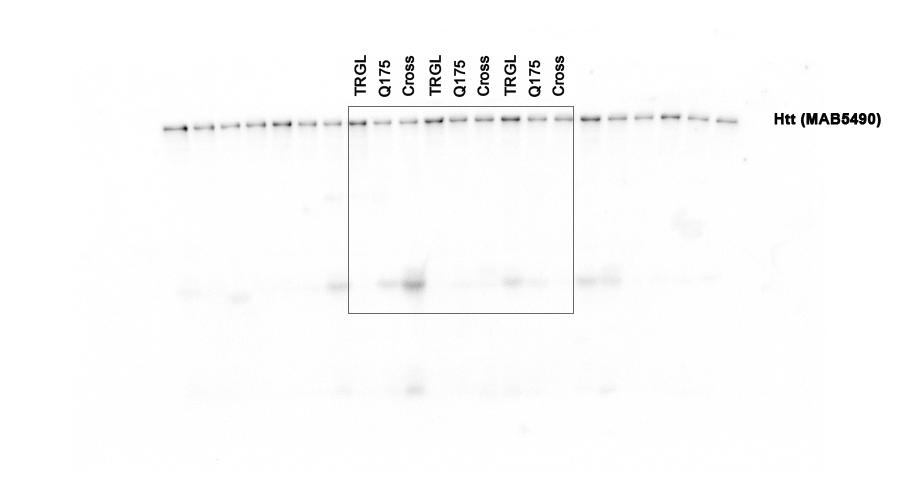

Supplement: Figure 1—source data 2. [file elife-104979-fig1-data2.zip › Figure 1C-Source Data 2/Fig 1C2, Right_Anti-Htt (5490)_Labeled.tif]

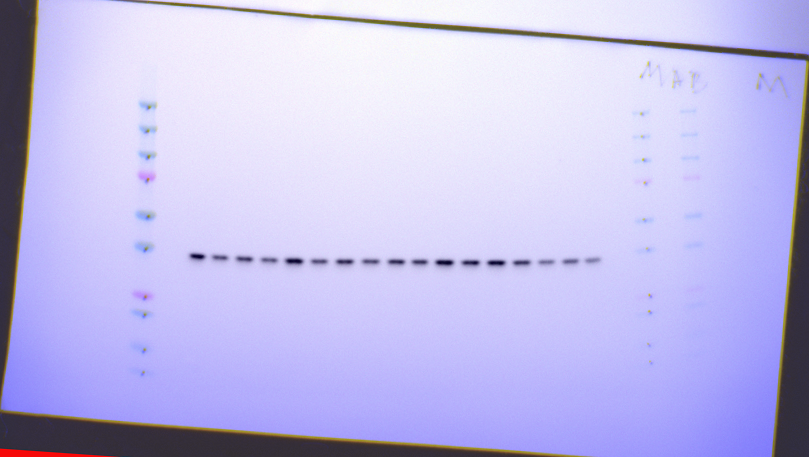

Supplement: Figure 3—figure supplement 1—source data 1. [file elife-104979-fig3-figsupp1-data1.zip › Figure 3-Figure supplement 1-Source Data 1/Anti-GAPDH (for mHTT)_Overlayed.tif]

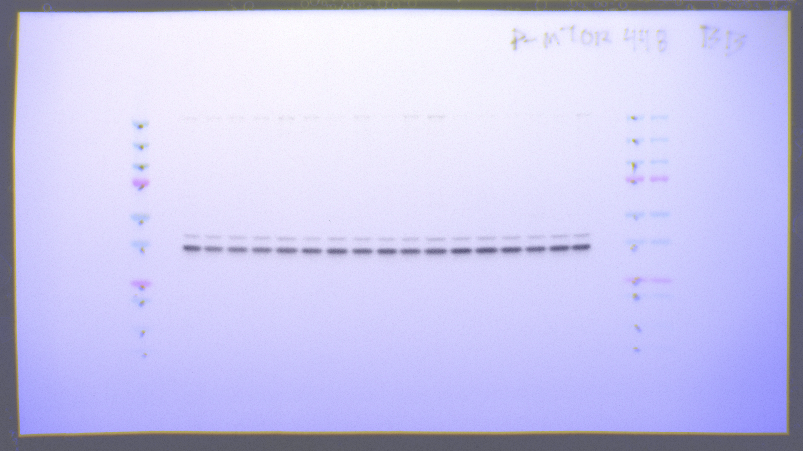

Supplement: Figure 3—figure supplement 1—source data 1. [file elife-104979-fig3-figsupp1-data1.zip › Figure 3-Figure supplement 1-Source Data 1/Anti-GAPDH (for p-MTOR S2448)_Overlayed.tif]

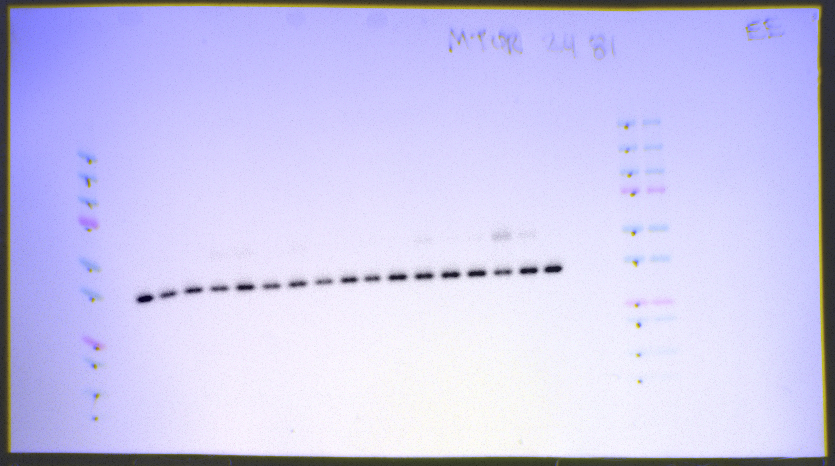

Supplement: Figure 3—figure supplement 1—source data 1. [file elife-104979-fig3-figsupp1-data1.zip › Figure 3-Figure supplement 1-Source Data 1/Anti-GAPDH (for p-MTOR S2481)_Overlayed.tif]

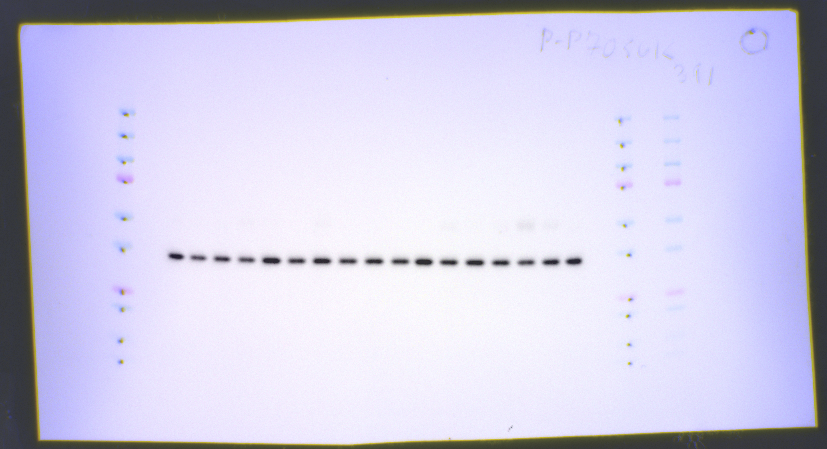

Supplement: Figure 3—figure supplement 1—source data 1. [file elife-104979-fig3-figsupp1-data1.zip › Figure 3-Figure supplement 1-Source Data 1/Anti-GAPDH (for p-p70S6K S371)_Overlayed.tif]

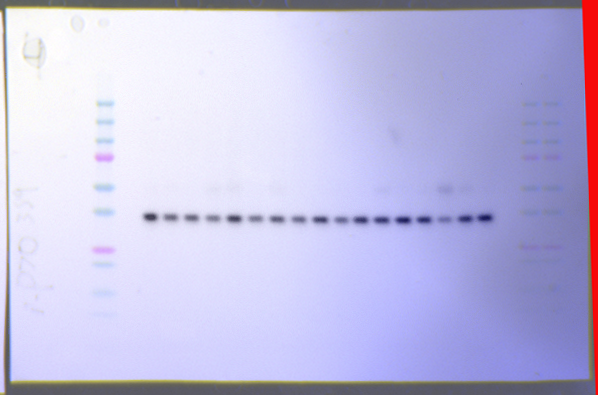

Supplement: Figure 3—figure supplement 1—source data 1. [file elife-104979-fig3-figsupp1-data1.zip › Figure 3-Figure supplement 1-Source Data 1/Anti-GAPDH (for p-p70S6K T389)_Overlayed.tif]

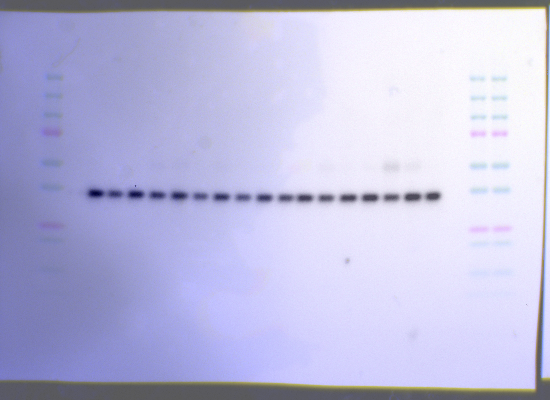

Supplement: Figure 3—figure supplement 1—source data 1. [file elife-104979-fig3-figsupp1-data1.zip › Figure 3-Figure supplement 1-Source Data 1/Anti-GAPDH (for p-ULK1 S317)_Overlayed.tif]

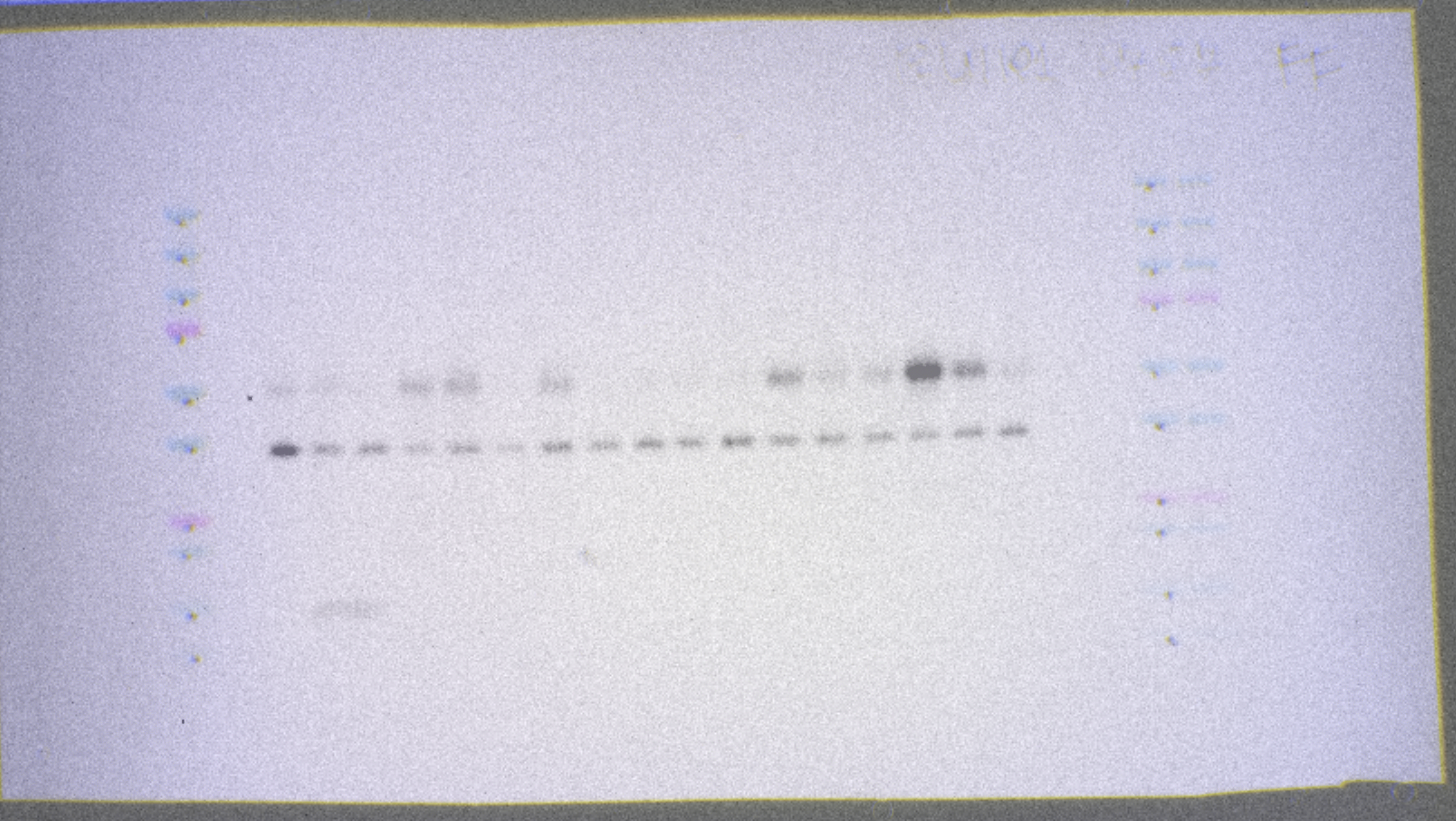

Supplement: Figure 3—figure supplement 1—source data 1. [file elife-104979-fig3-figsupp1-data1.zip › Figure 3-Figure supplement 1-Source Data 1/Anti-GAPDH (for p-ULK1 S757)_Overlayed.tif]

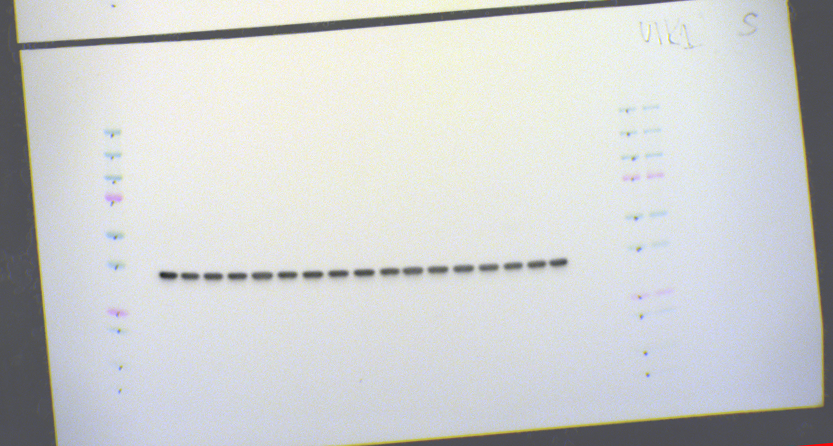

Supplement: Figure 3—figure supplement 1—source data 1. [file elife-104979-fig3-figsupp1-data1.zip › Figure 3-Figure supplement 1-Source Data 1/Anti-GAPDH (for ULK1)_Overlayed.tif]

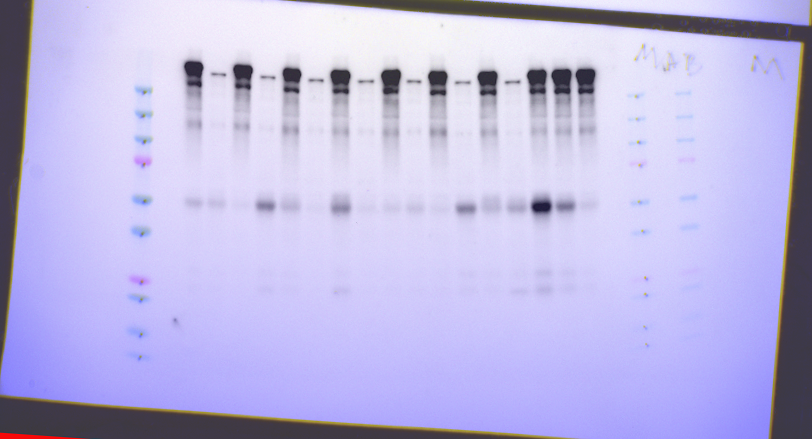

Supplement: Figure 3—figure supplement 1—source data 1. [file elife-104979-fig3-figsupp1-data1.zip › Figure 3-Figure supplement 1-Source Data 1/Anti-mHTT_Overlayed.tif]

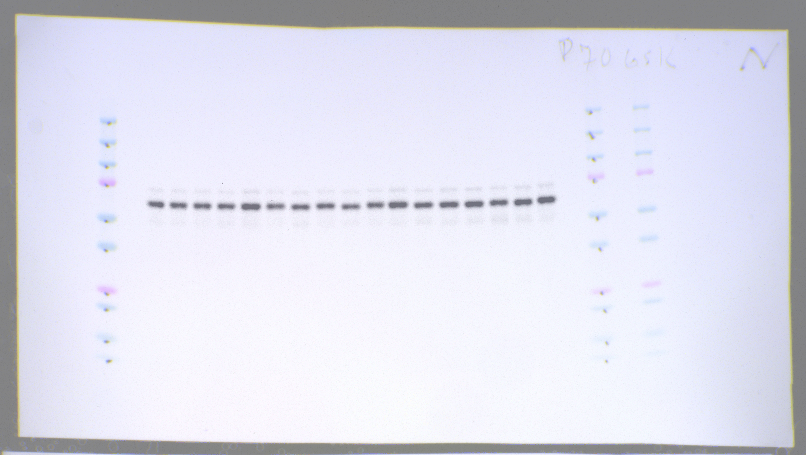

Supplement: Figure 3—figure supplement 1—source data 1. [file elife-104979-fig3-figsupp1-data1.zip › Figure 3-Figure supplement 1-Source Data 1/Anti-P70S6K_Overlayed.tif]

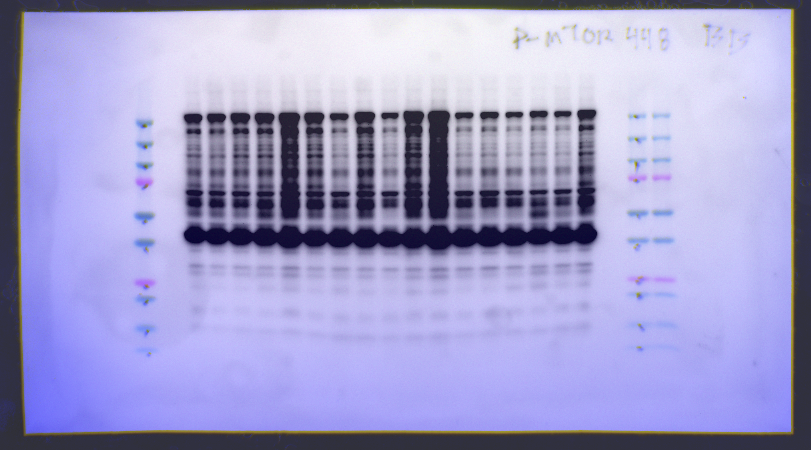

Supplement: Figure 3—figure supplement 1—source data 1. [file elife-104979-fig3-figsupp1-data1.zip › Figure 3-Figure supplement 1-Source Data 1/Anti-p-MTOR (S2448)_Overlayed.tif]

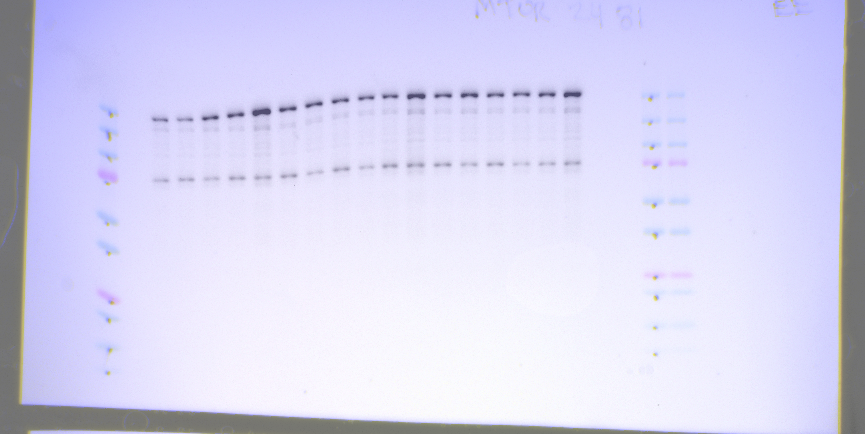

Supplement: Figure 3—figure supplement 1—source data 1. [file elife-104979-fig3-figsupp1-data1.zip › Figure 3-Figure supplement 1-Source Data 1/Anti-p-MTOR (S2481)_Overlayed.tif]

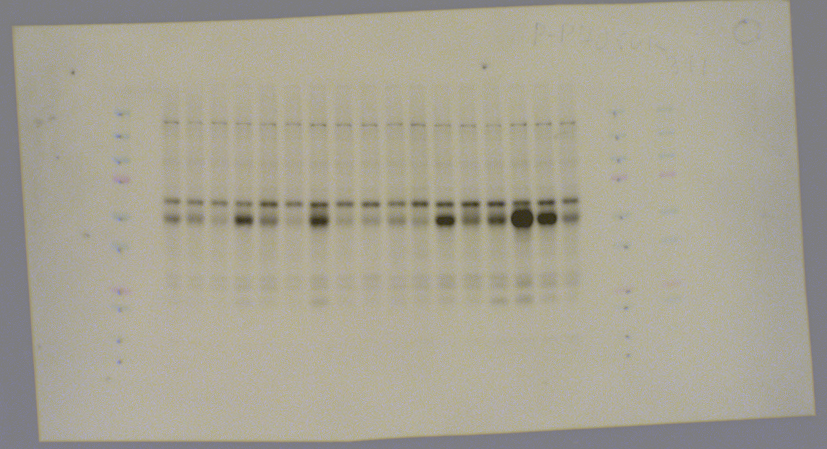

Supplement: Figure 3—figure supplement 1—source data 1. [file elife-104979-fig3-figsupp1-data1.zip › Figure 3-Figure supplement 1-Source Data 1/Anti-p-p70S6K (S371)_Overlayed.tif]

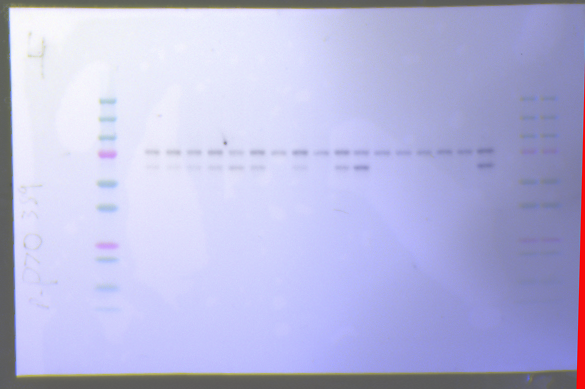

Supplement: Figure 3—figure supplement 1—source data 1. [file elife-104979-fig3-figsupp1-data1.zip › Figure 3-Figure supplement 1-Source Data 1/Anti-p-p70S6K (T389)_Overlayed.tif]

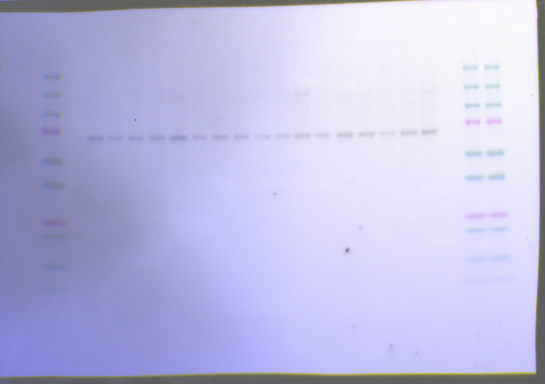

Supplement: Figure 3—figure supplement 1—source data 1. [file elife-104979-fig3-figsupp1-data1.zip › Figure 3-Figure supplement 1-Source Data 1/Anti-p-ULK1 (S317)_Overlayed.tif]

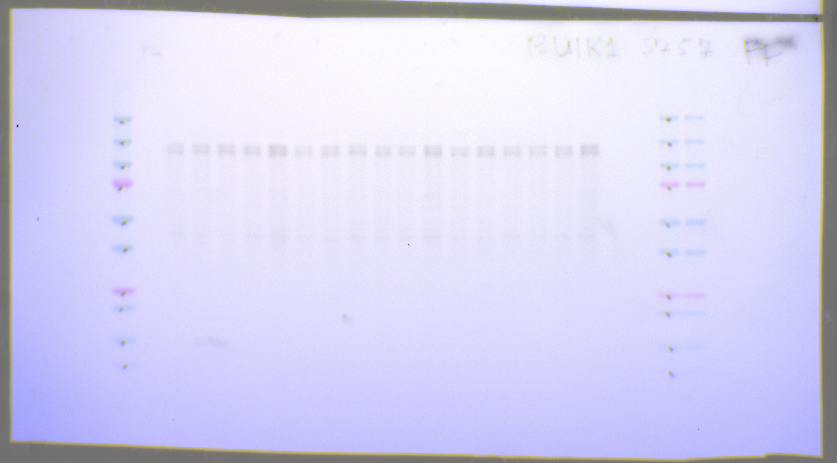

Supplement: Figure 3—figure supplement 1—source data 1. [file elife-104979-fig3-figsupp1-data1.zip › Figure 3-Figure supplement 1-Source Data 1/Anti-p-ULK1 (S757)_Overlayed.tif]

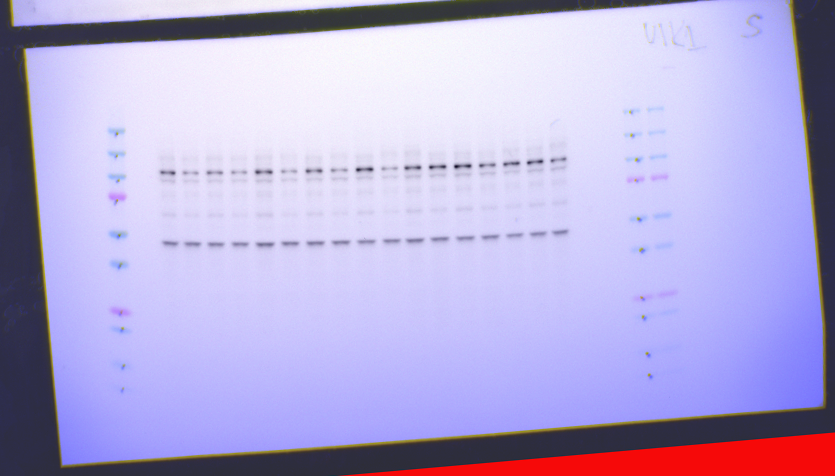

Supplement: Figure 3—figure supplement 1—source data 1. [file elife-104979-fig3-figsupp1-data1.zip › Figure 3-Figure supplement 1-Source Data 1/Anti-ULK1_Overlayed.tif]

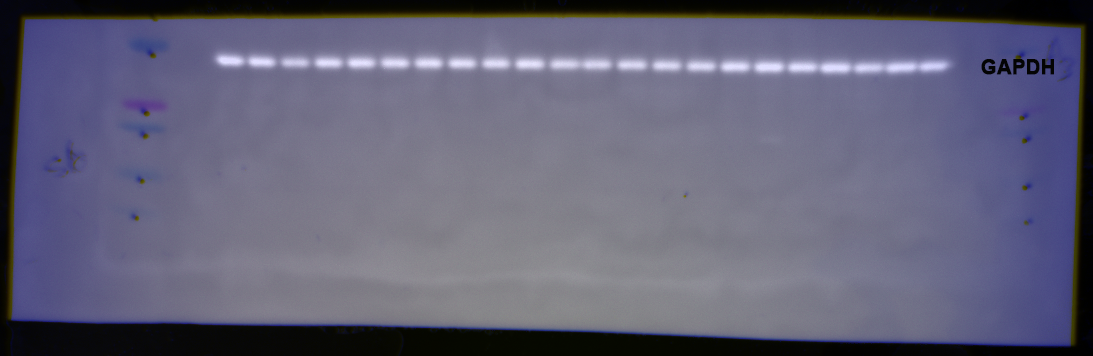

Supplement: Figure 3—figure supplement 1—source data 1. [file elife-104979-fig3-figsupp1-data1.zip › Figure 3-Figure supplement 1-Source Data 1/MTOR only/Anti-GAPDH (for MTOR used for quantitation-only)_Overlayed.tif]

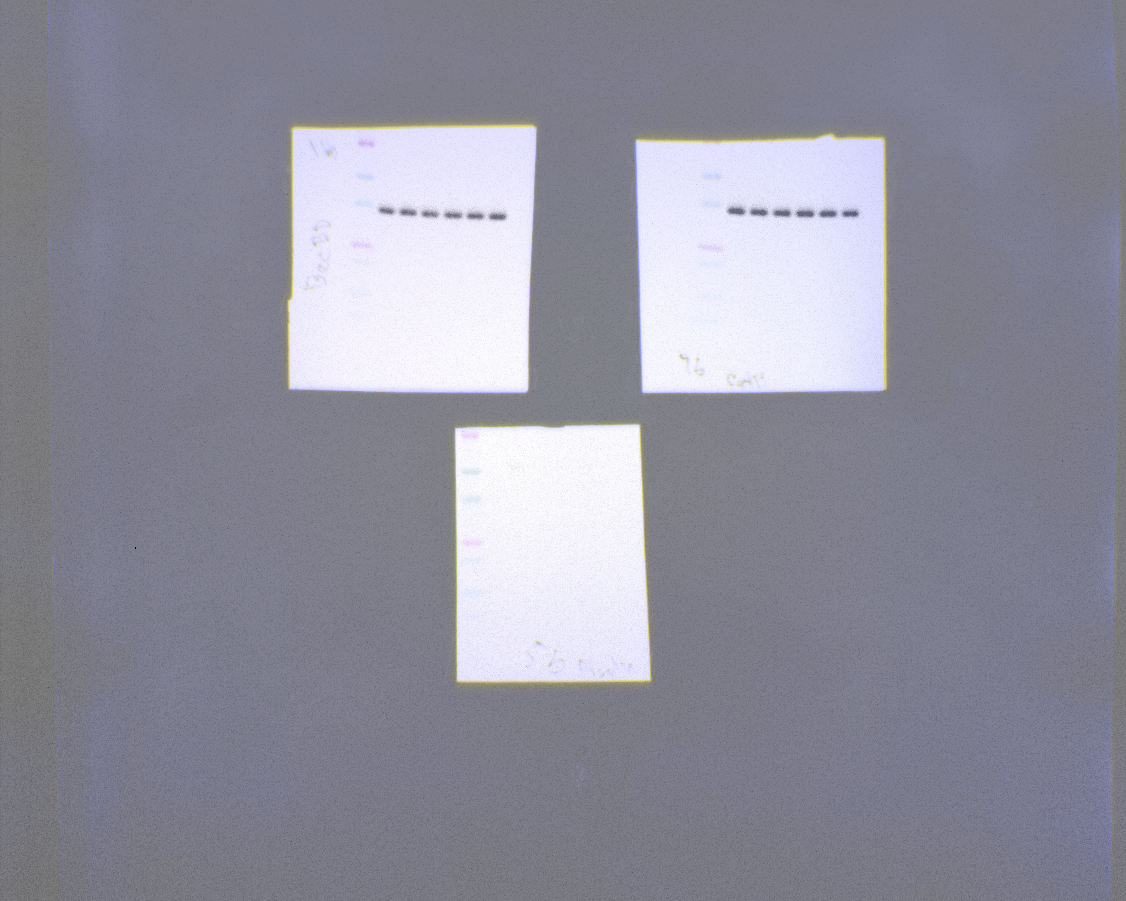

Supplement: Figure 3—figure supplement 1—source data 1. [file elife-104979-fig3-figsupp1-data1.zip › Figure 3-Figure supplement 1-Source Data 1/MTOR only/Anti-GAPDH (upper left, for MTOR & Beclin1 used for display-only)_Overlayed.tif]

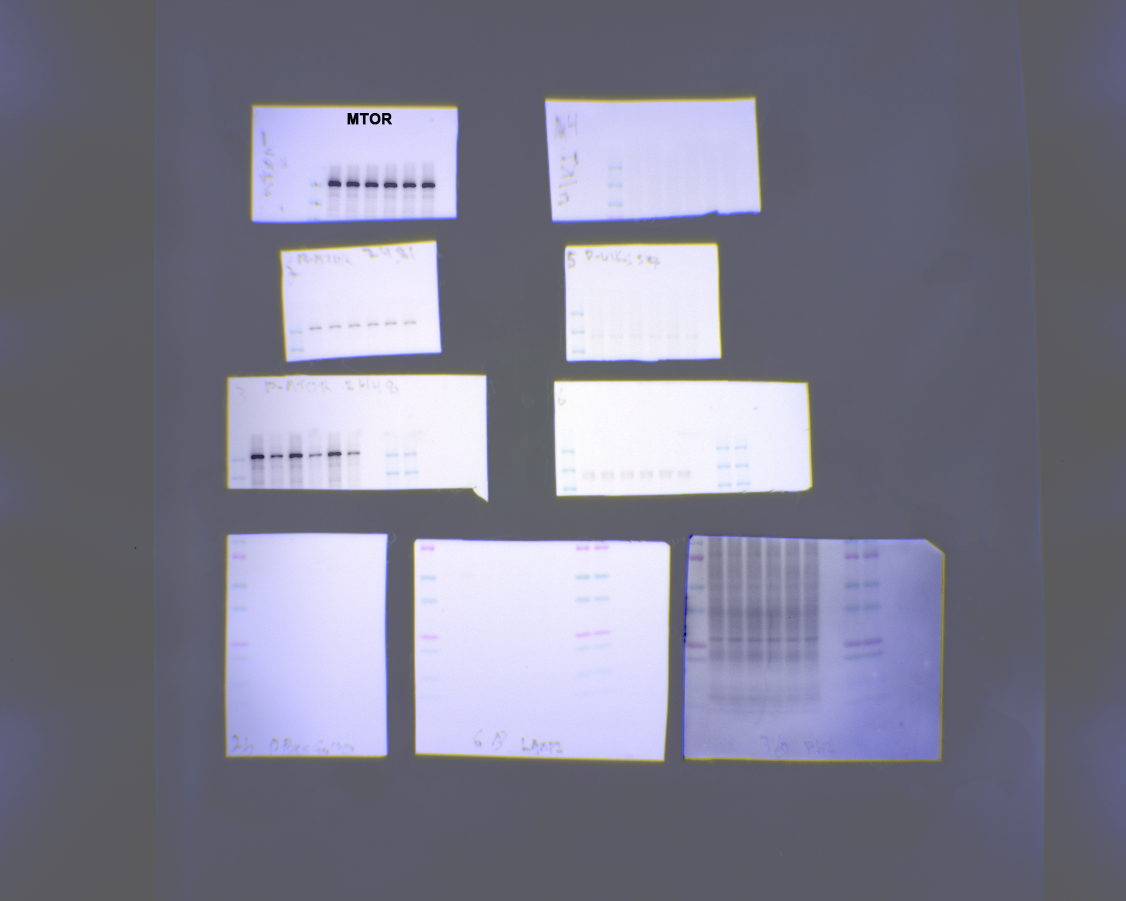

Supplement: Figure 3—figure supplement 1—source data 1. [file elife-104979-fig3-figsupp1-data1.zip › Figure 3-Figure supplement 1-Source Data 1/MTOR only/Anti-MTOR (upper-left, used for display-only)_Overlayed.tif]

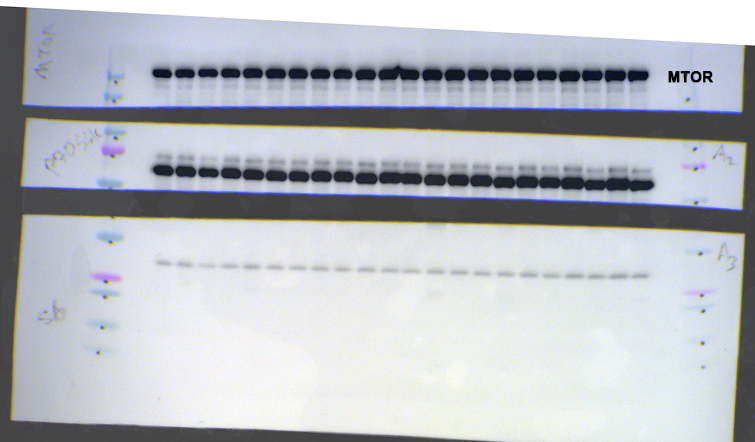

Supplement: Figure 3—figure supplement 1—source data 1. [file elife-104979-fig3-figsupp1-data1.zip › Figure 3-Figure supplement 1-Source Data 1/MTOR only/Anti-MTOR (used for quantitation-only)_Overlayed.tif]

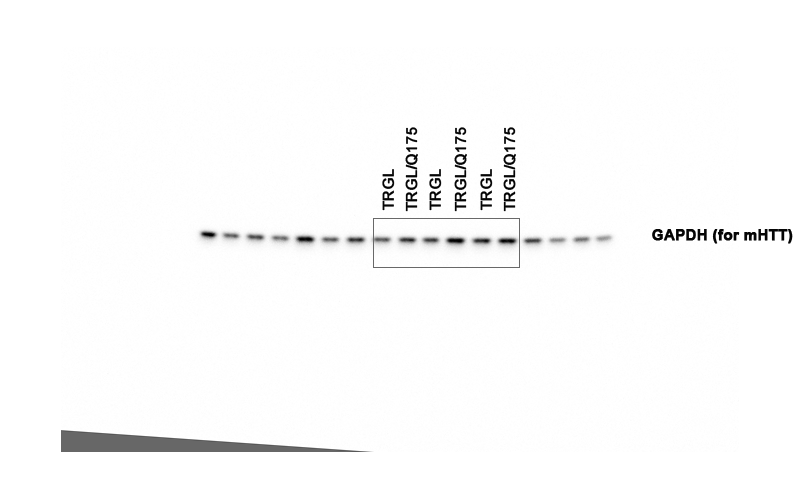

Supplement: Figure 3—figure supplement 1—source data 2. [file elife-104979-fig3-figsupp1-data2.zip › Figure 3-Figure supplement 1-Source Data 2/Anti-GAPDH (for mHTT)_Short expos & Labeled.tif]

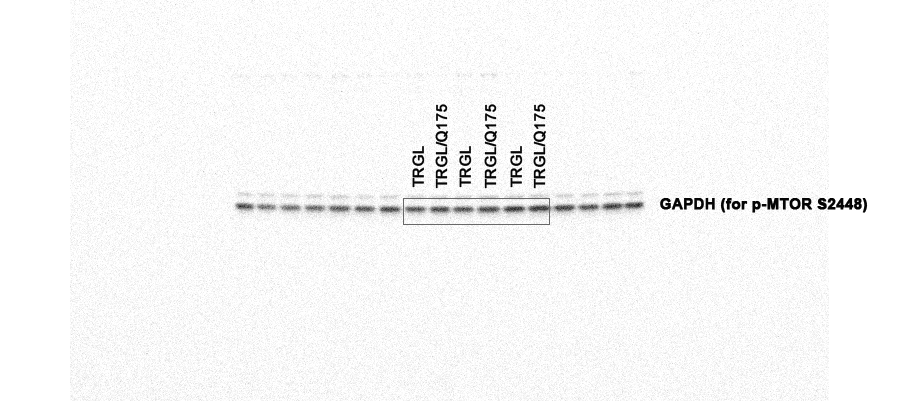

Supplement: Figure 3—figure supplement 1—source data 2. [file elife-104979-fig3-figsupp1-data2.zip › Figure 3-Figure supplement 1-Source Data 2/Anti-GAPDH (for p-MTOR S2448)_Short expos & Labeled.tif]

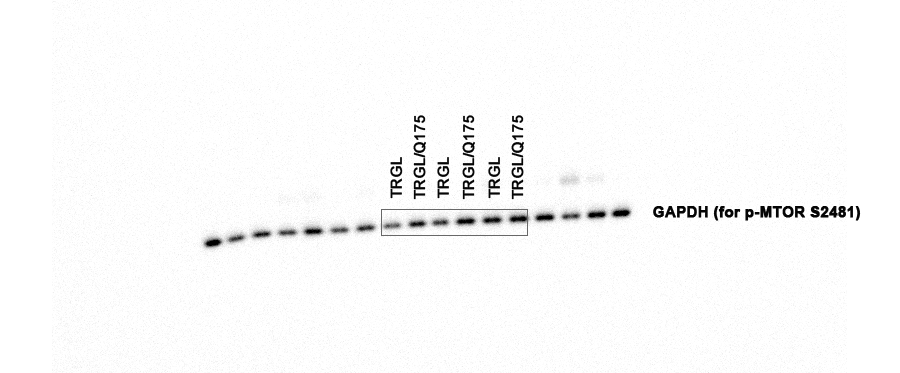

Supplement: Figure 3—figure supplement 1—source data 2. [file elife-104979-fig3-figsupp1-data2.zip › Figure 3-Figure supplement 1-Source Data 2/Anti-GAPDH (for p-MTOR S2481)_Short expos & Labeled.tif]

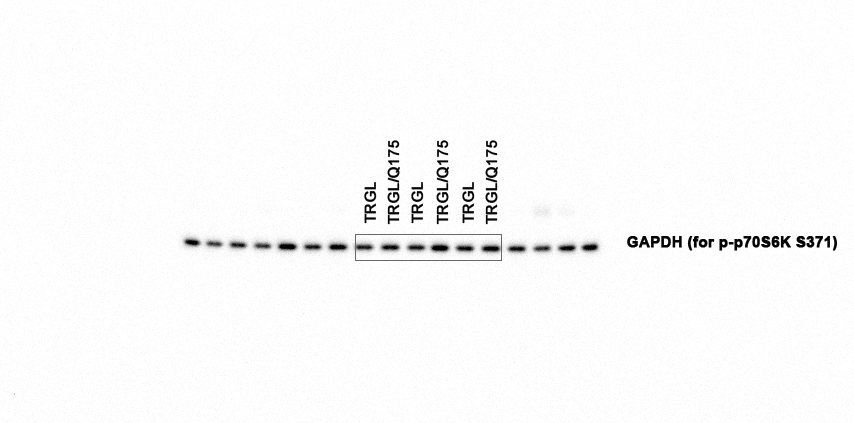

Supplement: Figure 3—figure supplement 1—source data 2. [file elife-104979-fig3-figsupp1-data2.zip › Figure 3-Figure supplement 1-Source Data 2/Anti-GAPDH (for p-p70S6K S371)_Short expos & Labeled.tif]

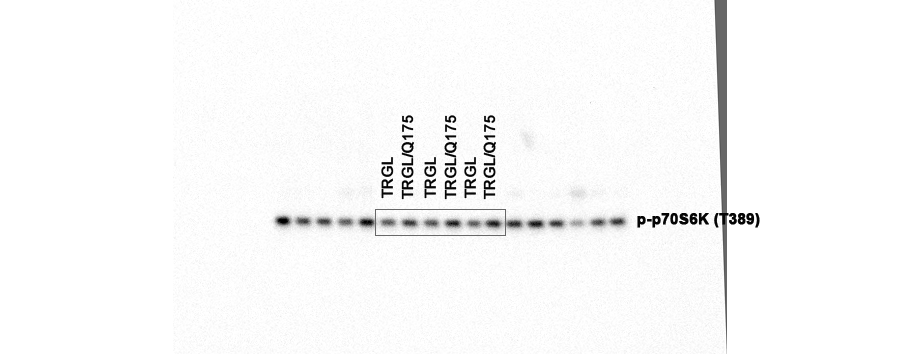

Supplement: Figure 3—figure supplement 1—source data 2. [file elife-104979-fig3-figsupp1-data2.zip › Figure 3-Figure supplement 1-Source Data 2/Anti-GAPDH (for p-p70S6K T389)_Short expos & Labeled.tif]

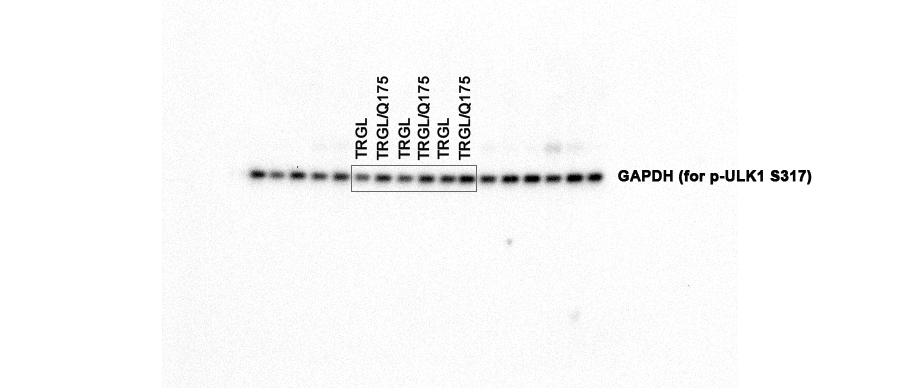

Supplement: Figure 3—figure supplement 1—source data 2. [file elife-104979-fig3-figsupp1-data2.zip › Figure 3-Figure supplement 1-Source Data 2/Anti-GAPDH (for p-ULK1 S317)_Labeled.tif]

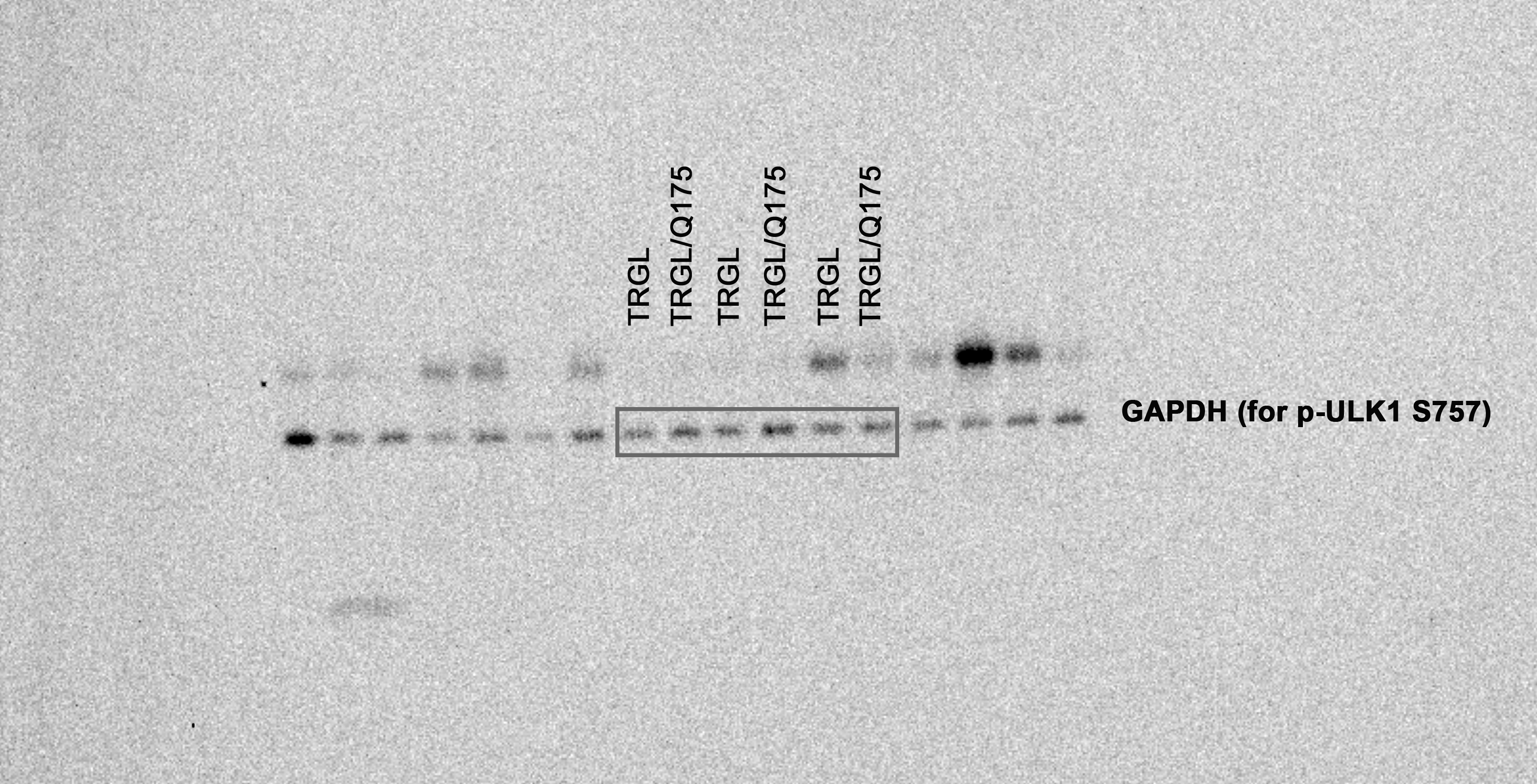

Supplement: Figure 3—figure supplement 1—source data 2. [file elife-104979-fig3-figsupp1-data2.zip › Figure 3-Figure supplement 1-Source Data 2/Anti-GAPDH (for p-ULK1 S757)_Labeled.tif]

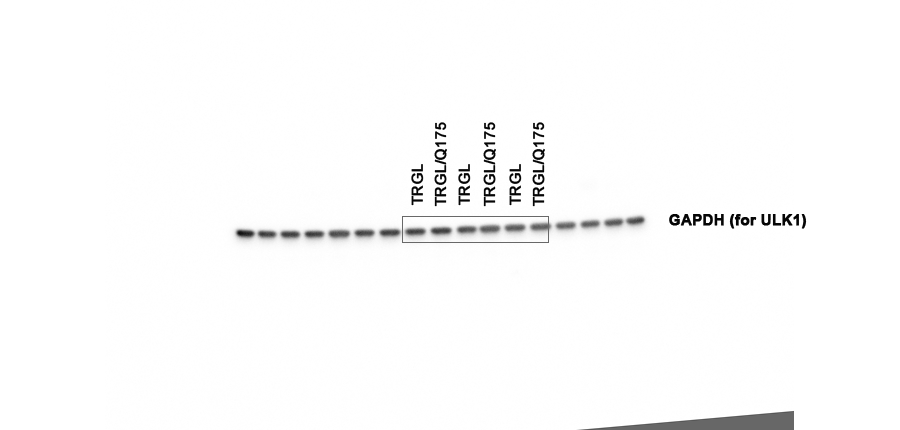

Supplement: Figure 3—figure supplement 1—source data 2. [file elife-104979-fig3-figsupp1-data2.zip › Figure 3-Figure supplement 1-Source Data 2/Anti-GAPDH (for ULK1)_Short expos & Labeled.tif]

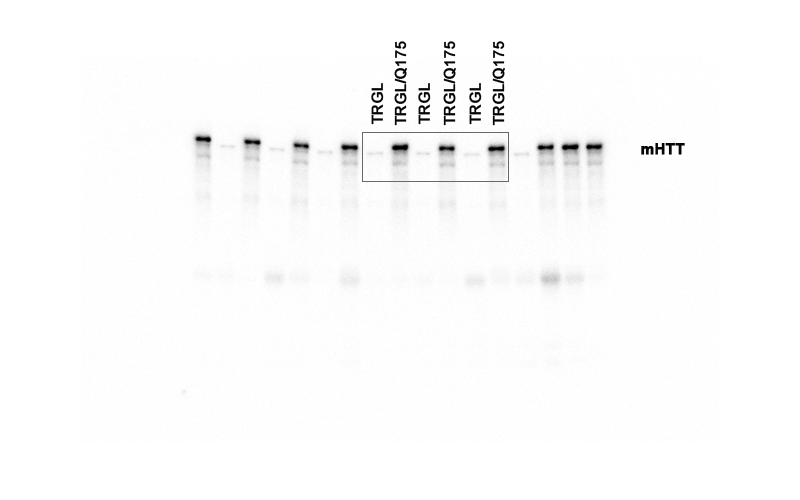

Supplement: Figure 3—figure supplement 1—source data 2. [file elife-104979-fig3-figsupp1-data2.zip › Figure 3-Figure supplement 1-Source Data 2/Anti-mHTT_Short expos & Labeled.tif]

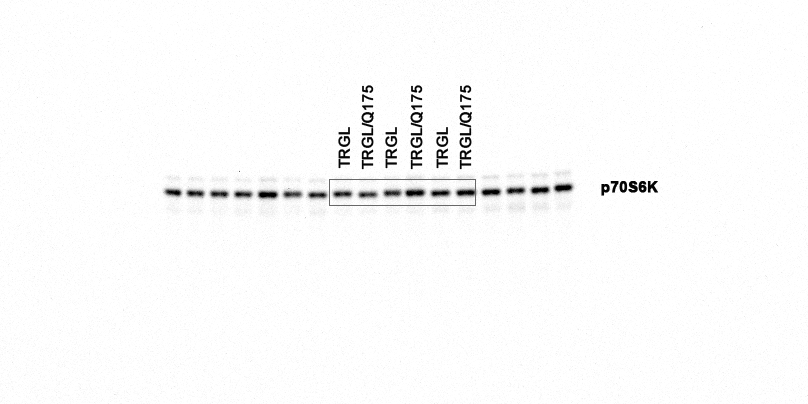

Supplement: Figure 3—figure supplement 1—source data 2. [file elife-104979-fig3-figsupp1-data2.zip › Figure 3-Figure supplement 1-Source Data 2/Anti-P70S6K_Short expos & Labeled.tif]

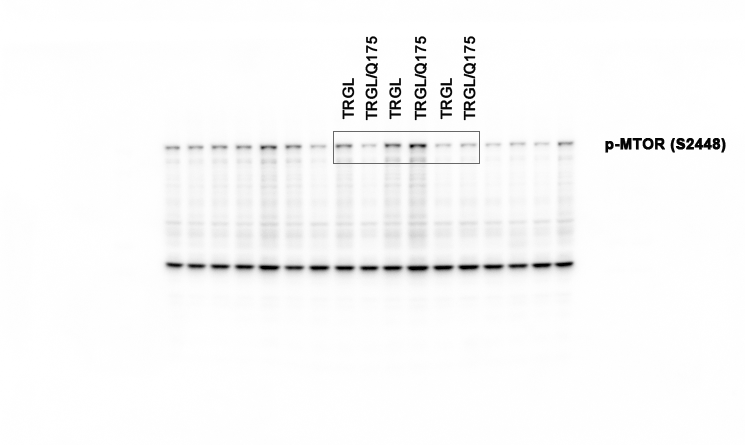

Supplement: Figure 3—figure supplement 1—source data 2. [file elife-104979-fig3-figsupp1-data2.zip › Figure 3-Figure supplement 1-Source Data 2/Anti-p-MTOR (S2448)_Short expos & Labeled.tif]

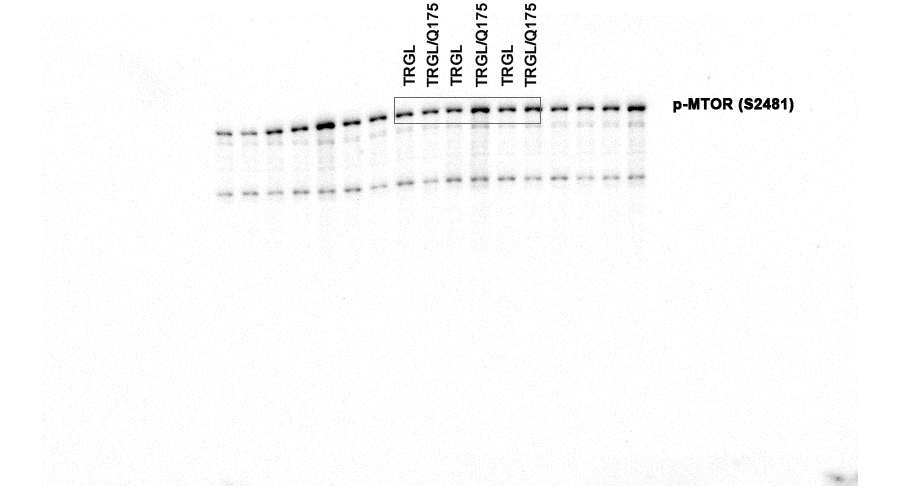

Supplement: Figure 3—figure supplement 1—source data 2. [file elife-104979-fig3-figsupp1-data2.zip › Figure 3-Figure supplement 1-Source Data 2/Anti-p-MTOR (S2481)_Short expos & Labeled.tif]

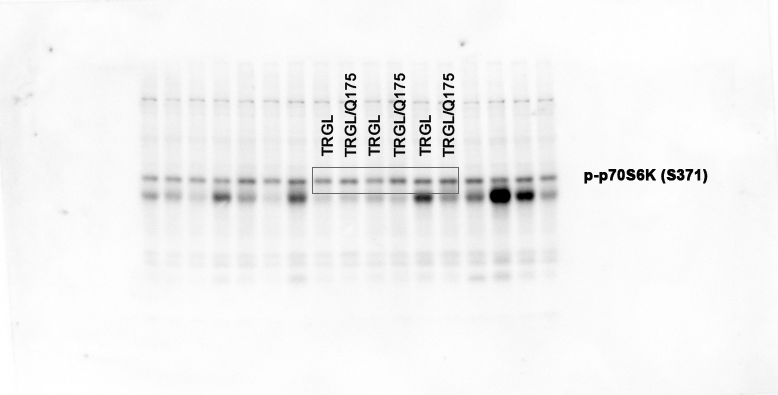

Supplement: Figure 3—figure supplement 1—source data 2. [file elife-104979-fig3-figsupp1-data2.zip › Figure 3-Figure supplement 1-Source Data 2/Anti-p-p70S6K (S371)_Short expos & Labeled.tif]

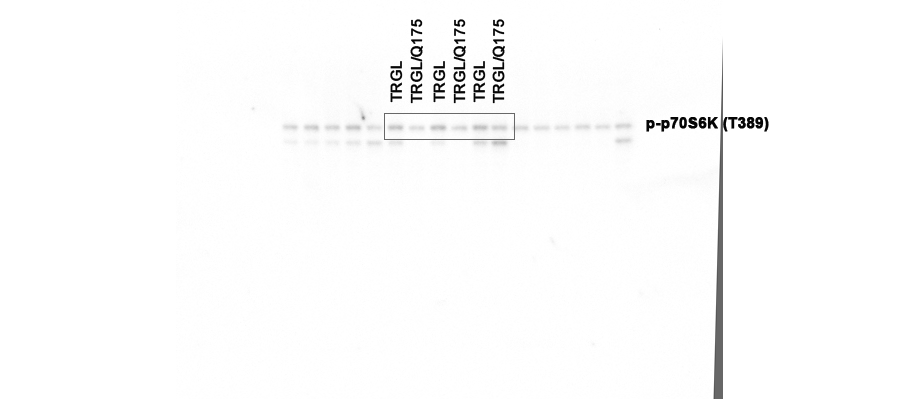

Supplement: Figure 3—figure supplement 1—source data 2. [file elife-104979-fig3-figsupp1-data2.zip › Figure 3-Figure supplement 1-Source Data 2/Anti-p-p70S6K (T389)_Short expos & Labeled.tif]

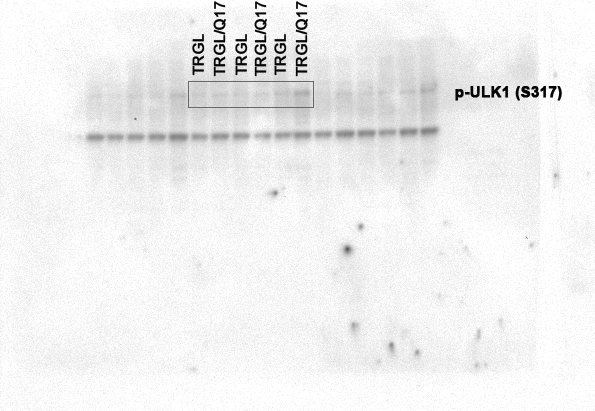

Supplement: Figure 3—figure supplement 1—source data 2. [file elife-104979-fig3-figsupp1-data2.zip › Figure 3-Figure supplement 1-Source Data 2/Anti-p-ULK1 (S317)_Labeled (adjusted).tif]

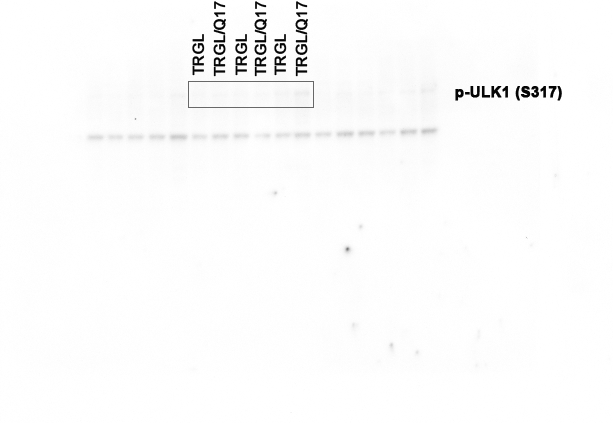

Supplement: Figure 3—figure supplement 1—source data 2. [file elife-104979-fig3-figsupp1-data2.zip › Figure 3-Figure supplement 1-Source Data 2/Anti-p-ULK1 (S317)_Labeled.tif]

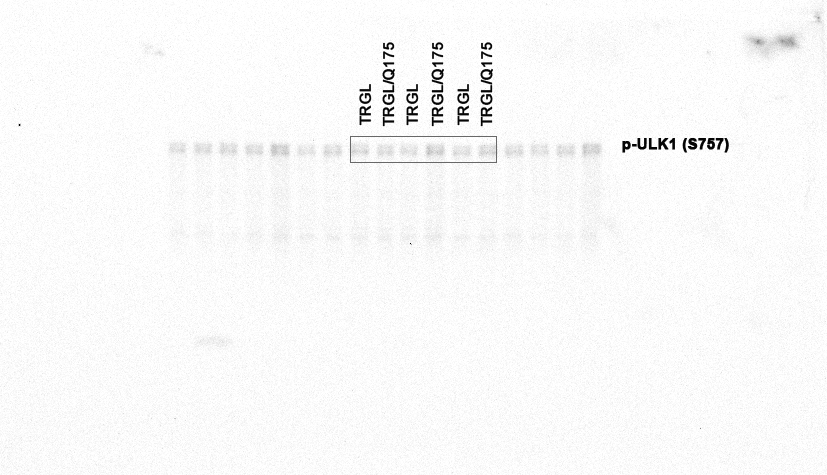

Supplement: Figure 3—figure supplement 1—source data 2. [file elife-104979-fig3-figsupp1-data2.zip › Figure 3-Figure supplement 1-Source Data 2/Anti-p-ULK1 (S757)_Labeled.tif]

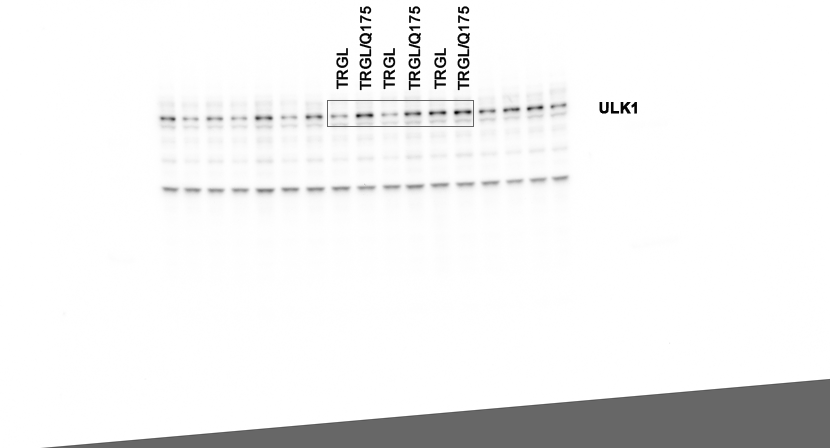

Supplement: Figure 3—figure supplement 1—source data 2. [file elife-104979-fig3-figsupp1-data2.zip › Figure 3-Figure supplement 1-Source Data 2/Anti-ULK1_Short expos & Labeled.tif]

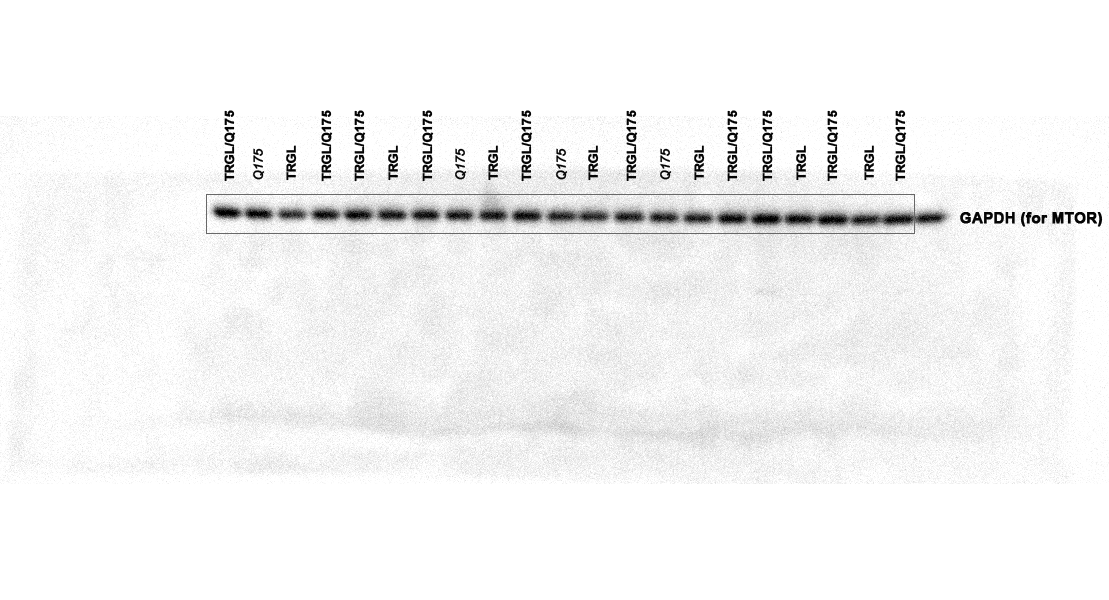

Supplement: Figure 3—figure supplement 1—source data 2. [file elife-104979-fig3-figsupp1-data2.zip › Figure 3-Figure supplement 1-Source Data 2/MTOR only/Anti-GAPDH (for MTOR used for quantitation-only)_Short expos & Labeled.tif]

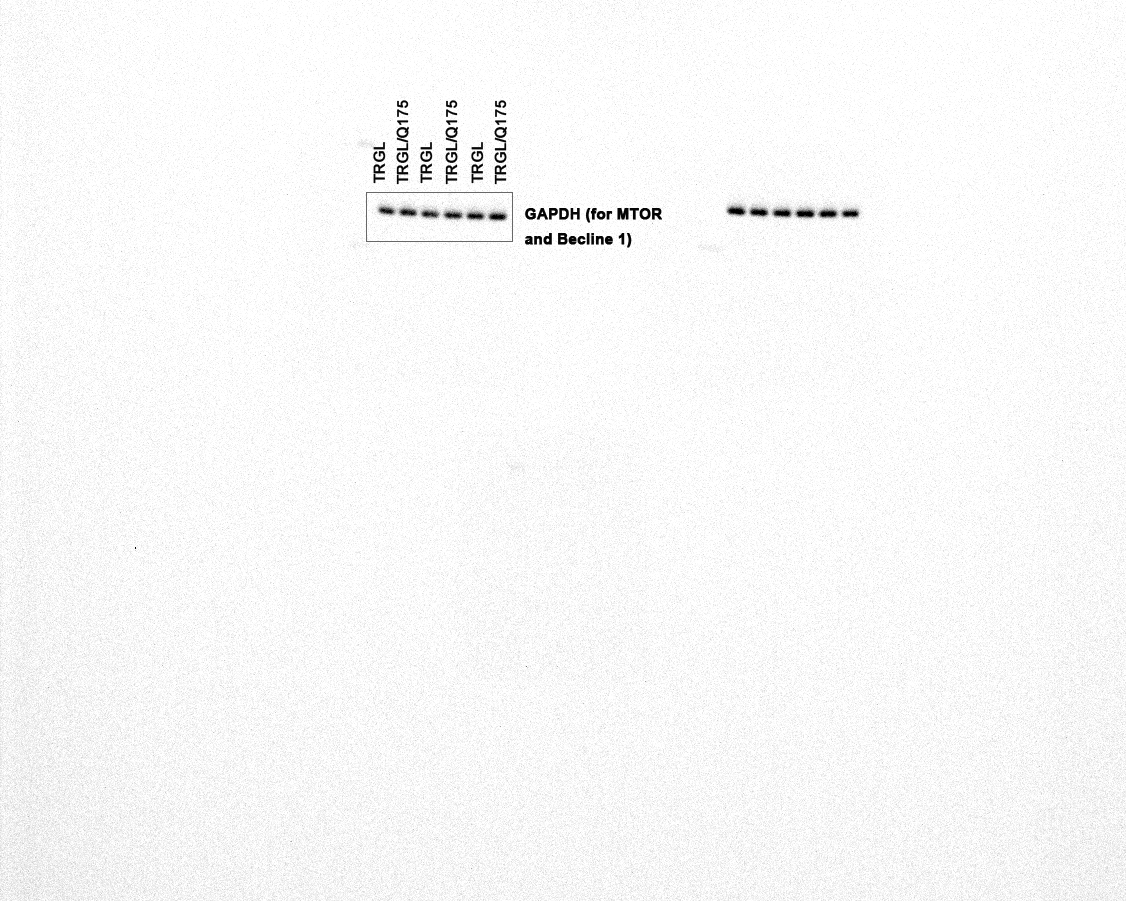

Supplement: Figure 3—figure supplement 1—source data 2. [file elife-104979-fig3-figsupp1-data2.zip › Figure 3-Figure supplement 1-Source Data 2/MTOR only/Anti-GAPDH (upper left, for MTOR & Beclin1 used for display-only)_Short expos & Labeled.tif]

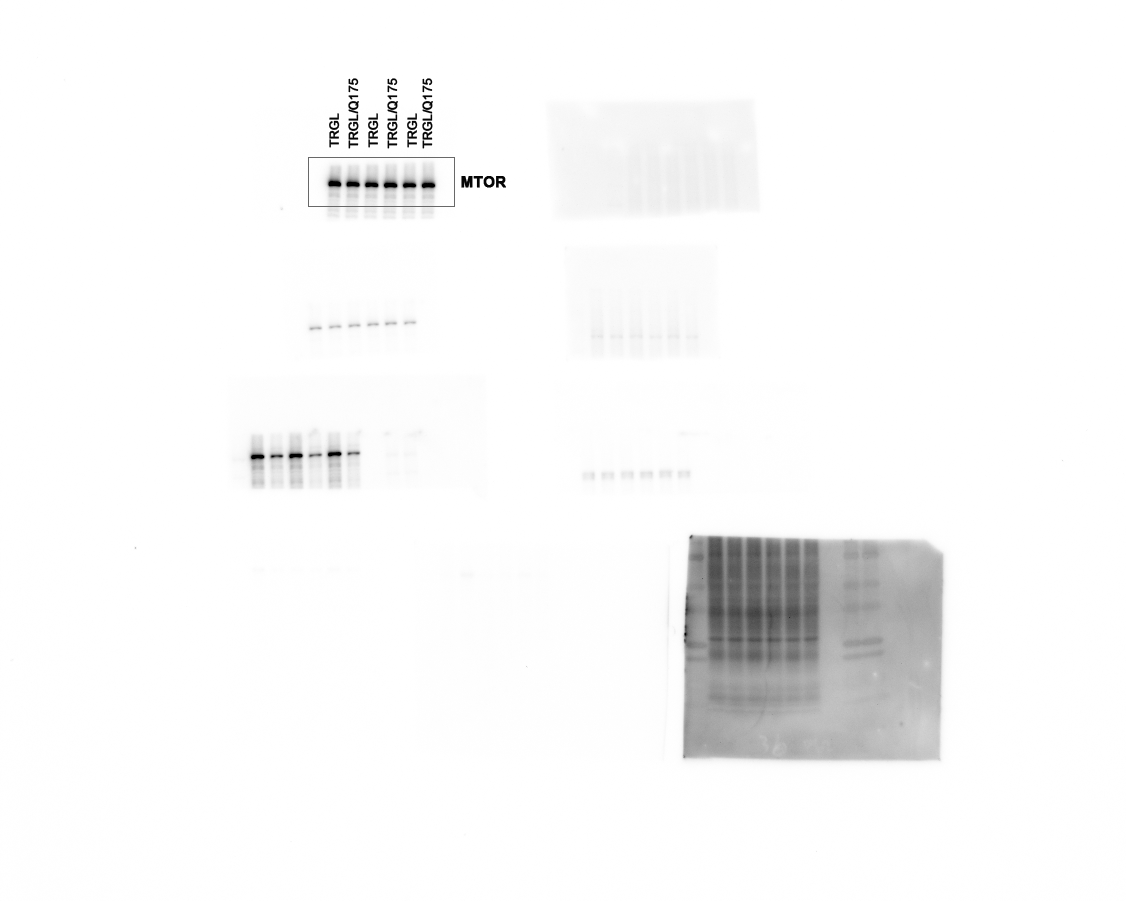

Supplement: Figure 3—figure supplement 1—source data 2. [file elife-104979-fig3-figsupp1-data2.zip › Figure 3-Figure supplement 1-Source Data 2/MTOR only/Anti-MTOR (upper-left, used for display-only)_Short expos & Labeled.tif]

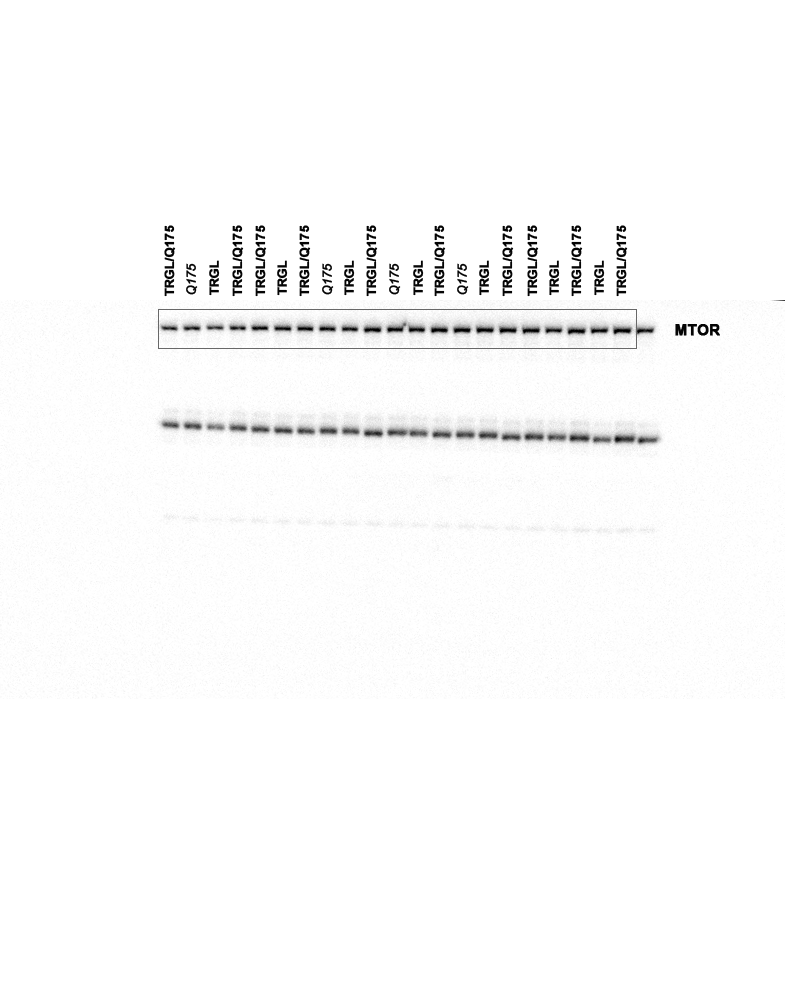

Supplement: Figure 3—figure supplement 1—source data 2. [file elife-104979-fig3-figsupp1-data2.zip › Figure 3-Figure supplement 1-Source Data 2/MTOR only/Anti-MTOR (used for quantitation-only)_Short expos & Labeled.tif]

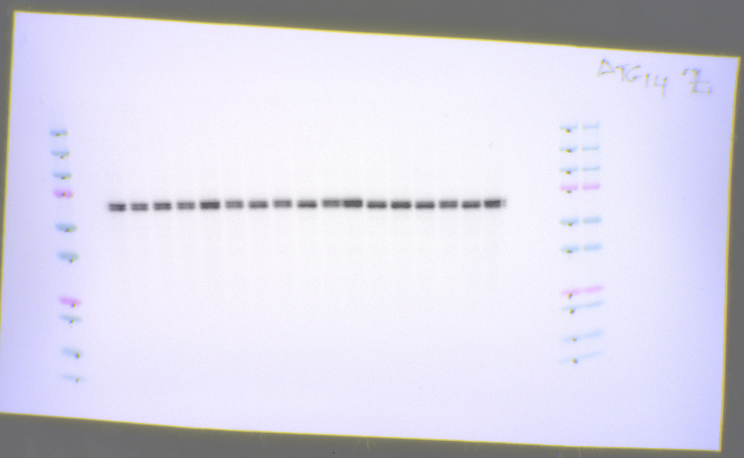

Supplement: Figure 3—figure supplement 2—source data 1. [file elife-104979-fig3-figsupp2-data1.zip › Figure 3-Figure supplement 2-Source Data 1/Anti-ATG14_Overlayed.tif]

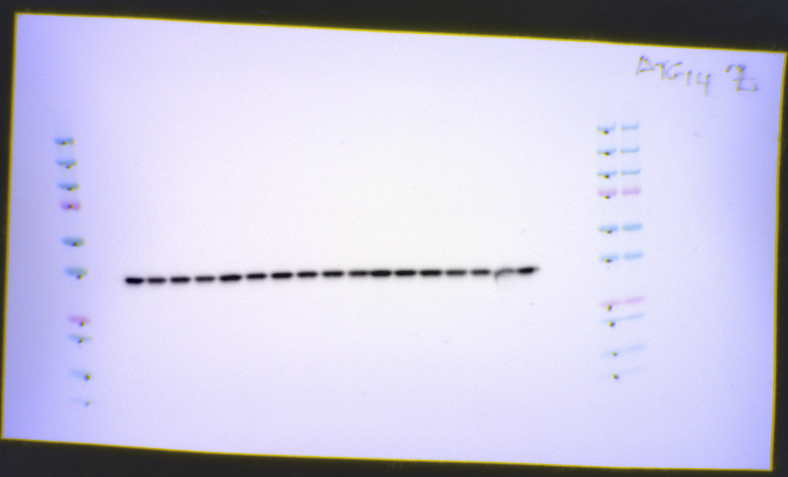

Supplement: Figure 3—figure supplement 2—source data 1. [file elife-104979-fig3-figsupp2-data1.zip › Figure 3-Figure supplement 2-Source Data 1/Anti-GAPDH (for ATG14)_Overlayed.tif]

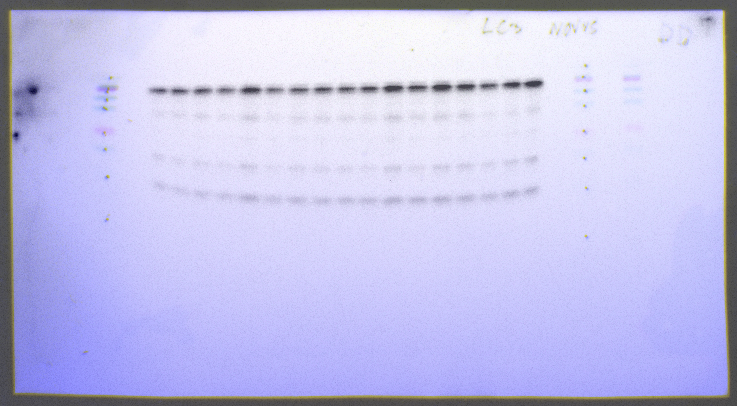

Supplement: Figure 3—figure supplement 2—source data 1. [file elife-104979-fig3-figsupp2-data1.zip › Figure 3-Figure supplement 2-Source Data 1/Anti-GAPDH (for LC3 Novus)_Overlayed.tif]

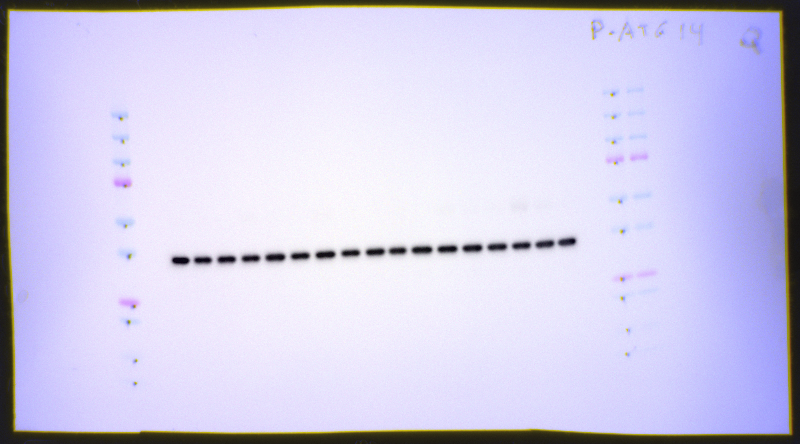

Supplement: Figure 3—figure supplement 2—source data 1. [file elife-104979-fig3-figsupp2-data1.zip › Figure 3-Figure supplement 2-Source Data 1/Anti-GAPDH (for p-ATG14 S29)_Overlayed.tif]

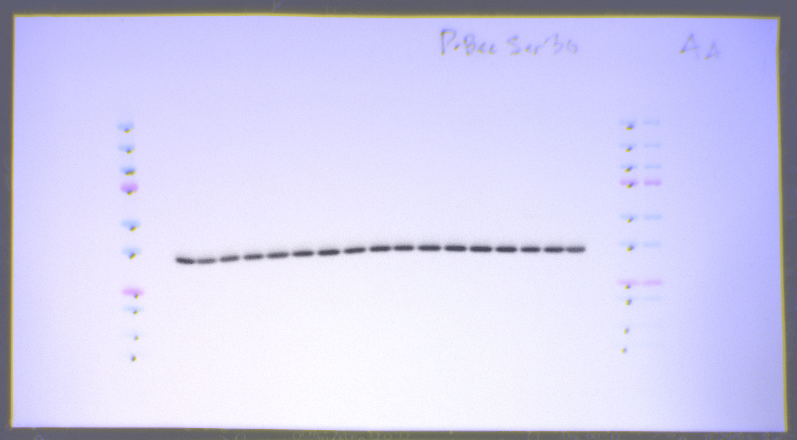

Supplement: Figure 3—figure supplement 2—source data 1. [file elife-104979-fig3-figsupp2-data1.zip › Figure 3-Figure supplement 2-Source Data 1/Anti-GAPDH (for p-Beclin1 S30)_Overlayed.tif]

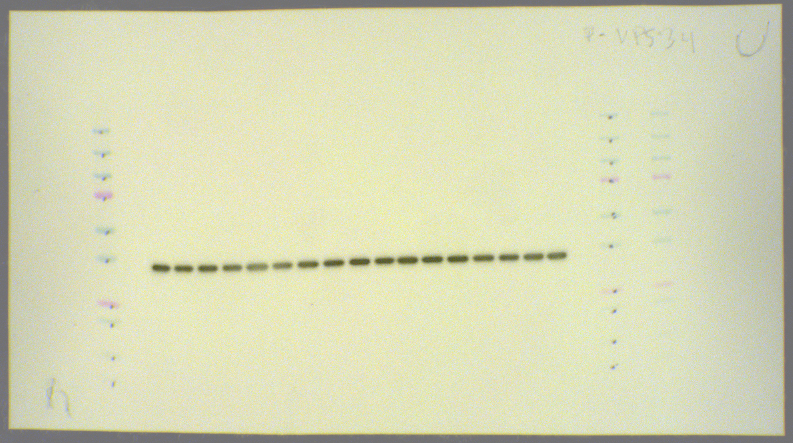

Supplement: Figure 3—figure supplement 2—source data 1. [file elife-104979-fig3-figsupp2-data1.zip › Figure 3-Figure supplement 2-Source Data 1/Anti-GAPDH (for p-VPS34 S249)_Overlayed.tif]

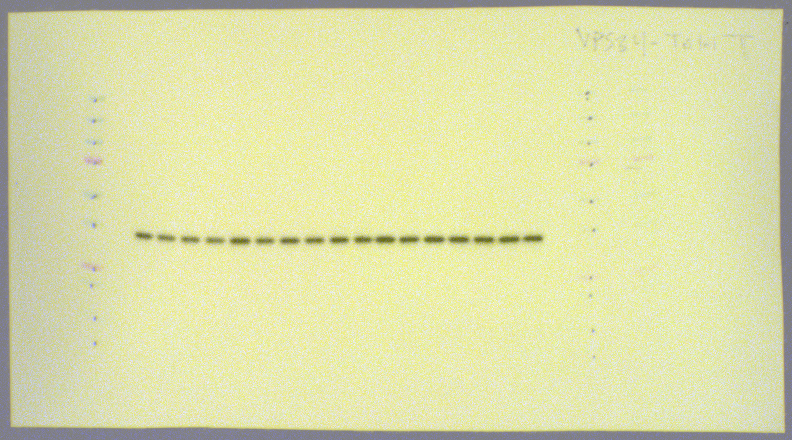

Supplement: Figure 3—figure supplement 2—source data 1. [file elife-104979-fig3-figsupp2-data1.zip › Figure 3-Figure supplement 2-Source Data 1/Anti-GAPDH (for VPS34)_Overlayed.tif]

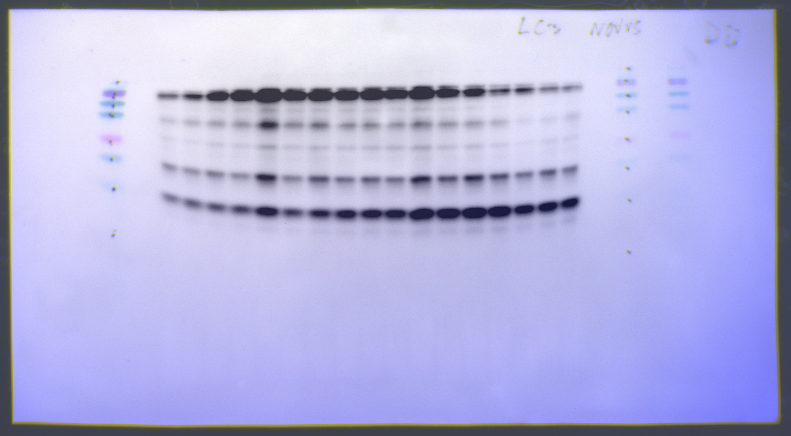

Supplement: Figure 3—figure supplement 2—source data 1. [file elife-104979-fig3-figsupp2-data1.zip › Figure 3-Figure supplement 2-Source Data 1/Anti-LC3 (Novus)_Overlayed.tif]

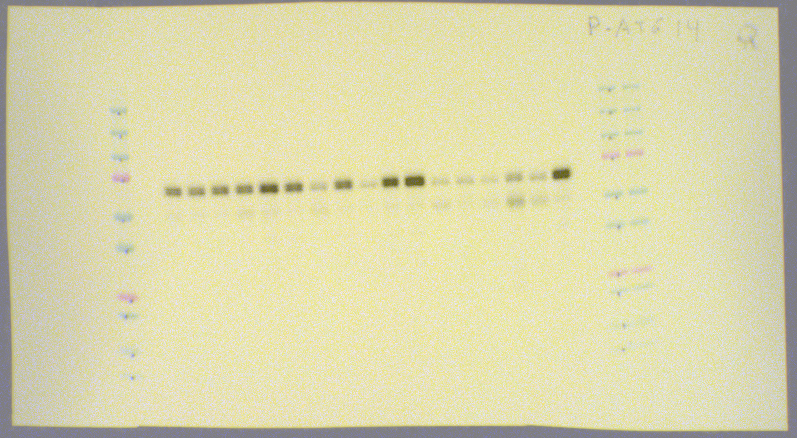

Supplement: Figure 3—figure supplement 2—source data 1. [file elife-104979-fig3-figsupp2-data1.zip › Figure 3-Figure supplement 2-Source Data 1/Anti-p-ATG14 (S29)_Overlayed.tif]

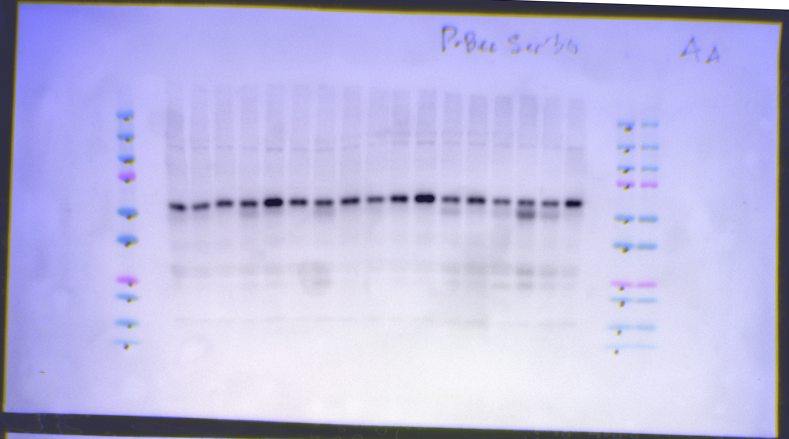

Supplement: Figure 3—figure supplement 2—source data 1. [file elife-104979-fig3-figsupp2-data1.zip › Figure 3-Figure supplement 2-Source Data 1/Anti-p-Beclin1 (S30)_Overlayed.tif]

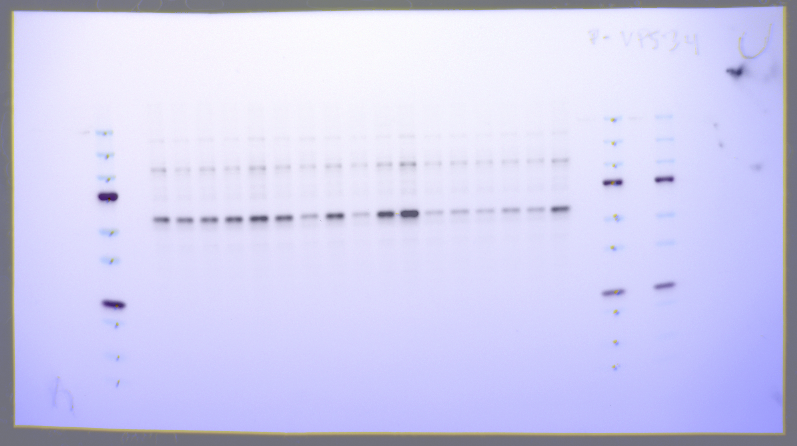

Supplement: Figure 3—figure supplement 2—source data 1. [file elife-104979-fig3-figsupp2-data1.zip › Figure 3-Figure supplement 2-Source Data 1/Anti-p-VPS34 (S249)_Overlayed.tif]

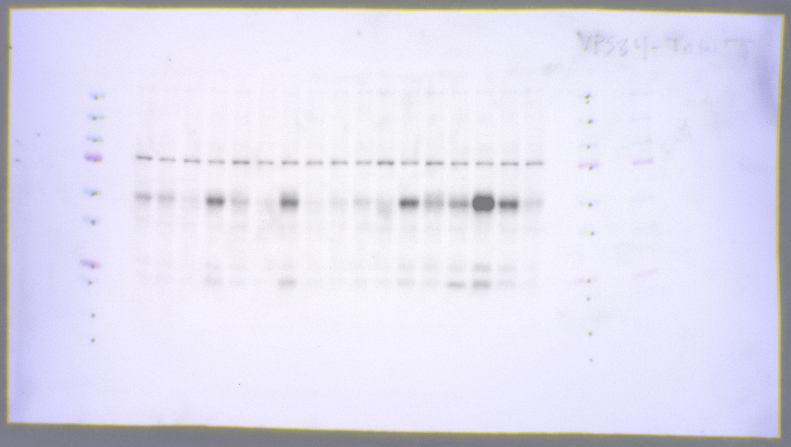

Supplement: Figure 3—figure supplement 2—source data 1. [file elife-104979-fig3-figsupp2-data1.zip › Figure 3-Figure supplement 2-Source Data 1/Anti-VPS34_Overlayed.tif]

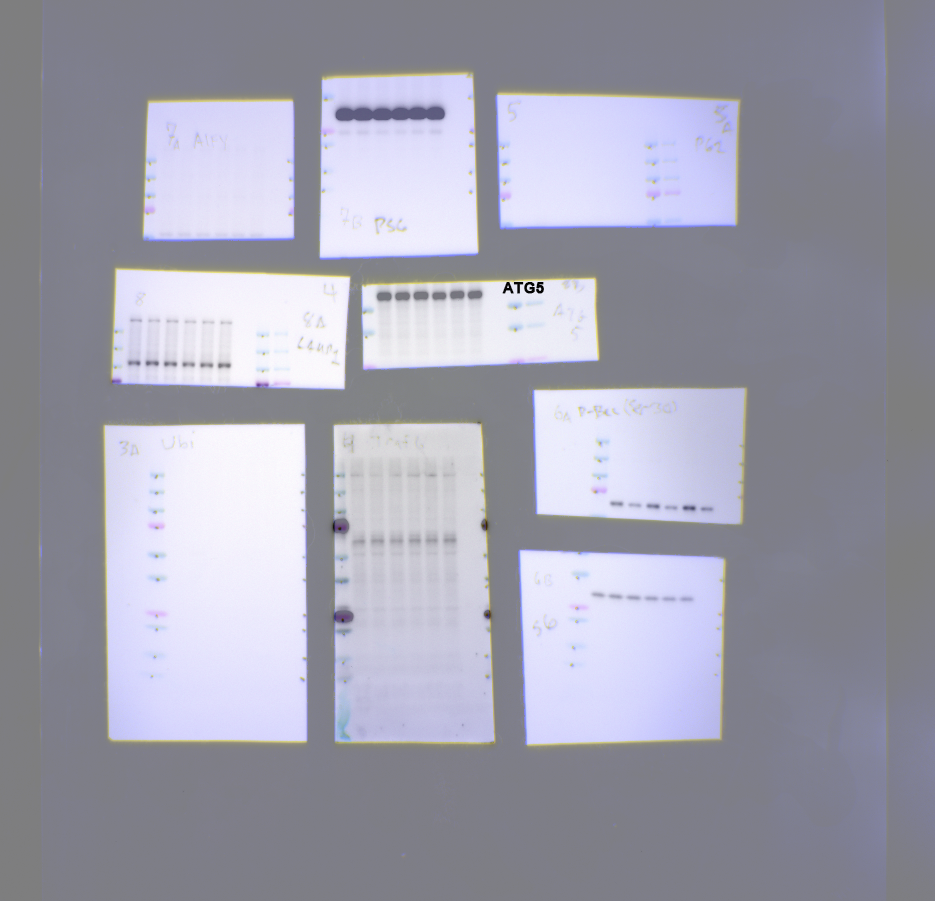

Supplement: Figure 3—figure supplement 2—source data 1. [file elife-104979-fig3-figsupp2-data1.zip › Figure 3-Figure supplement 2-Source Data 1/Beclin 1 & Atg5 only/Anti-ATG5 (mid-right, used for display-only)_Overlayed.tif]

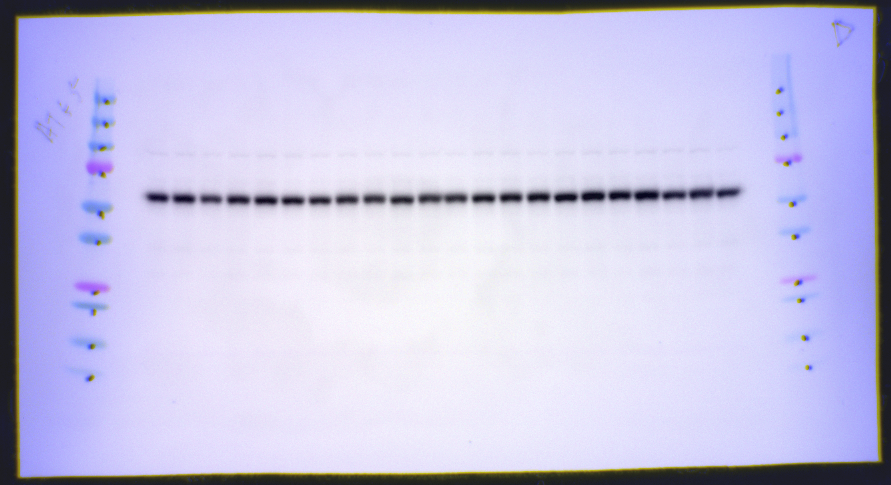

Supplement: Figure 3—figure supplement 2—source data 1. [file elife-104979-fig3-figsupp2-data1.zip › Figure 3-Figure supplement 2-Source Data 1/Beclin 1 & Atg5 only/Anti-ATG5 (used for quantitation-only)_Overlayed.tif]

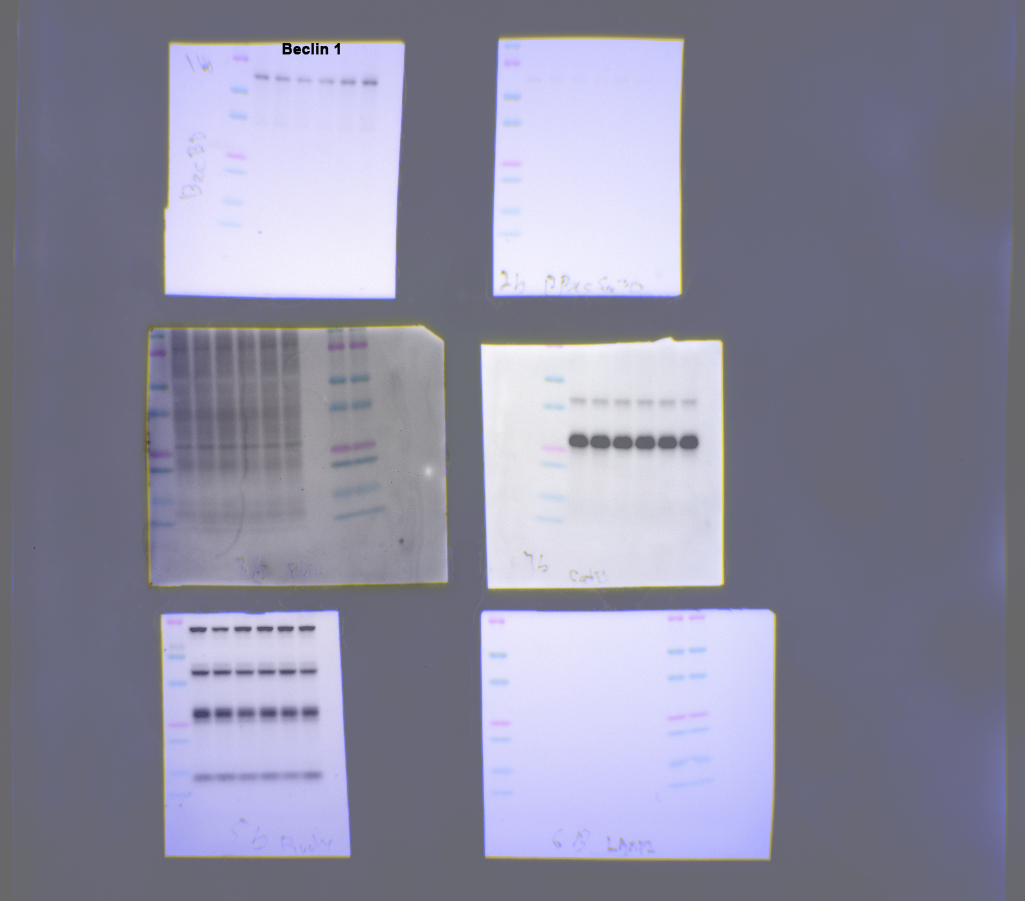

Supplement: Figure 3—figure supplement 2—source data 1. [file elife-104979-fig3-figsupp2-data1.zip › Figure 3-Figure supplement 2-Source Data 1/Beclin 1 & Atg5 only/Anti-Beclin1 (top left, used for display-only)_Overlayed.tif]

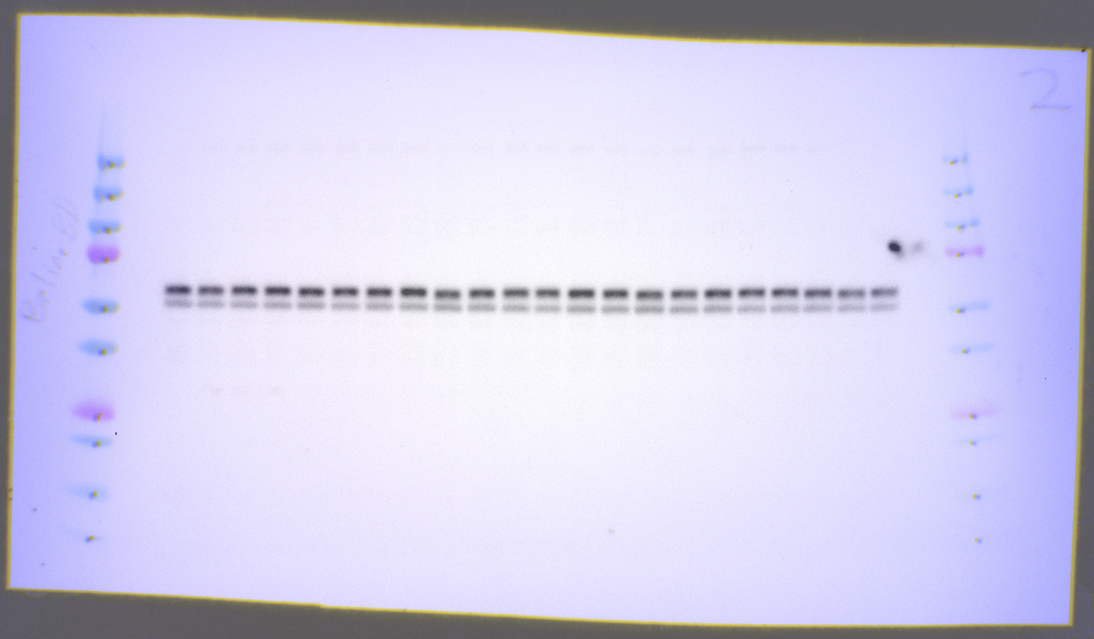

Supplement: Figure 3—figure supplement 2—source data 1. [file elife-104979-fig3-figsupp2-data1.zip › Figure 3-Figure supplement 2-Source Data 1/Beclin 1 & Atg5 only/Anti-Beclin1 (used for quantitation-only)_Overlayed.tif]

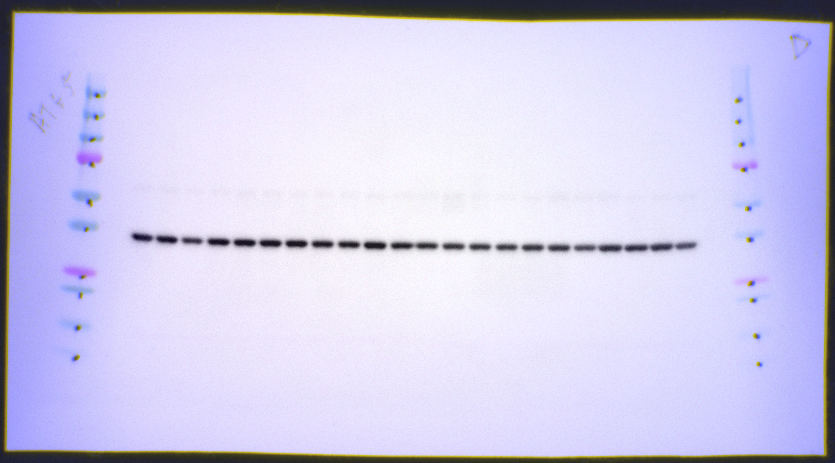

Supplement: Figure 3—figure supplement 2—source data 1. [file elife-104979-fig3-figsupp2-data1.zip › Figure 3-Figure supplement 2-Source Data 1/Beclin 1 & Atg5 only/Anti-GAPDH (for Atg5 used for quantitation-only)_Overlayed.tif]

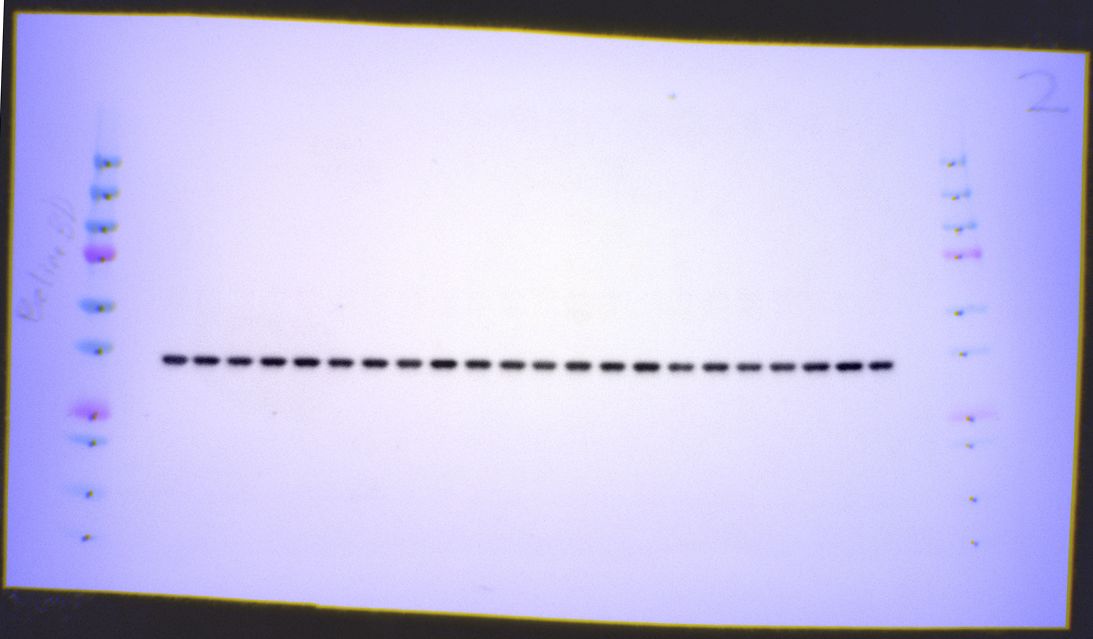

Supplement: Figure 3—figure supplement 2—source data 1. [file elife-104979-fig3-figsupp2-data1.zip › Figure 3-Figure supplement 2-Source Data 1/Beclin 1 & Atg5 only/Anti-GAPDH (for Beclin1 & CTSB used for quantitation-only)_Overlayed.tif]

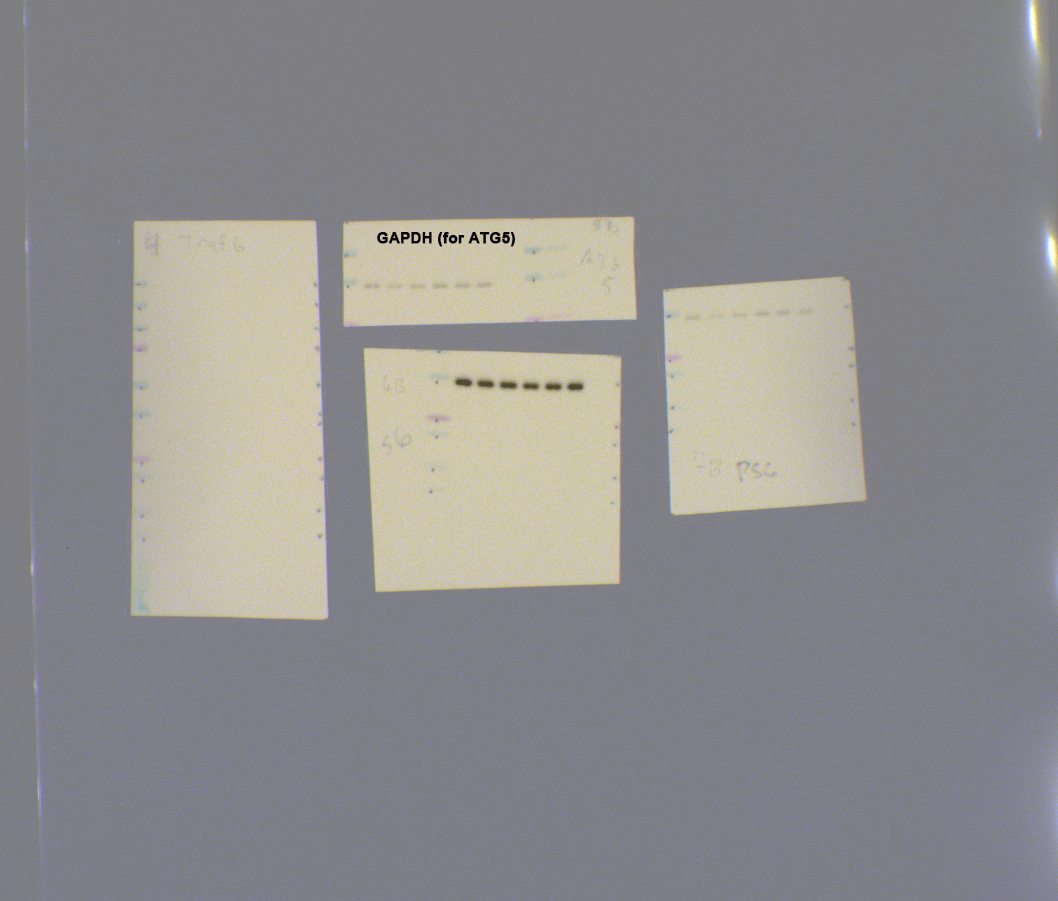

Supplement: Figure 3—figure supplement 2—source data 1. [file elife-104979-fig3-figsupp2-data1.zip › Figure 3-Figure supplement 2-Source Data 1/Beclin 1 & Atg5 only/Anti-GAPDH (mid-top, for ATG5 used for display-only)_Overlayed.tif]

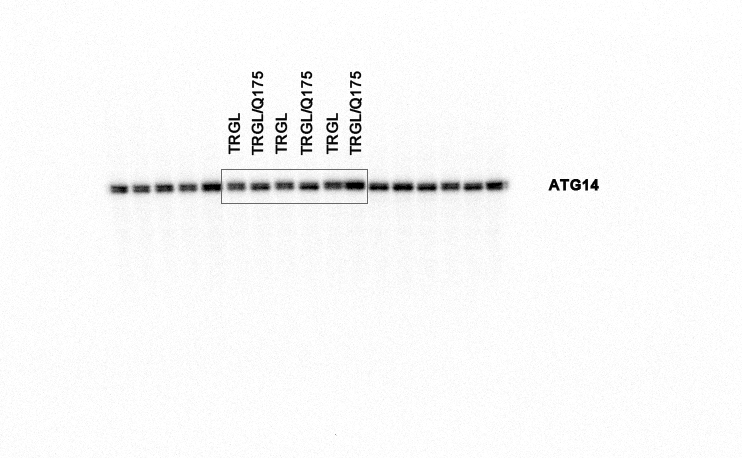

Supplement: Figure 3—figure supplement 2—source data 2. [file elife-104979-fig3-figsupp2-data2.zip › Figure 3-Figure supplement 2-Source Data 2/Anti-ATG14_Short expos & Labeled.tif]

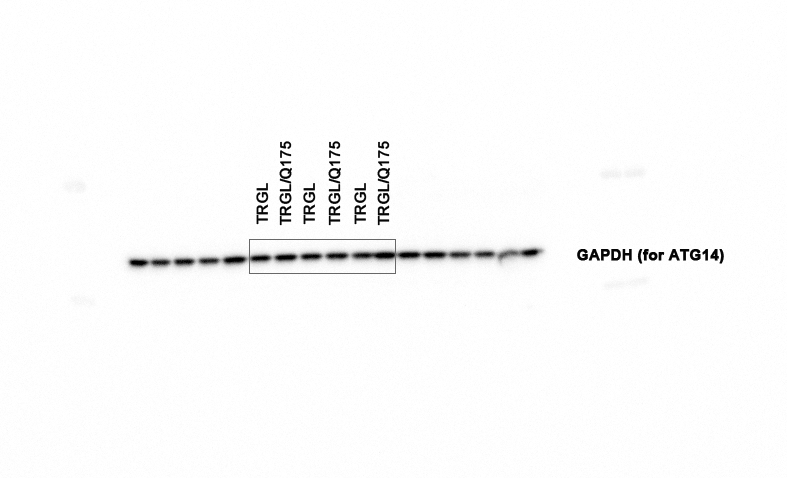

Supplement: Figure 3—figure supplement 2—source data 2. [file elife-104979-fig3-figsupp2-data2.zip › Figure 3-Figure supplement 2-Source Data 2/Anti-GAPDH (for ATG14)_Short expos & Labeled.tif]

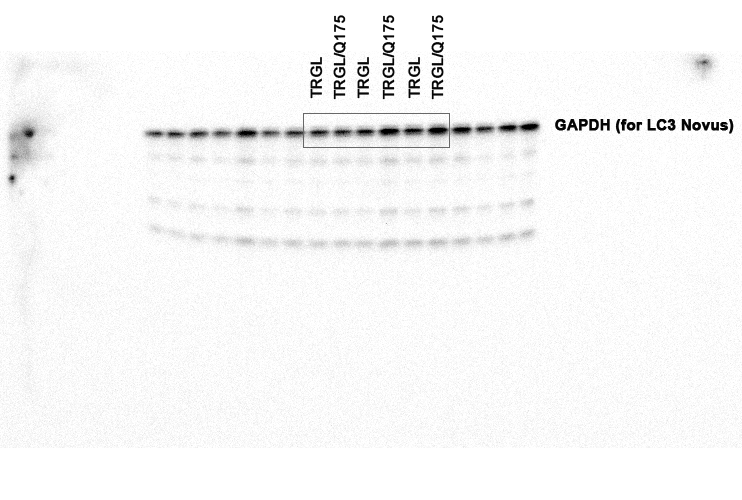

Supplement: Figure 3—figure supplement 2—source data 2. [file elife-104979-fig3-figsupp2-data2.zip › Figure 3-Figure supplement 2-Source Data 2/Anti-GAPDH (for LC3 Novus)_Short expos & Labeled.tif]

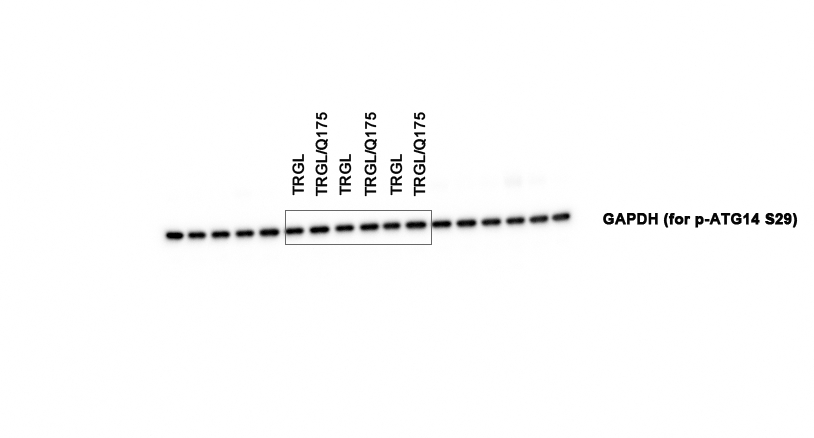

Supplement: Figure 3—figure supplement 2—source data 2. [file elife-104979-fig3-figsupp2-data2.zip › Figure 3-Figure supplement 2-Source Data 2/Anti-GAPDH (for p-ATG14 S29)_Short expos & Labeled.tif]

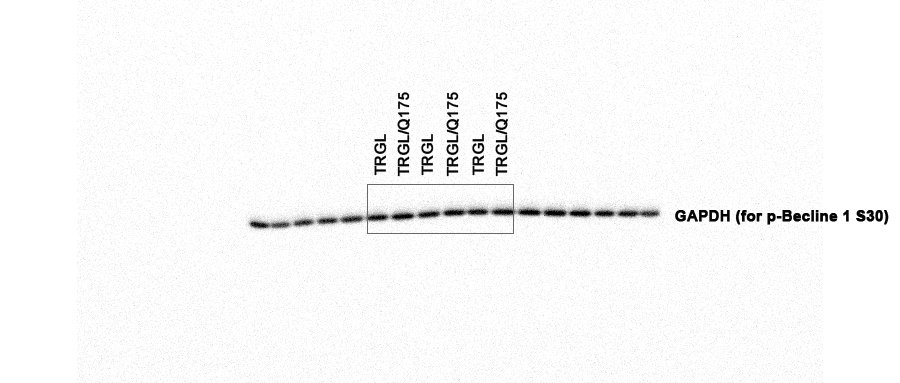

Supplement: Figure 3—figure supplement 2—source data 2. [file elife-104979-fig3-figsupp2-data2.zip › Figure 3-Figure supplement 2-Source Data 2/Anti-GAPDH (for p-Beclin1 S30)_Short expos & Labeled.tif]

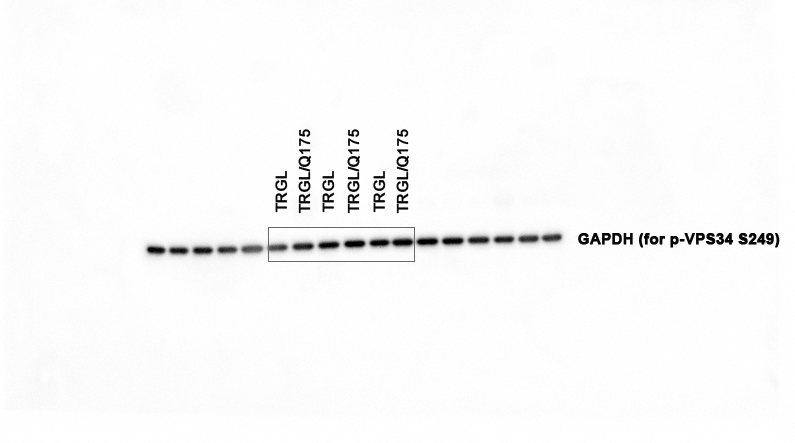

Supplement: Figure 3—figure supplement 2—source data 2. [file elife-104979-fig3-figsupp2-data2.zip › Figure 3-Figure supplement 2-Source Data 2/Anti-GAPDH (for p-VPS34 S249)_Short expos & Labeled.tif]

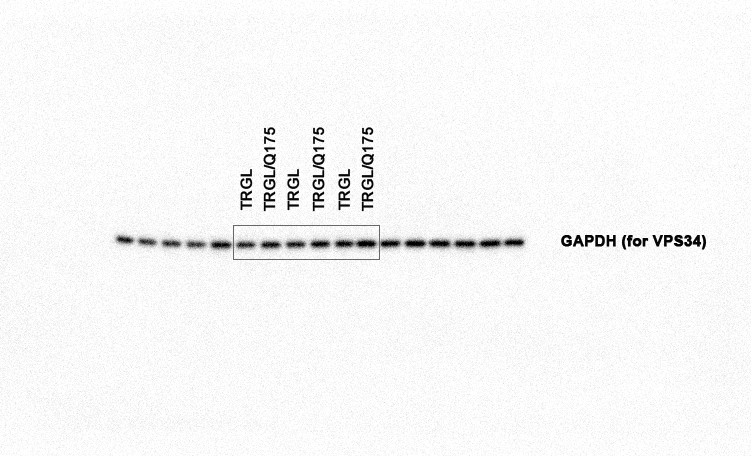

Supplement: Figure 3—figure supplement 2—source data 2. [file elife-104979-fig3-figsupp2-data2.zip › Figure 3-Figure supplement 2-Source Data 2/Anti-GAPDH (for VPS34)_Short expos & Labeled.tif]

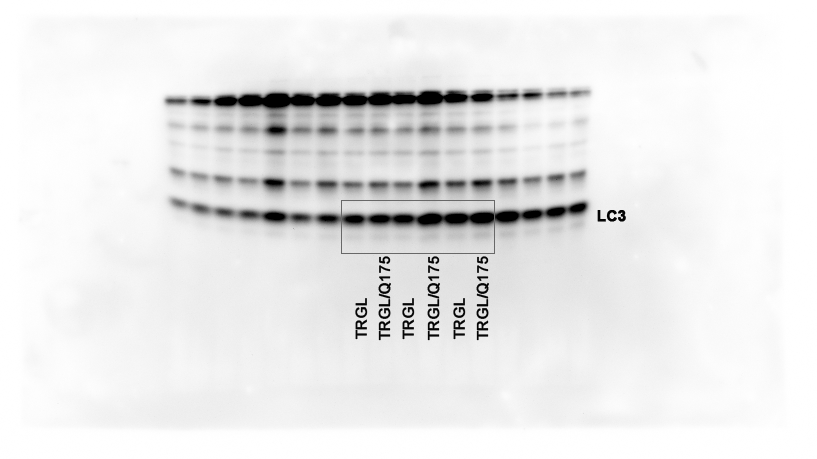

Supplement: Figure 3—figure supplement 2—source data 2. [file elife-104979-fig3-figsupp2-data2.zip › Figure 3-Figure supplement 2-Source Data 2/Anti-LC3 (Novus)_Labeled (Long expos).tif]

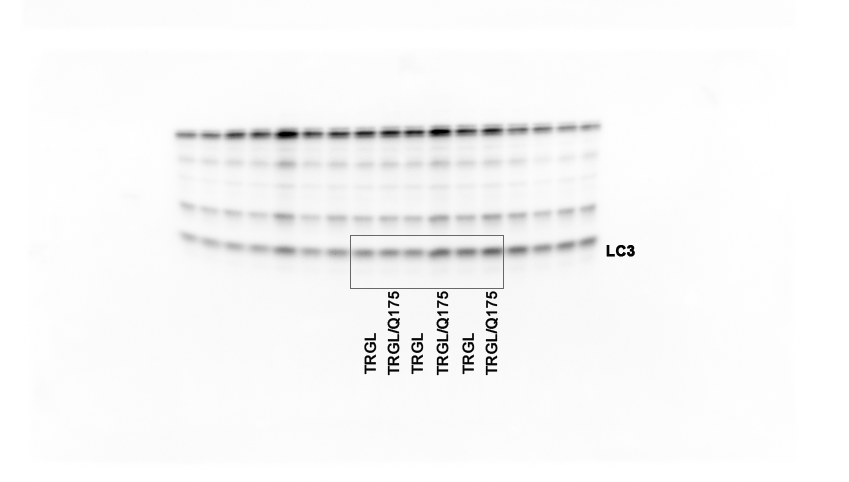

Supplement: Figure 3—figure supplement 2—source data 2. [file elife-104979-fig3-figsupp2-data2.zip › Figure 3-Figure supplement 2-Source Data 2/Anti-LC3 (Novus)_Labeled (Short expos).tif]

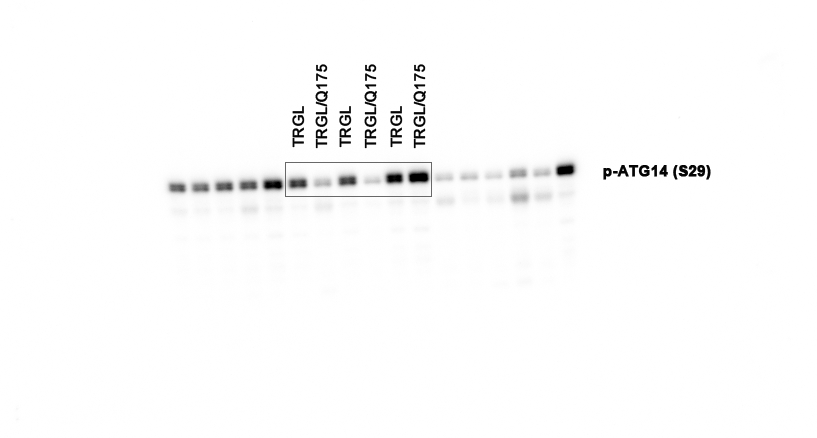

Supplement: Figure 3—figure supplement 2—source data 2. [file elife-104979-fig3-figsupp2-data2.zip › Figure 3-Figure supplement 2-Source Data 2/Anti-p-ATG14 (S29)_Short expos & Labeled.tif]

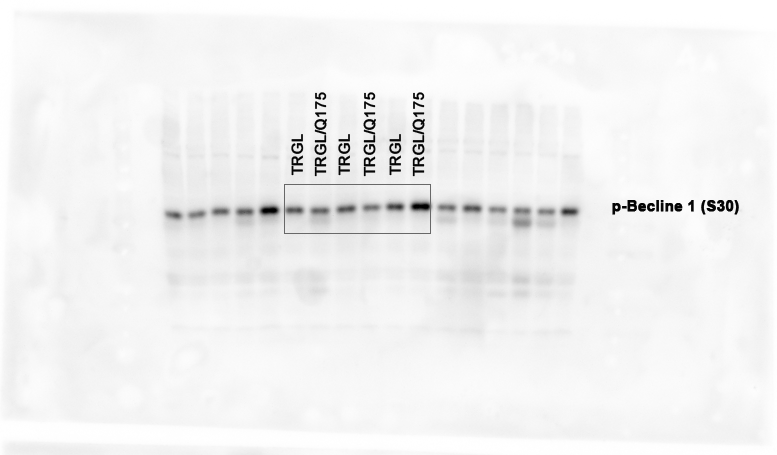

Supplement: Figure 3—figure supplement 2—source data 2. [file elife-104979-fig3-figsupp2-data2.zip › Figure 3-Figure supplement 2-Source Data 2/Anti-p-Beclin1 (S30)_Short expos & Labeled.tif]

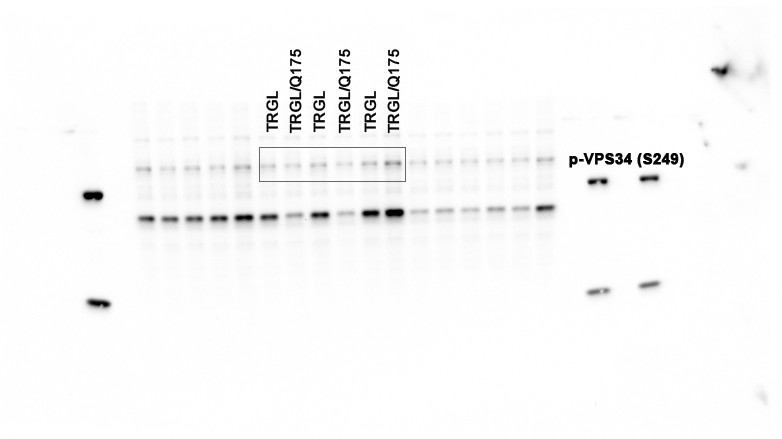

Supplement: Figure 3—figure supplement 2—source data 2. [file elife-104979-fig3-figsupp2-data2.zip › Figure 3-Figure supplement 2-Source Data 2/Anti-p-VPS34 (S249)_Short expos & Labeled.tif]

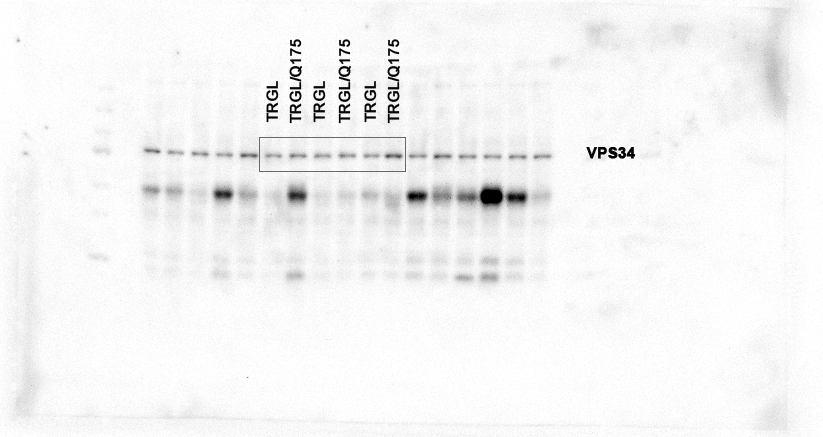

Supplement: Figure 3—figure supplement 2—source data 2. [file elife-104979-fig3-figsupp2-data2.zip › Figure 3-Figure supplement 2-Source Data 2/Anti-VPS34_Short expos & Labeled.tif]

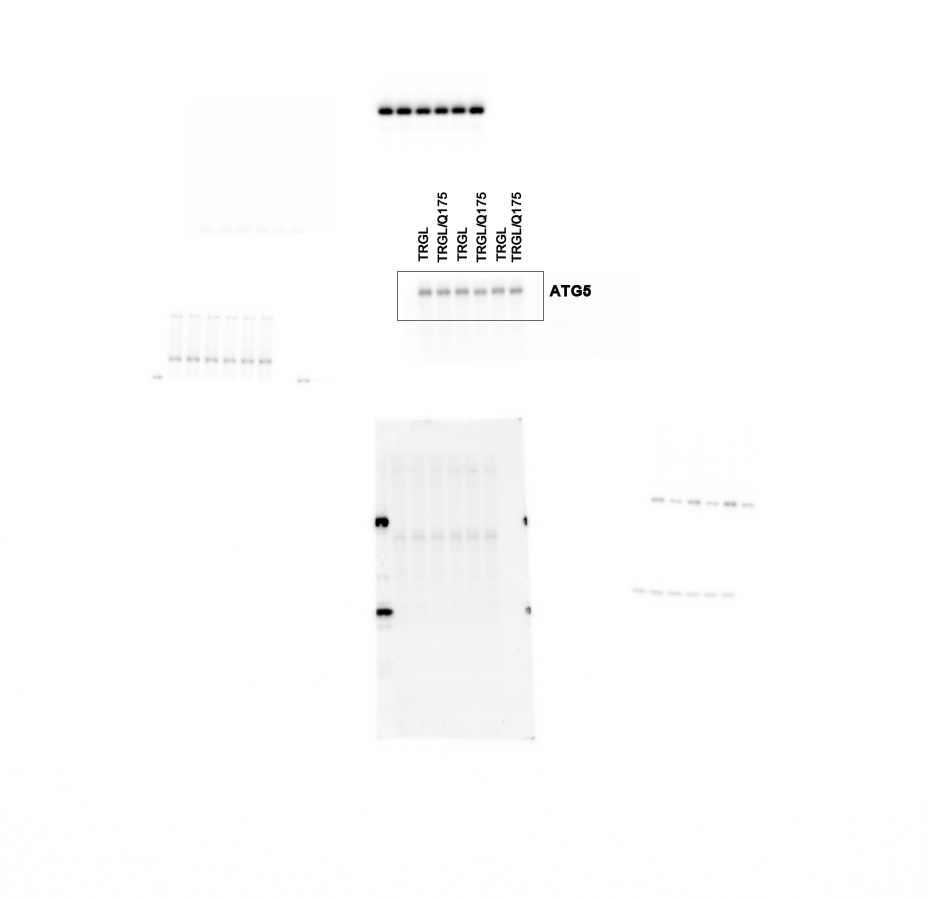

Supplement: Figure 3—figure supplement 2—source data 2. [file elife-104979-fig3-figsupp2-data2.zip › Figure 3-Figure supplement 2-Source Data 2/Beclin 1 & Atg5 only/Anti-ATG5 (mid-right, used for display-only)_Short expos & Labeled.tif]

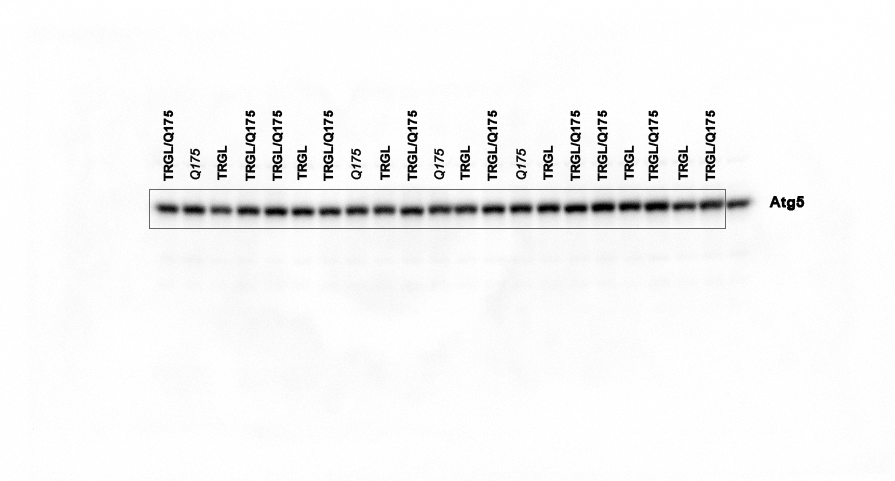

Supplement: Figure 3—figure supplement 2—source data 2. [file elife-104979-fig3-figsupp2-data2.zip › Figure 3-Figure supplement 2-Source Data 2/Beclin 1 & Atg5 only/Anti-ATG5 (used for quantitation-only)_Short expos & Labeled.tif]

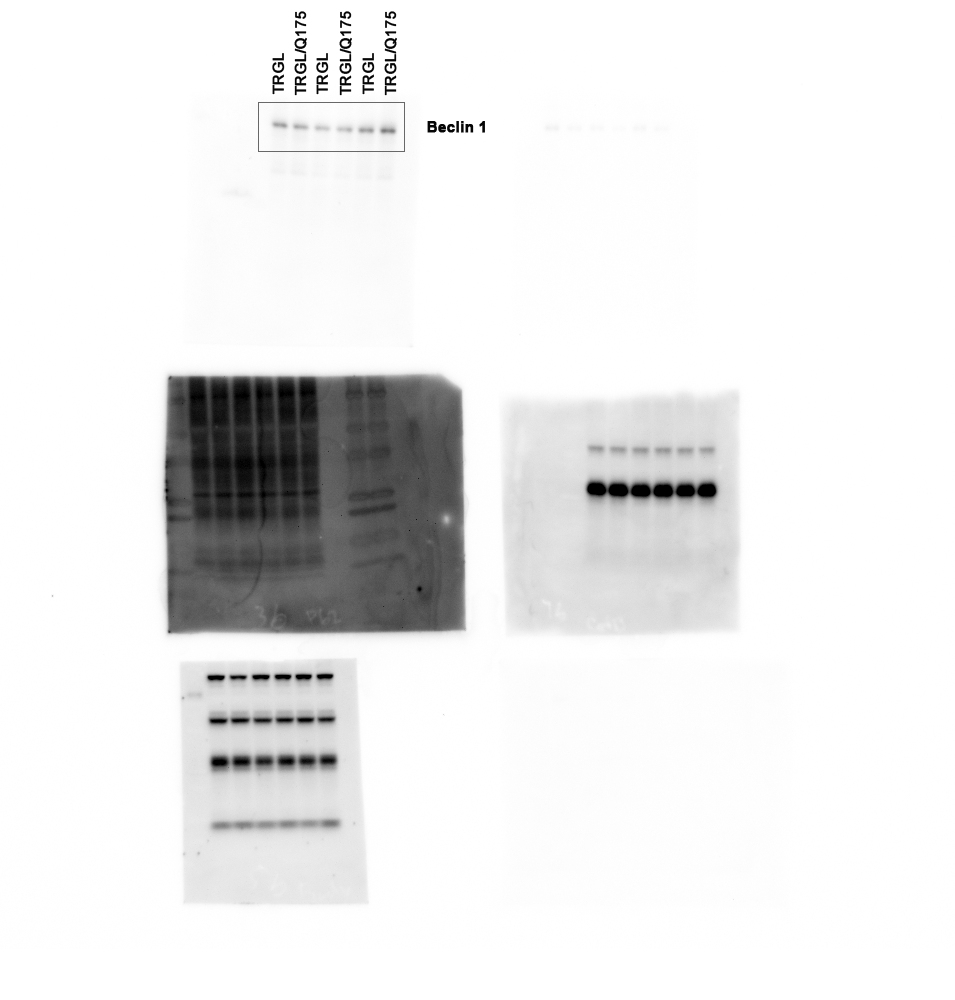

Supplement: Figure 3—figure supplement 2—source data 2. [file elife-104979-fig3-figsupp2-data2.zip › Figure 3-Figure supplement 2-Source Data 2/Beclin 1 & Atg5 only/Anti-Beclin1 (top left, used for display-only)_Short expos & Labeled.tif]
