# Supplementary figures and images for: mTOR inhibition in Q175 Huntington’s disease model mice facilitates neuronal autophagy and mutant huntingtin clearance (part 2 of 2)
Source: eLife. 2025 May 20;14:RP104979. doi: 10.7554/eLife.104979 (PMC12092004; doi:10.7554/eLife.104979)

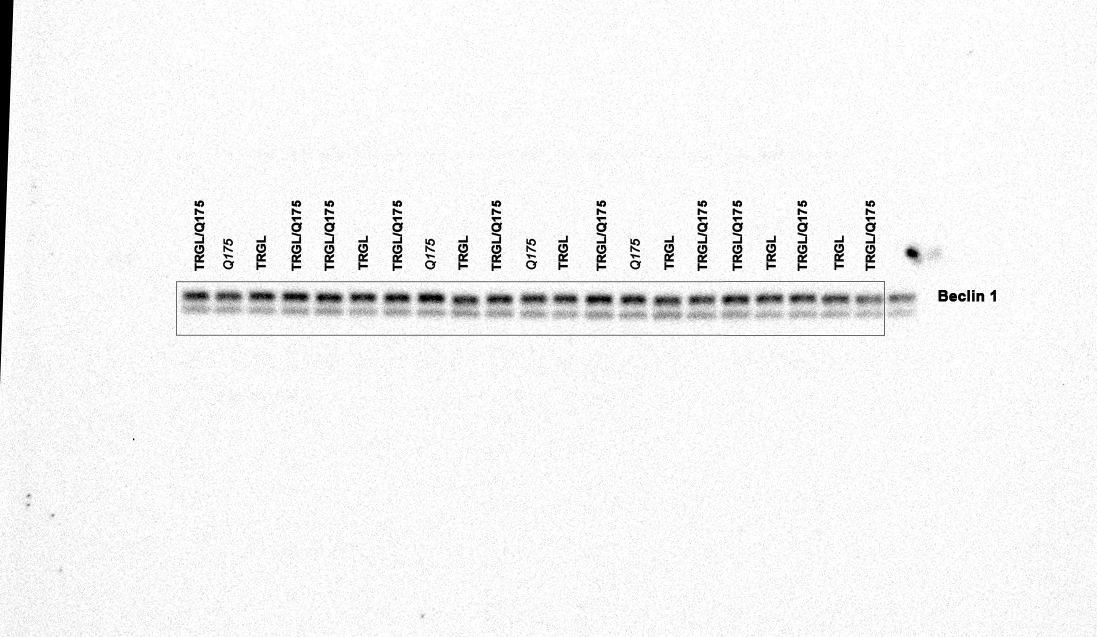

Supplement: Figure 3—figure supplement 2—source data 2. [file elife-104979-fig3-figsupp2-data2.zip › Figure 3-Figure supplement 2-Source Data 2/Beclin 1 & Atg5 only/Anti-Beclin1 (used for quantitation-only)_Short expos & Labeled.tif]

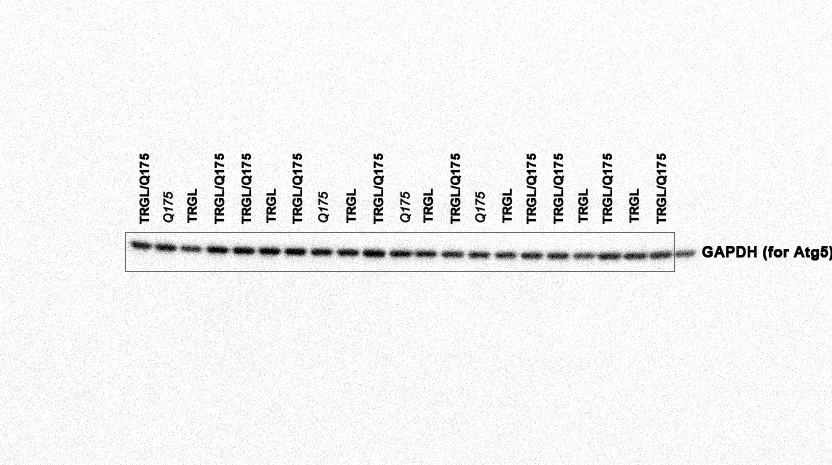

Supplement: Figure 3—figure supplement 2—source data 2. [file elife-104979-fig3-figsupp2-data2.zip › Figure 3-Figure supplement 2-Source Data 2/Beclin 1 & Atg5 only/Anti-GAPDH (for Atg5 used for quantitation-only)_Short expos & Labeled.tif]

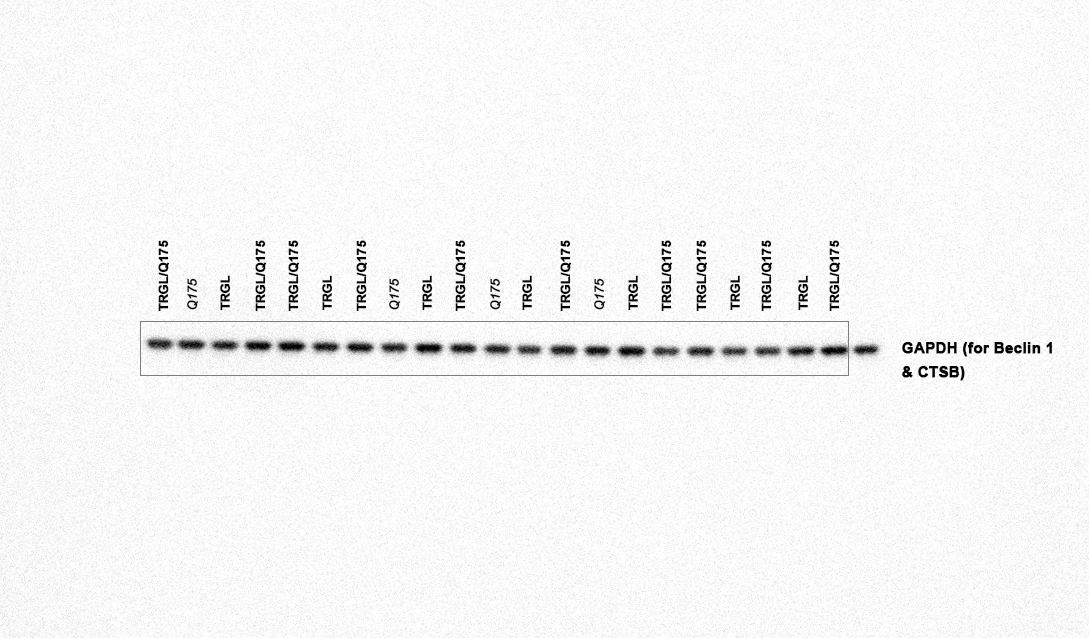

Supplement: Figure 3—figure supplement 2—source data 2. [file elife-104979-fig3-figsupp2-data2.zip › Figure 3-Figure supplement 2-Source Data 2/Beclin 1 & Atg5 only/Anti-GAPDH (for Beclin1 & CTSB used for quantitation-only)_Short expos & Labeled.tif]

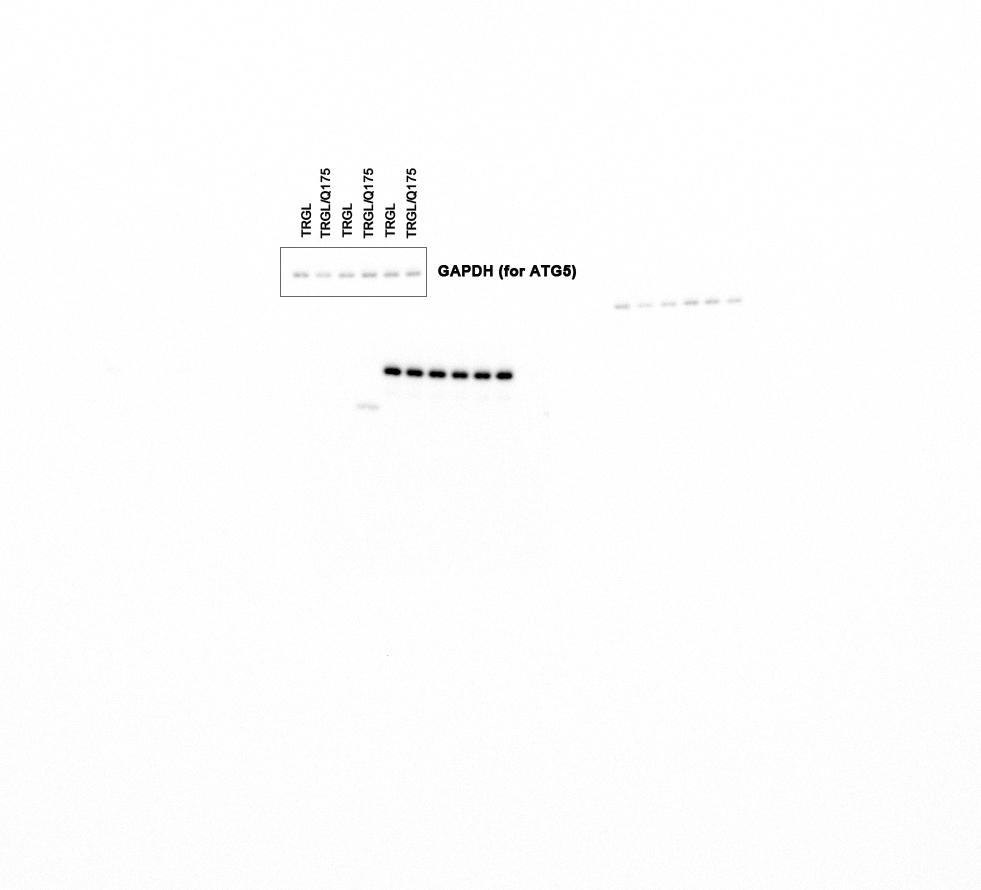

Supplement: Figure 3—figure supplement 2—source data 2. [file elife-104979-fig3-figsupp2-data2.zip › Figure 3-Figure supplement 2-Source Data 2/Beclin 1 & Atg5 only/Anti-GAPDH (mid-top, for ATG5 used for display-only)_Short expos & Labeled.tif]

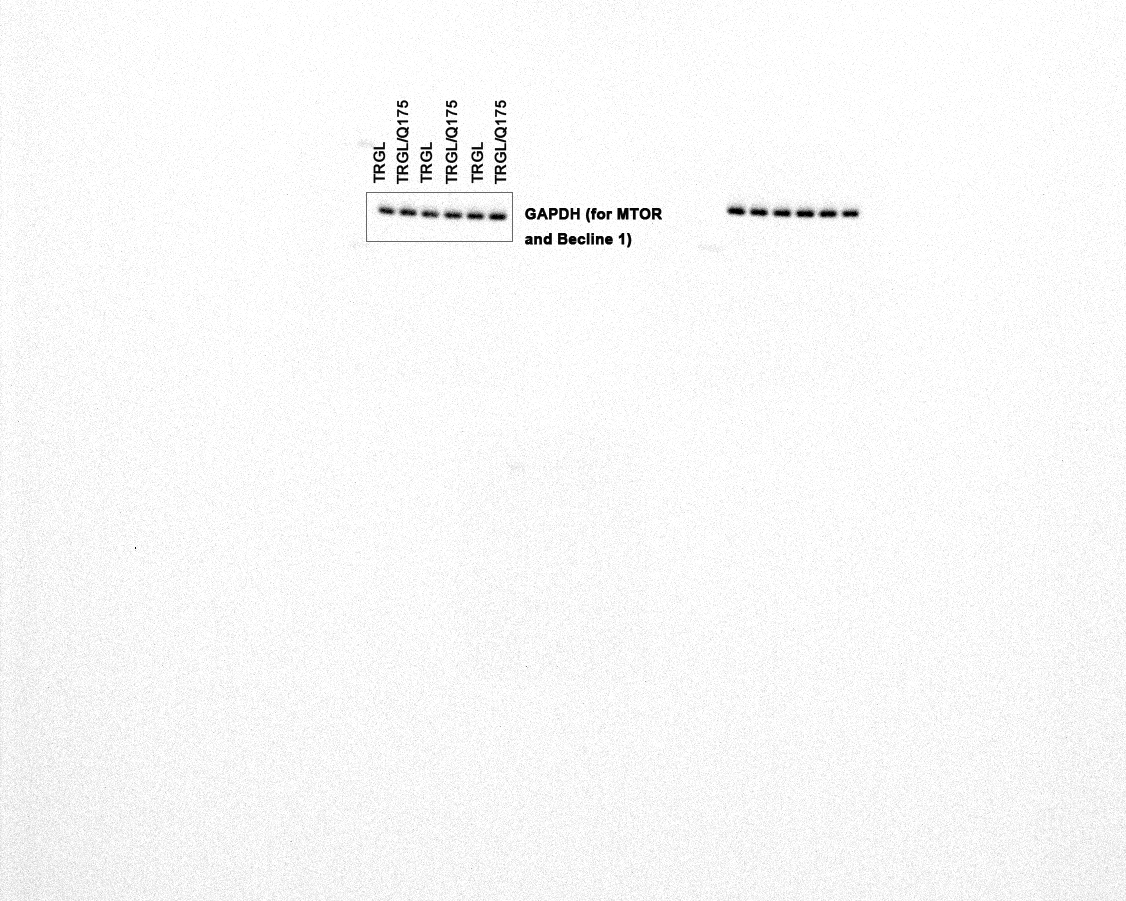

Supplement: Figure 3—figure supplement 2—source data 2. [file elife-104979-fig3-figsupp2-data2.zip › Figure 3-Figure supplement 2-Source Data 2/Beclin 1 & Atg5 only/Anti-GAPDH (upper left, for MTOR & Beclin1 used for display-only)_Short expos & Labeled.tif]

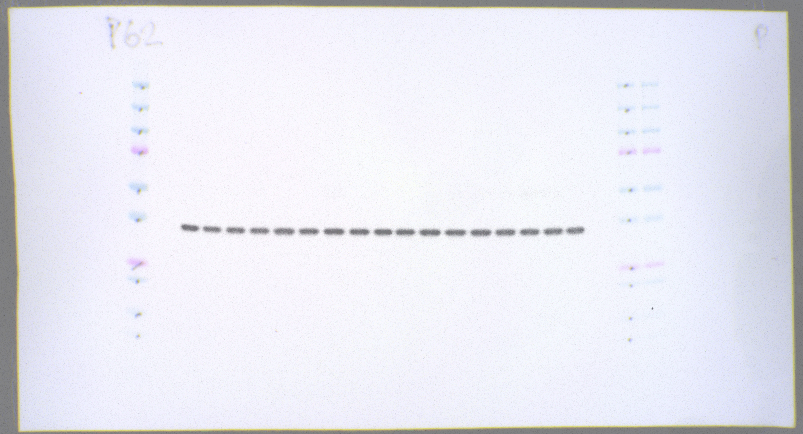

Supplement: Figure 3—figure supplement 3—source data 1. [file elife-104979-fig3-figsupp3-data1.zip › Figure 3-Figure supplement 3-Source Data 1/Anti-GAPDH (for p62)_Overlayed.tif]

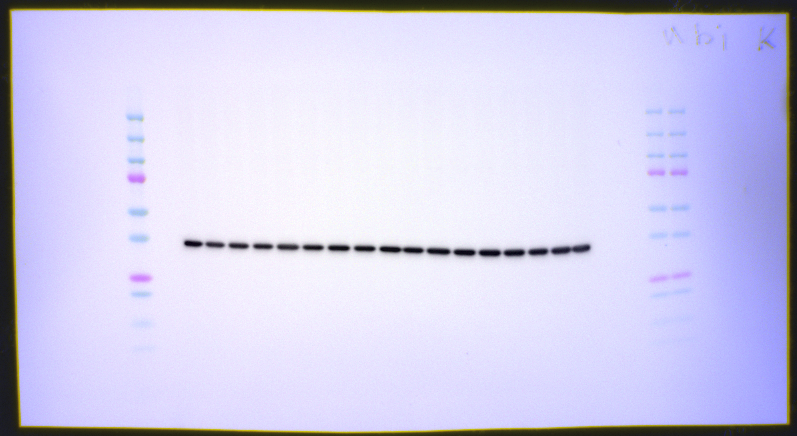

Supplement: Figure 3—figure supplement 3—source data 1. [file elife-104979-fig3-figsupp3-data1.zip › Figure 3-Figure supplement 3-Source Data 1/Anti-GAPDH (for Ubiquitin)_Overlayed.tif]

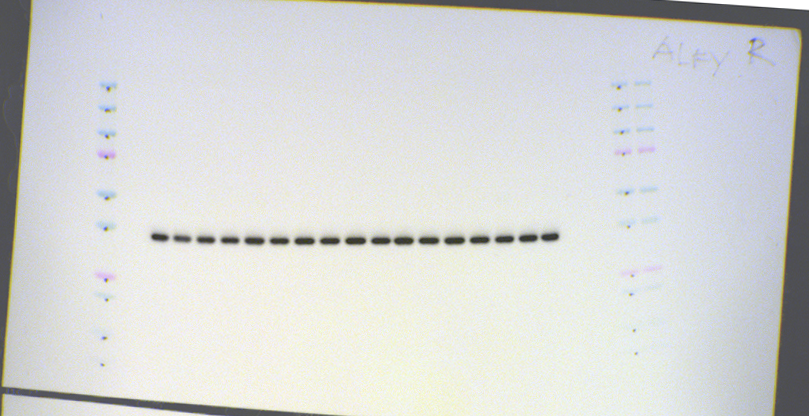

Supplement: Figure 3—figure supplement 3—source data 1. [file elife-104979-fig3-figsupp3-data1.zip › Figure 3-Figure supplement 3-Source Data 1/Anti-GAPDH (for WDFY3)_Overlayed.tif]

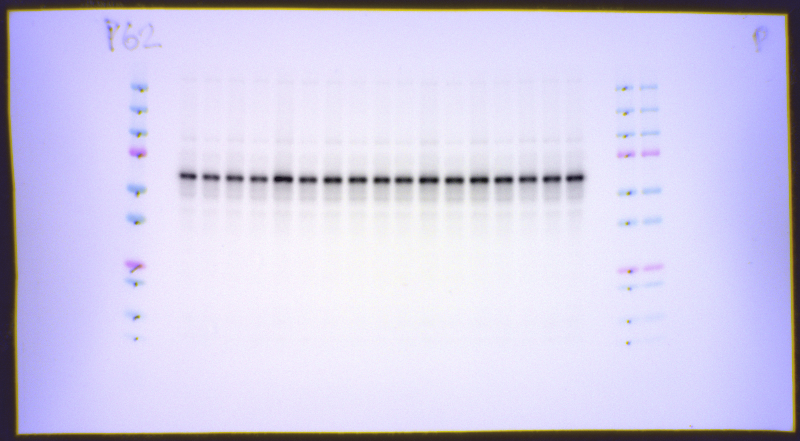

Supplement: Figure 3—figure supplement 3—source data 1. [file elife-104979-fig3-figsupp3-data1.zip › Figure 3-Figure supplement 3-Source Data 1/Anti-p62_Overlayed.tif]

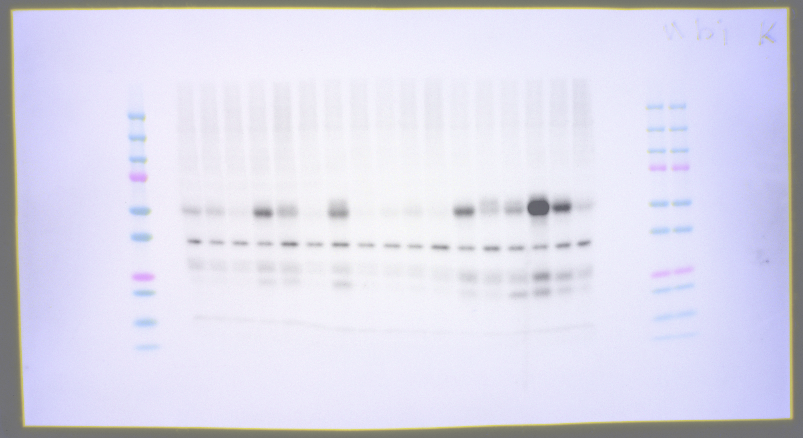

Supplement: Figure 3—figure supplement 3—source data 1. [file elife-104979-fig3-figsupp3-data1.zip › Figure 3-Figure supplement 3-Source Data 1/Anti-Ubiquitin_Overlayed.tif]

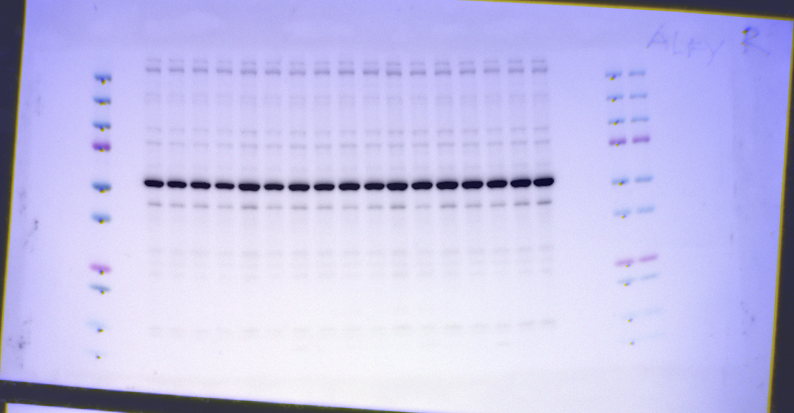

Supplement: Figure 3—figure supplement 3—source data 1. [file elife-104979-fig3-figsupp3-data1.zip › Figure 3-Figure supplement 3-Source Data 1/Anti-WDFY3 (Alfy)_Overlayed.tif]

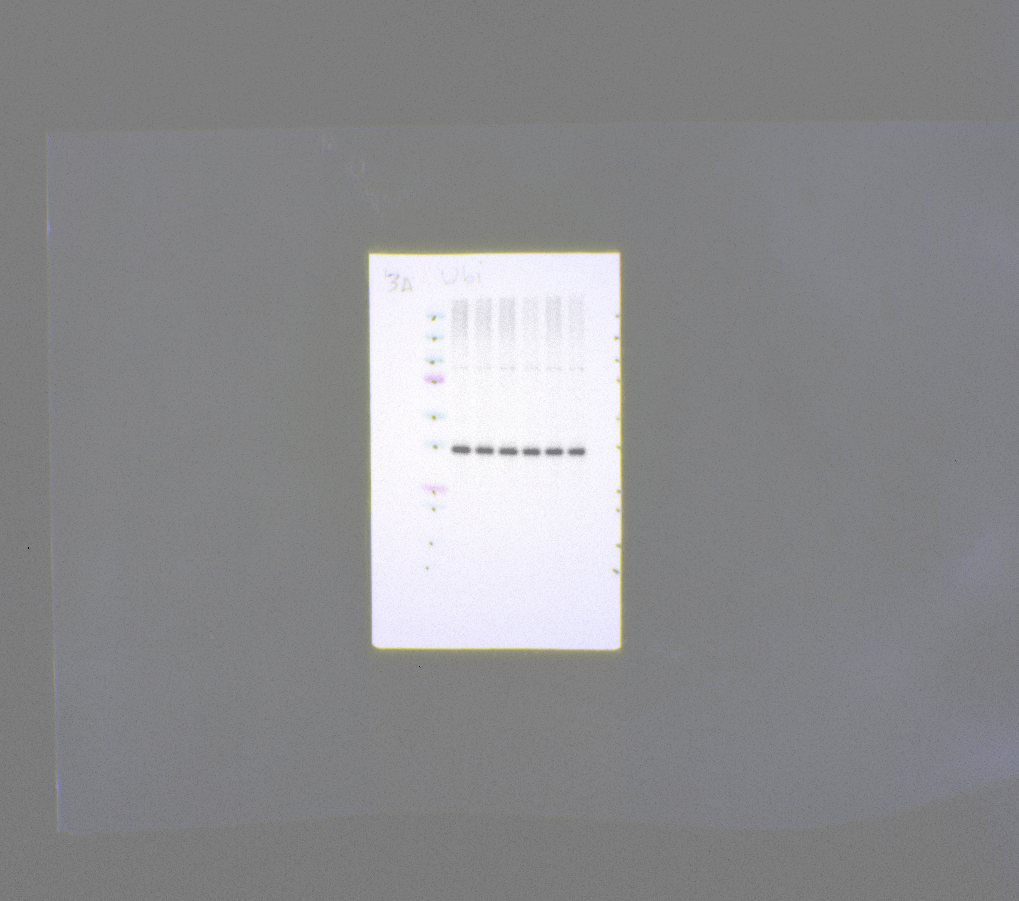

Supplement: Figure 3—figure supplement 3—source data 1. [file elife-104979-fig3-figsupp3-data1.zip › Figure 3-Figure supplement 3-Source Data 1/TRAF6 only/Anti-GAPDH (for TRAF6 used for display-only)_Overlayed.tif]

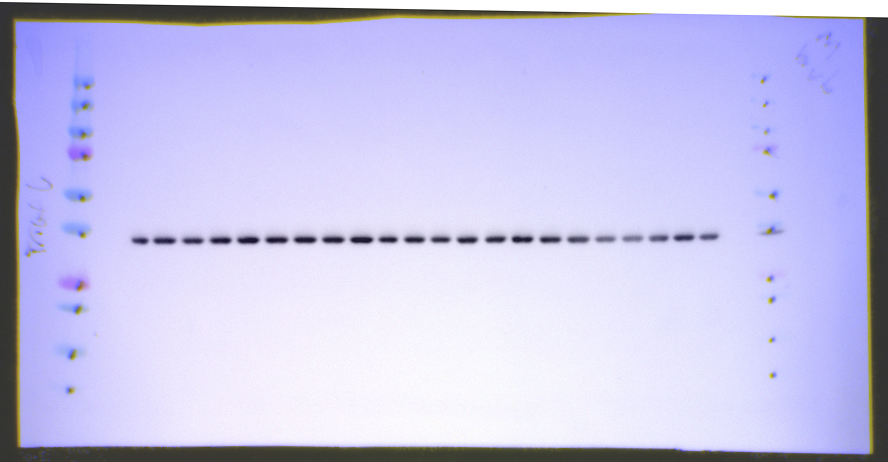

Supplement: Figure 3—figure supplement 3—source data 1. [file elife-104979-fig3-figsupp3-data1.zip › Figure 3-Figure supplement 3-Source Data 1/TRAF6 only/Anti-GAPDH (for TRAF6 used for quantitation-only)_Overlayed.tif]

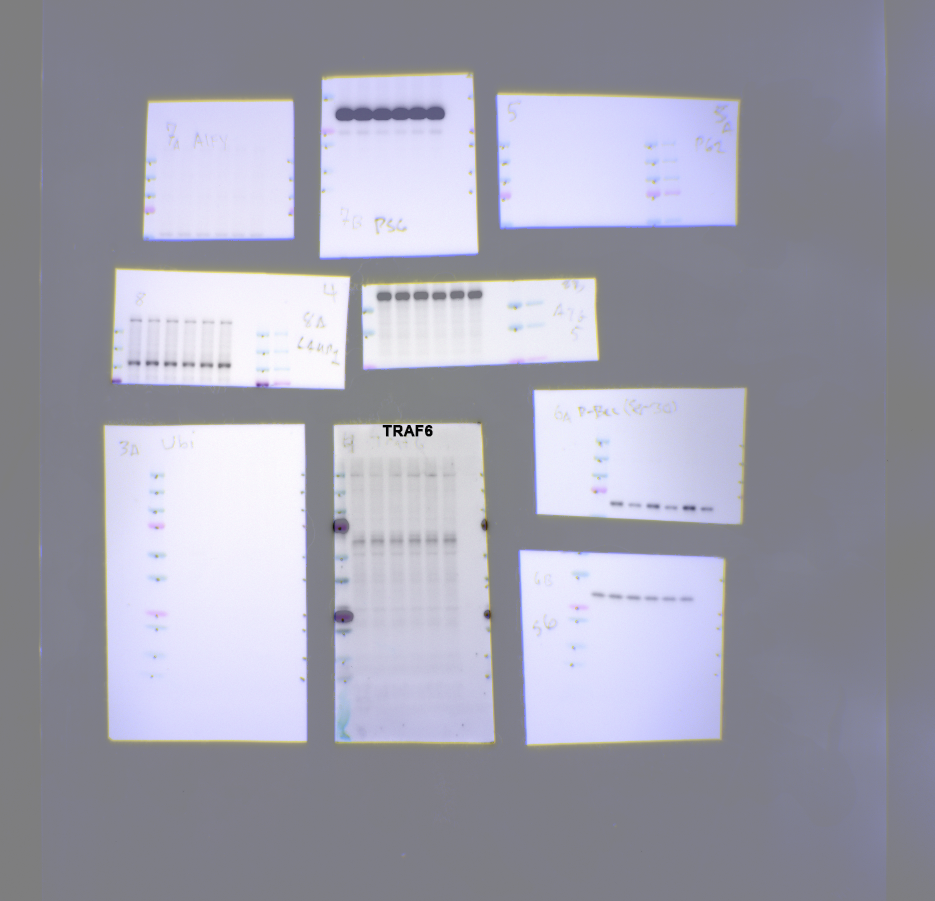

Supplement: Figure 3—figure supplement 3—source data 1. [file elife-104979-fig3-figsupp3-data1.zip › Figure 3-Figure supplement 3-Source Data 1/TRAF6 only/Anti-TRAF6 (bott-mid, used for display-only)_Overlayed.tif]

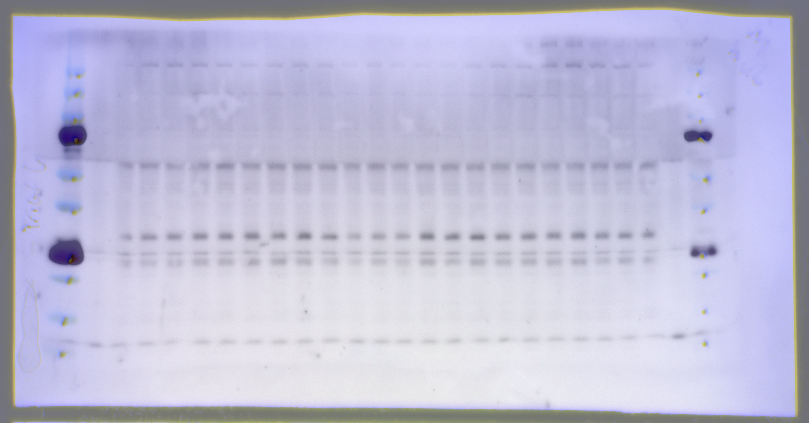

Supplement: Figure 3—figure supplement 3—source data 1. [file elife-104979-fig3-figsupp3-data1.zip › Figure 3-Figure supplement 3-Source Data 1/TRAF6 only/Anti-TRAF6 (used for quantitation-only)_Overlayed.tif]

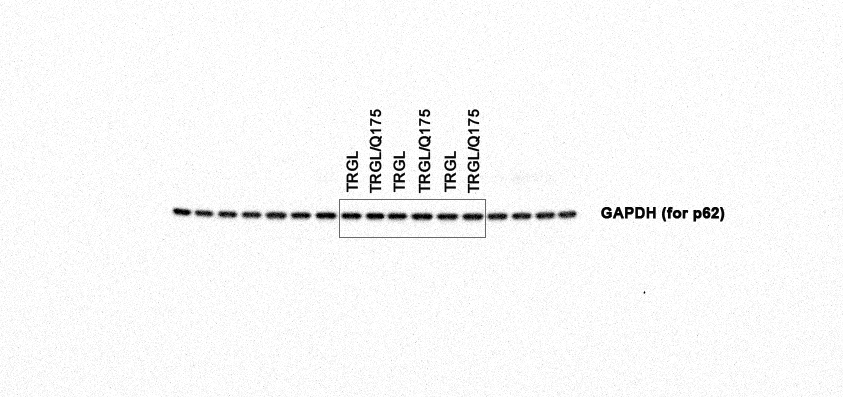

Supplement: Figure 3—figure supplement 3—source data 2. [file elife-104979-fig3-figsupp3-data2.zip › Figure 3-Figure supplement 3-Source Data 2/Anti-GAPDH (for p62)_Short expos & Labeled.tif]

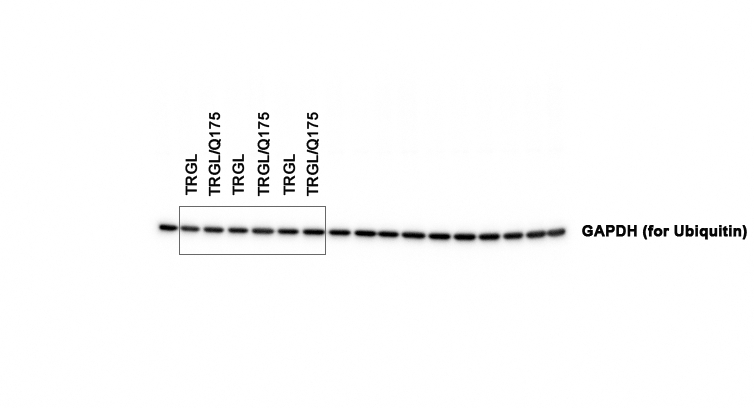

Supplement: Figure 3—figure supplement 3—source data 2. [file elife-104979-fig3-figsupp3-data2.zip › Figure 3-Figure supplement 3-Source Data 2/Anti-GAPDH (for Ubiquitin)_Short expos & Labeled.tif]

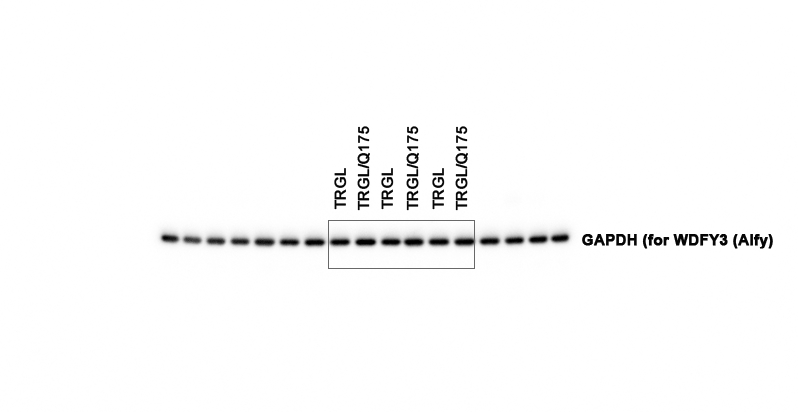

Supplement: Figure 3—figure supplement 3—source data 2. [file elife-104979-fig3-figsupp3-data2.zip › Figure 3-Figure supplement 3-Source Data 2/Anti-GAPDH (for WDFY3)_Short expos & Labeled.tif]

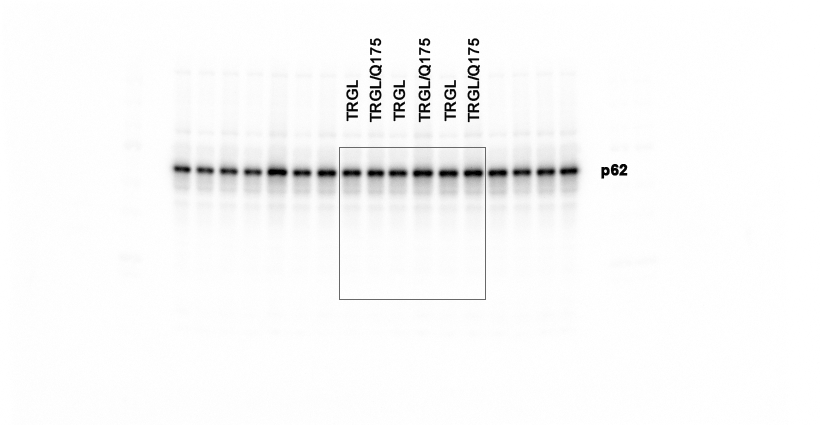

Supplement: Figure 3—figure supplement 3—source data 2. [file elife-104979-fig3-figsupp3-data2.zip › Figure 3-Figure supplement 3-Source Data 2/Anti-p62_Short expos & Labeled.tif]

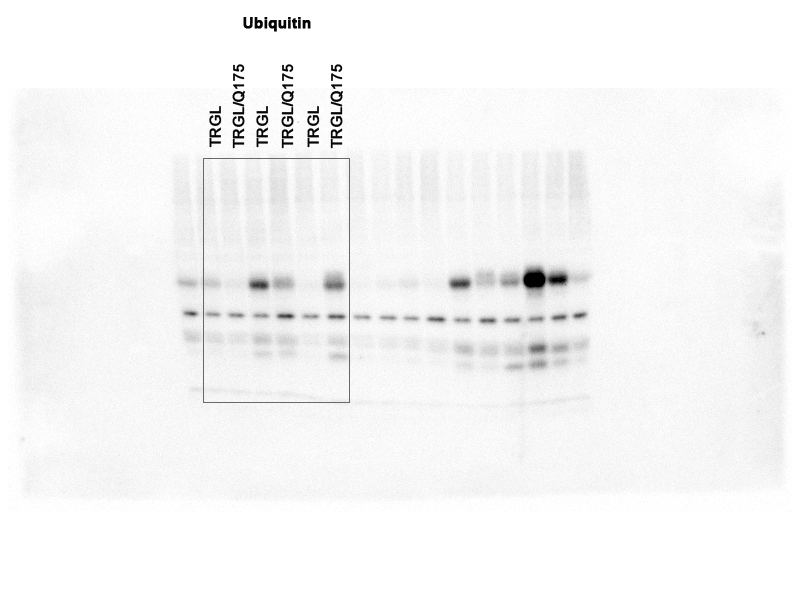

Supplement: Figure 3—figure supplement 3—source data 2. [file elife-104979-fig3-figsupp3-data2.zip › Figure 3-Figure supplement 3-Source Data 2/Anti-Ubiquitin_Short expos & Labeled.tif]

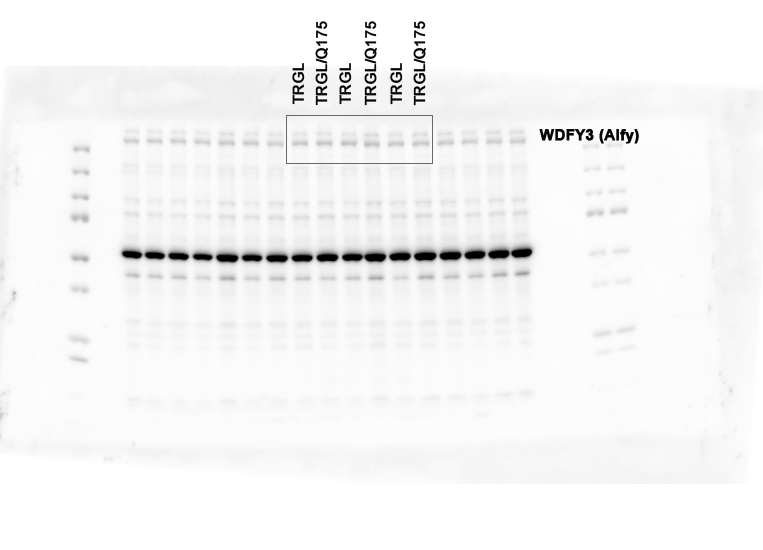

Supplement: Figure 3—figure supplement 3—source data 2. [file elife-104979-fig3-figsupp3-data2.zip › Figure 3-Figure supplement 3-Source Data 2/Anti-WDFY3 (Alfy)_Short expos & Labeled.tif]

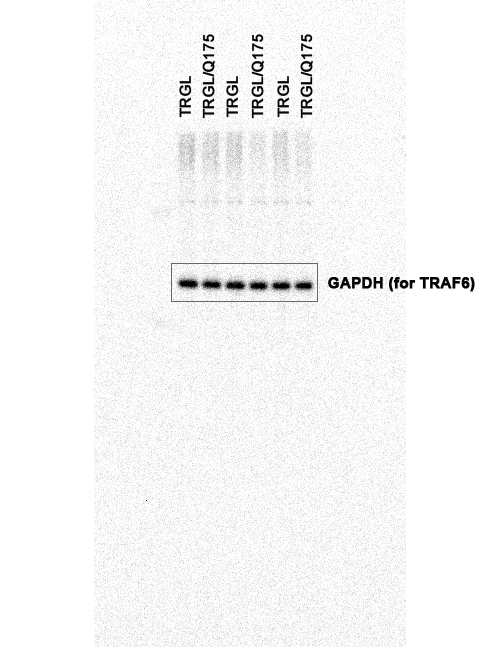

Supplement: Figure 3—figure supplement 3—source data 2. [file elife-104979-fig3-figsupp3-data2.zip › Figure 3-Figure supplement 3-Source Data 2/TRAF6 only/Anti-GAPDH (for TRAF6 used for display-only)_Short expos & Labeled.tif]

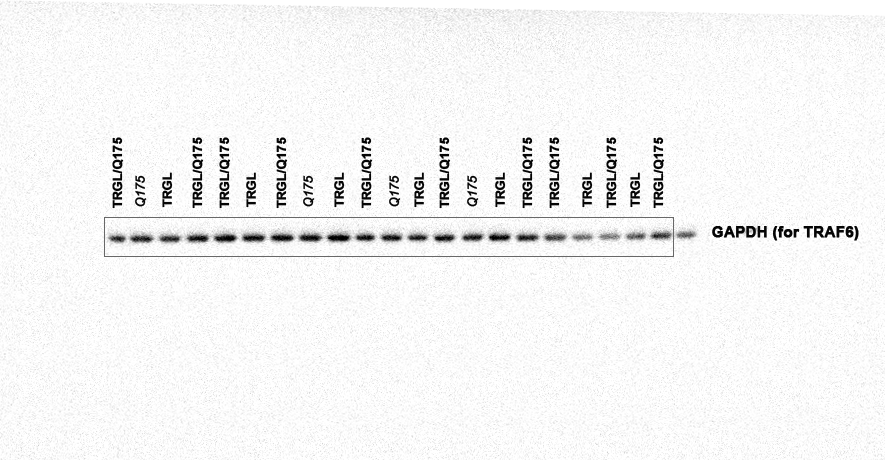

Supplement: Figure 3—figure supplement 3—source data 2. [file elife-104979-fig3-figsupp3-data2.zip › Figure 3-Figure supplement 3-Source Data 2/TRAF6 only/Anti-GAPDH (for TRAF6 used for quantitation-only)_Short expos & Labeled.tif]

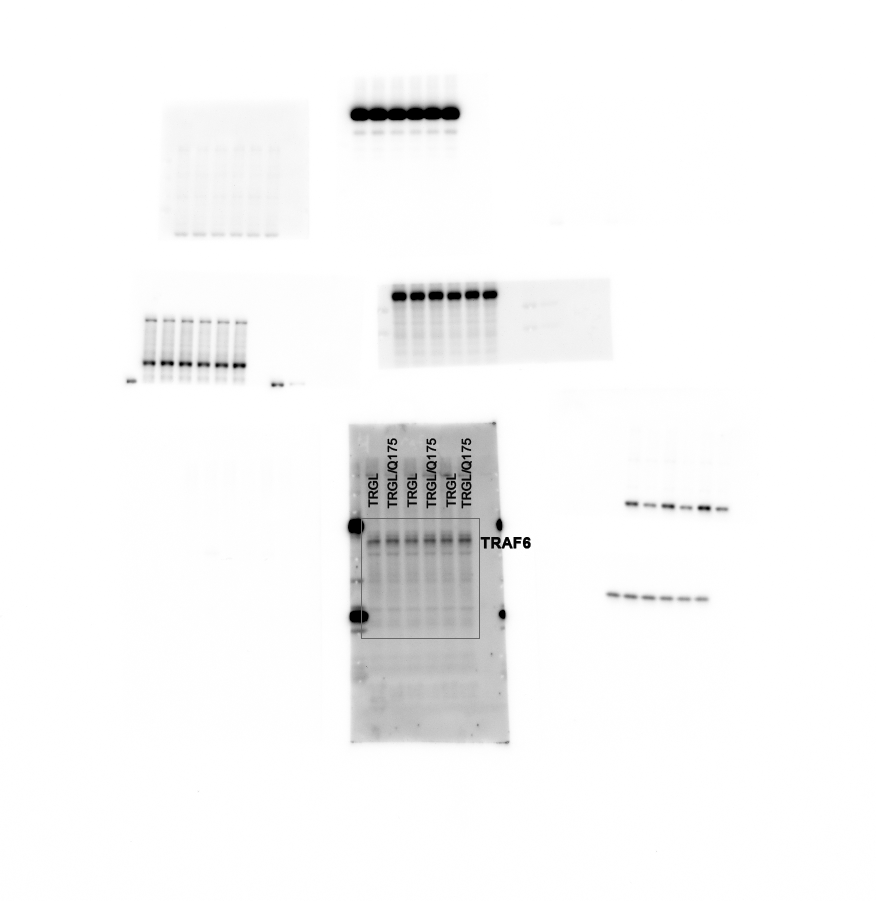

Supplement: Figure 3—figure supplement 3—source data 2. [file elife-104979-fig3-figsupp3-data2.zip › Figure 3-Figure supplement 3-Source Data 2/TRAF6 only/Anti-TRAF6 (bott-mid, used for display-only)_Short expos & Labeled.tif]

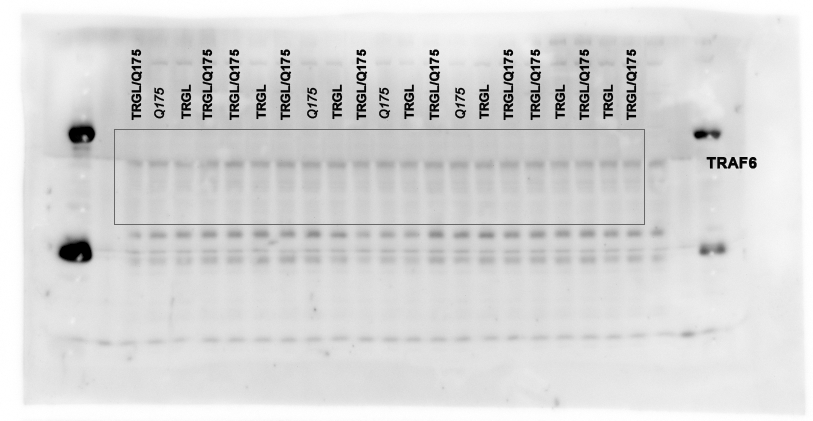

Supplement: Figure 3—figure supplement 3—source data 2. [file elife-104979-fig3-figsupp3-data2.zip › Figure 3-Figure supplement 3-Source Data 2/TRAF6 only/Anti-TRAF6 (used for quantitation-only)_Short expos & Labeled.tif]

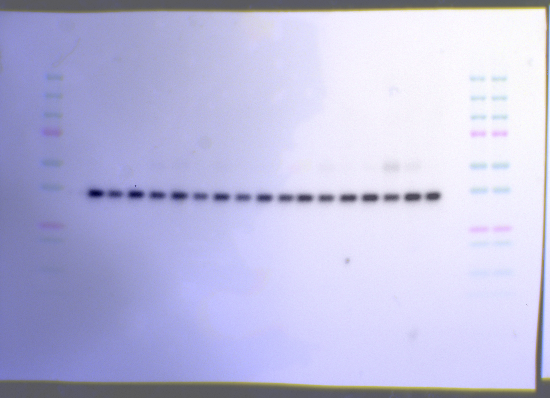

Supplement: Figure 3—figure supplement 4—source data 1. [file elife-104979-fig3-figsupp4-data1.zip › Figure 3-Figure supplement 4-Source Data 1/Anti-GAPDH (for LAMP1)_Overlayed.tif]

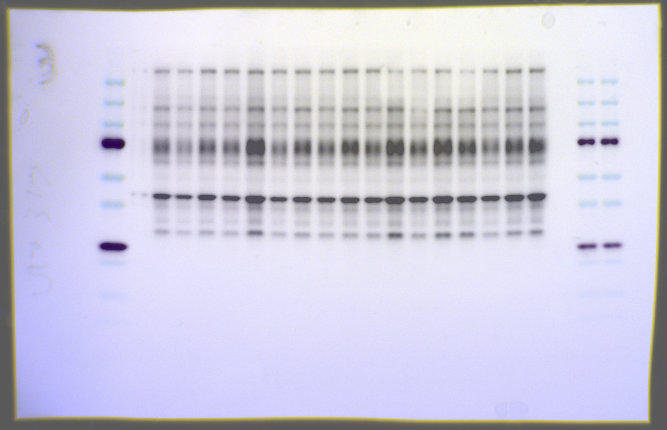

Supplement: Figure 3—figure supplement 4—source data 1. [file elife-104979-fig3-figsupp4-data1.zip › Figure 3-Figure supplement 4-Source Data 1/Anti-LAMP1_Overlayed.tif]

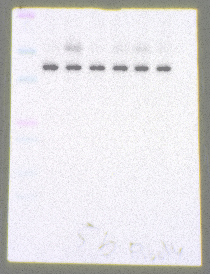

Supplement: Figure 3—figure supplement 4—source data 1. [file elife-104979-fig3-figsupp4-data1.zip › Figure 3-Figure supplement 4-Source Data 1/CTSB & CTSD/Anti-Actin (for CTSD used for display-only)_Overlayed.tif]

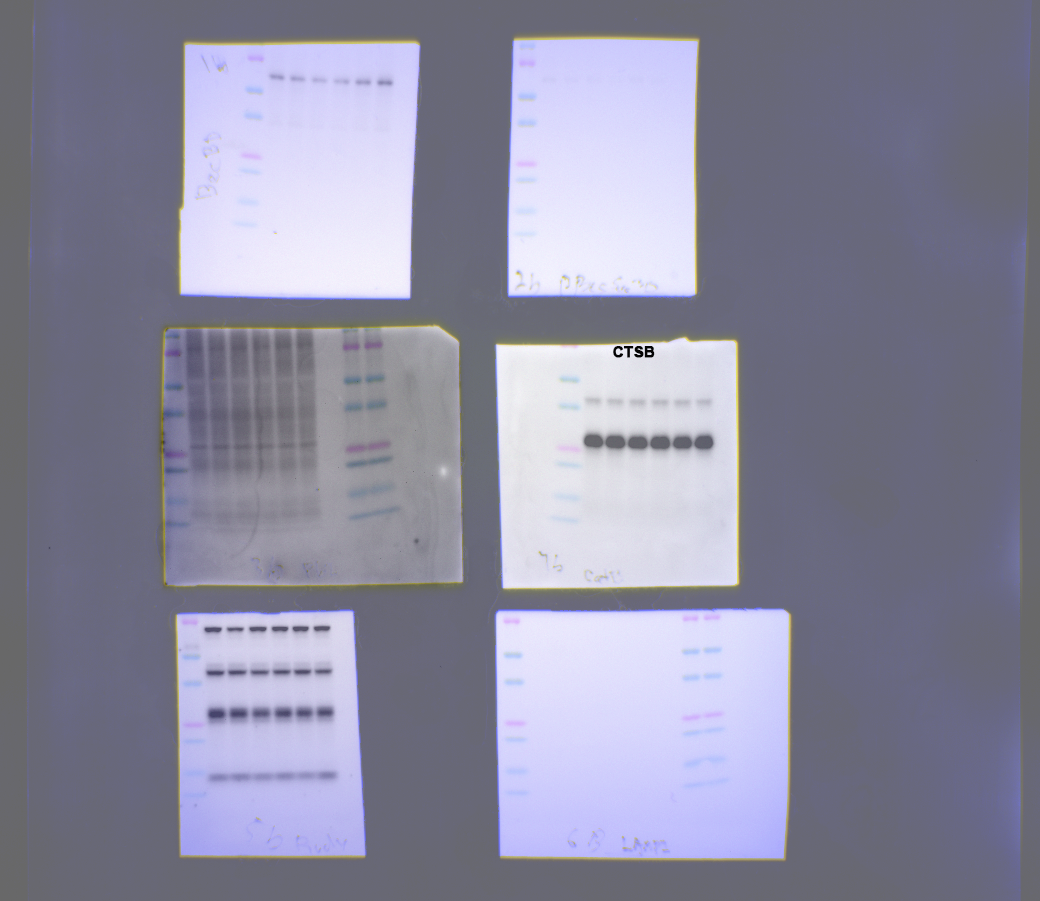

Supplement: Figure 3—figure supplement 4—source data 1. [file elife-104979-fig3-figsupp4-data1.zip › Figure 3-Figure supplement 4-Source Data 1/CTSB & CTSD/Anti-CTSB (mid-right, used for display-only)_Overlayed.tif]

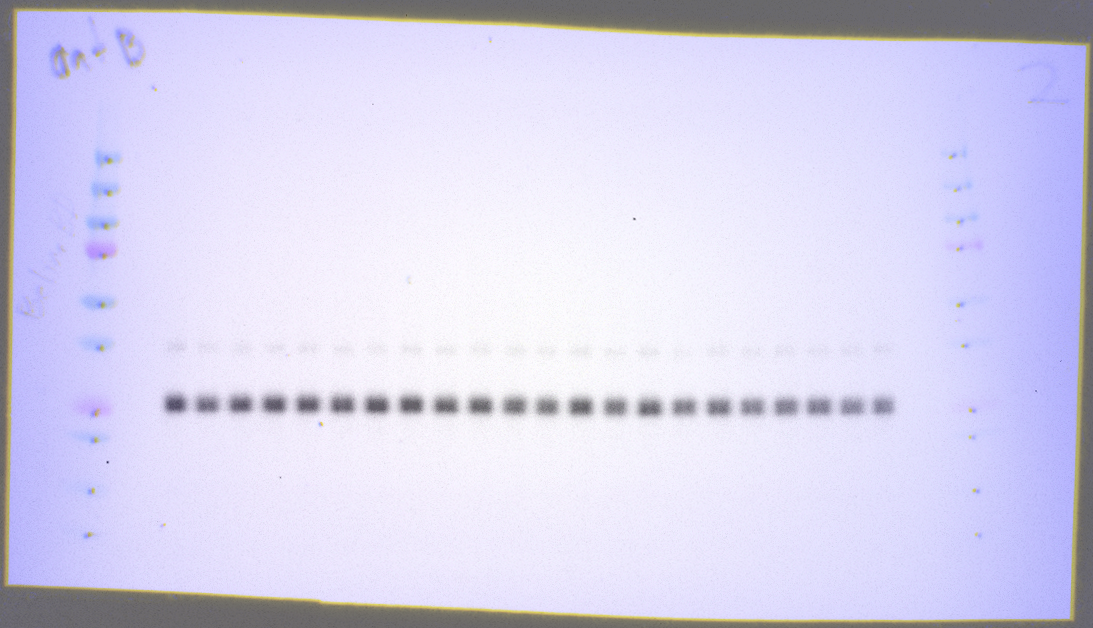

Supplement: Figure 3—figure supplement 4—source data 1. [file elife-104979-fig3-figsupp4-data1.zip › Figure 3-Figure supplement 4-Source Data 1/CTSB & CTSD/Anti-CTSB (used for quantitation-only)_Overlayed.tif]

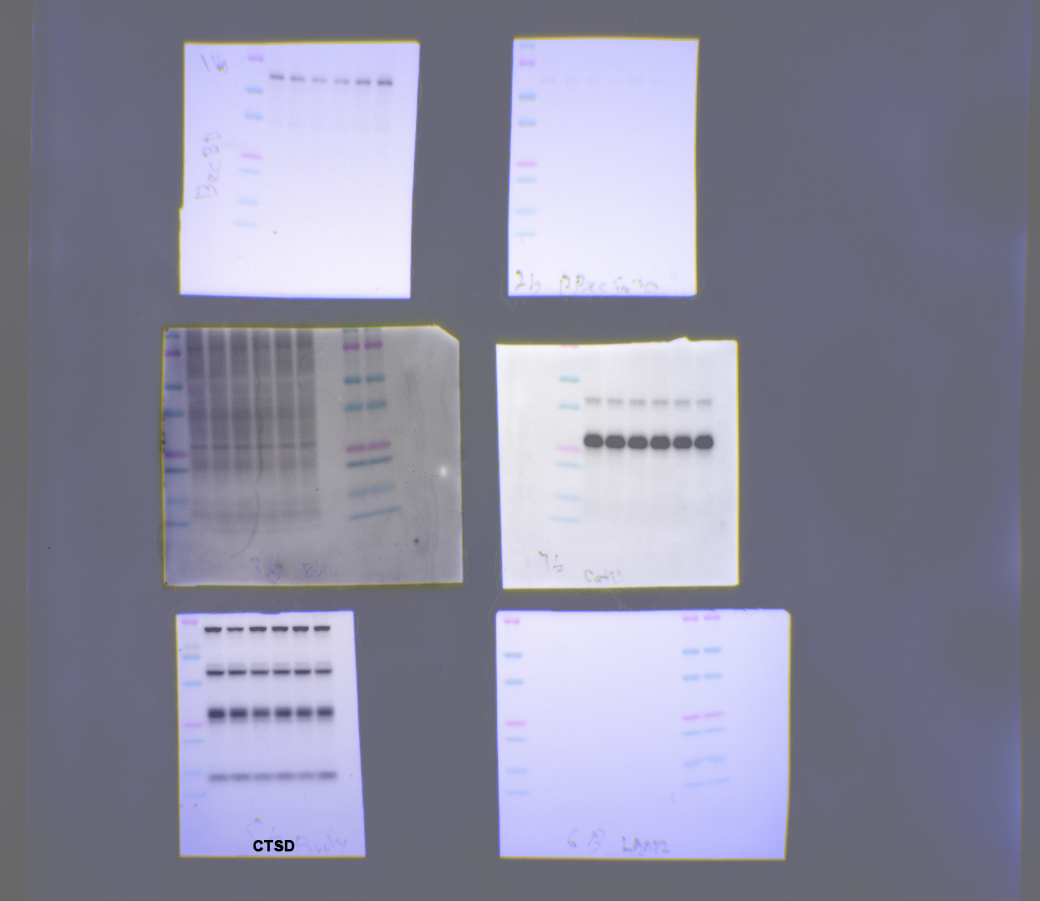

Supplement: Figure 3—figure supplement 4—source data 1. [file elife-104979-fig3-figsupp4-data1.zip › Figure 3-Figure supplement 4-Source Data 1/CTSB & CTSD/Anti-CTSD (bot-left, used for display-only)_Overlayed.tif]

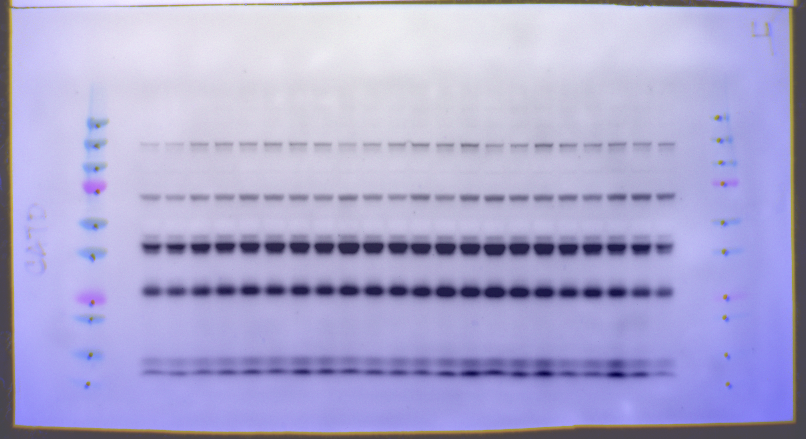

Supplement: Figure 3—figure supplement 4—source data 1. [file elife-104979-fig3-figsupp4-data1.zip › Figure 3-Figure supplement 4-Source Data 1/CTSB & CTSD/Anti-CTSD (used for quantitation-only)_Overlayed.tif]

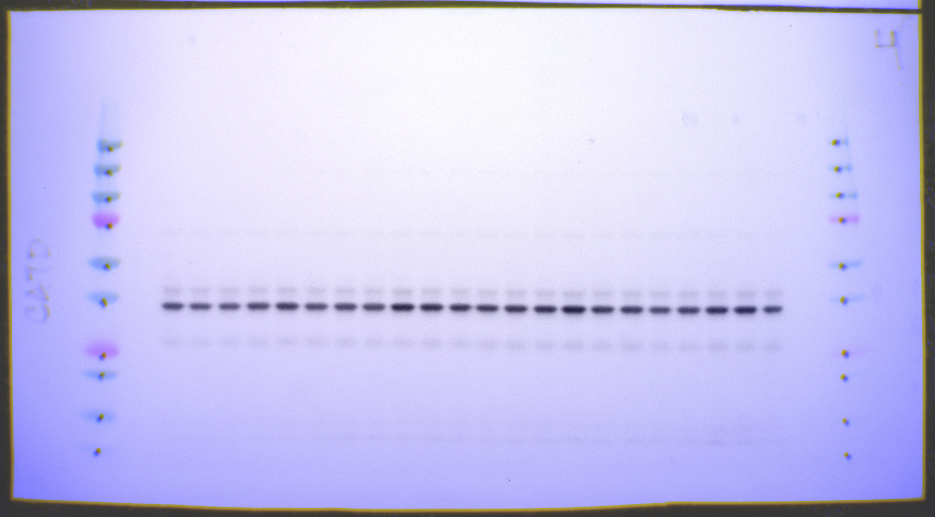

Supplement: Figure 3—figure supplement 4—source data 1. [file elife-104979-fig3-figsupp4-data1.zip › Figure 3-Figure supplement 4-Source Data 1/CTSB & CTSD/Anti-GAPDH (for CTSD used for quantitation-only)_Overlayed.tif]

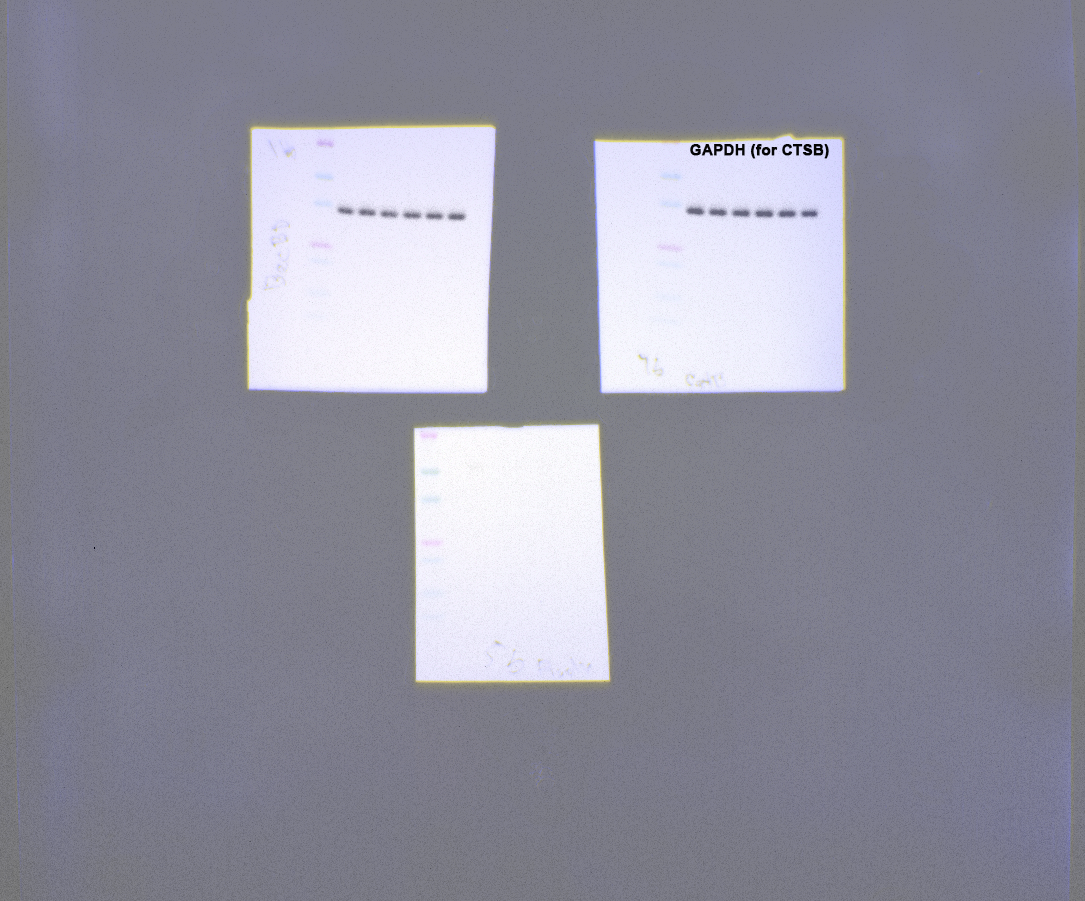

Supplement: Figure 3—figure supplement 4—source data 1. [file elife-104979-fig3-figsupp4-data1.zip › Figure 3-Figure supplement 4-Source Data 1/CTSB & CTSD/Anti-GAPDH (right, for CTSB used for display-only)_Overlayed.tif]

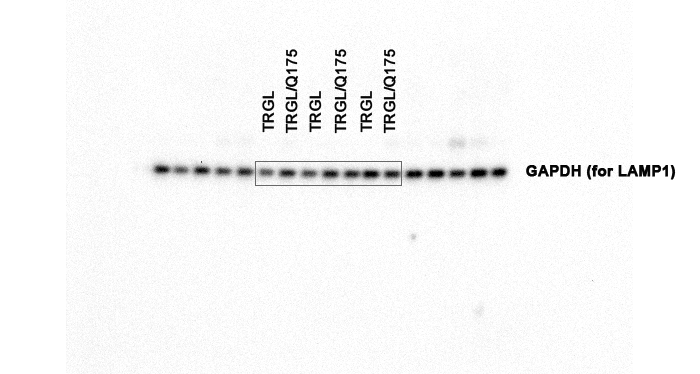

Supplement: Figure 3—figure supplement 4—source data 2. [file elife-104979-fig3-figsupp4-data2.zip › Figure 3-Figure supplement 4-Source Data 2/Anti-GAPDH (for LAMP1)_Short expos & Labeled.tif]

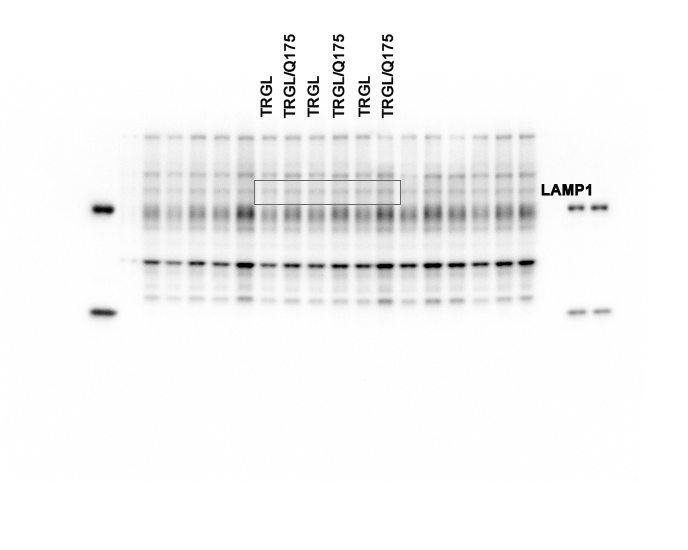

Supplement: Figure 3—figure supplement 4—source data 2. [file elife-104979-fig3-figsupp4-data2.zip › Figure 3-Figure supplement 4-Source Data 2/Anti-LAMP1_Short expos & Labeled.tif]

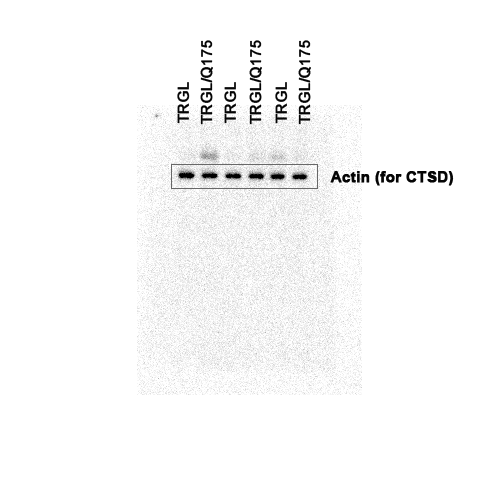

Supplement: Figure 3—figure supplement 4—source data 2. [file elife-104979-fig3-figsupp4-data2.zip › Figure 3-Figure supplement 4-Source Data 2/CTSB & CTSD/Anti-Actin (for CTSD used for display-only)_Short expos & Labeled.tif]

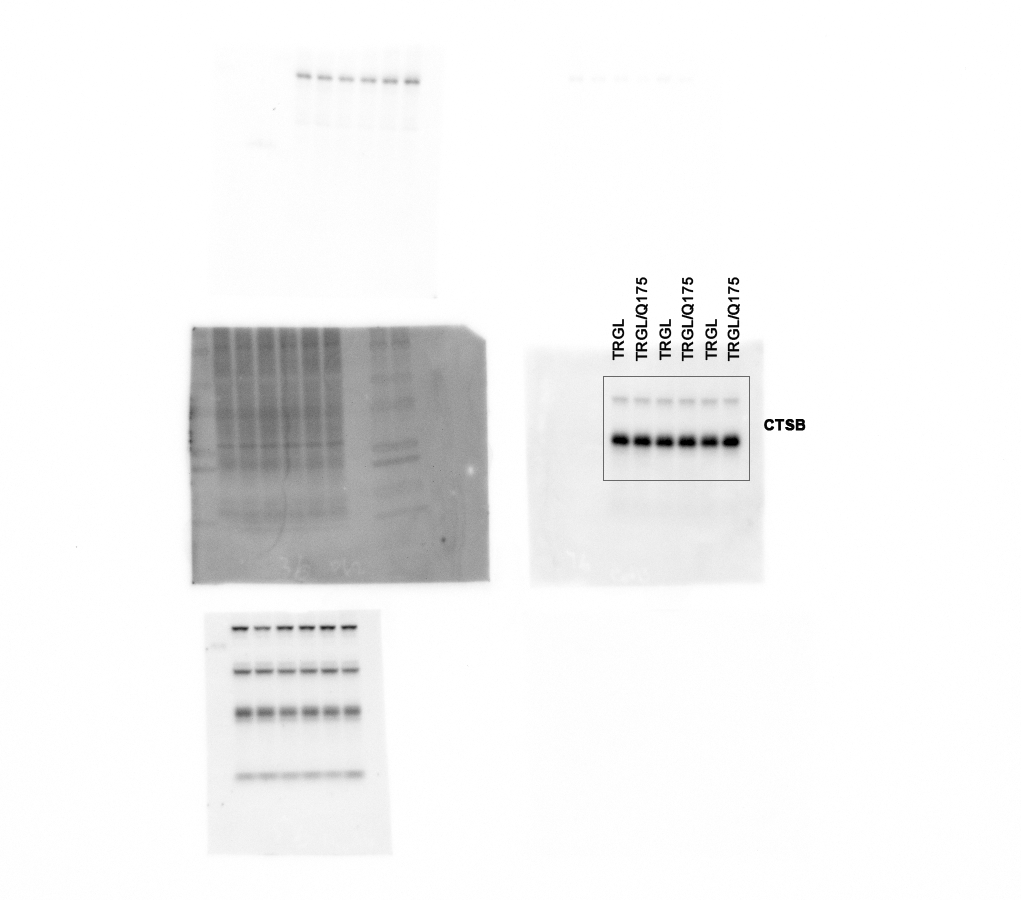

Supplement: Figure 3—figure supplement 4—source data 2. [file elife-104979-fig3-figsupp4-data2.zip › Figure 3-Figure supplement 4-Source Data 2/CTSB & CTSD/Anti-CTSB (mid-right, used for display-only)_Short expos & Labeled.tif]

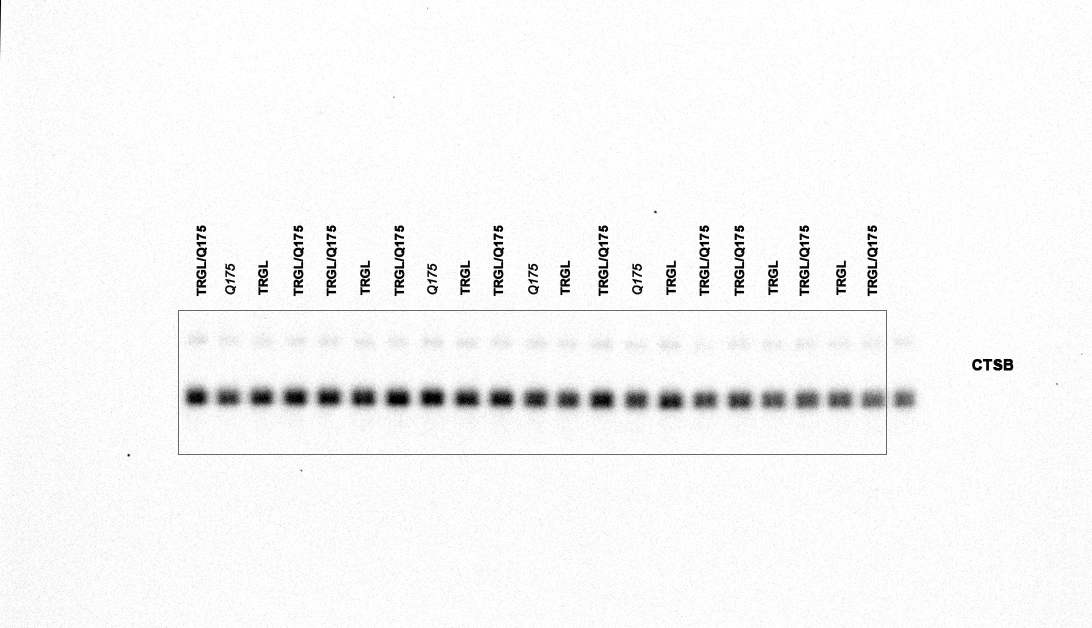

Supplement: Figure 3—figure supplement 4—source data 2. [file elife-104979-fig3-figsupp4-data2.zip › Figure 3-Figure supplement 4-Source Data 2/CTSB & CTSD/Anti-CTSB (used for quantitation-only)_Short expos & Labeled.tif]

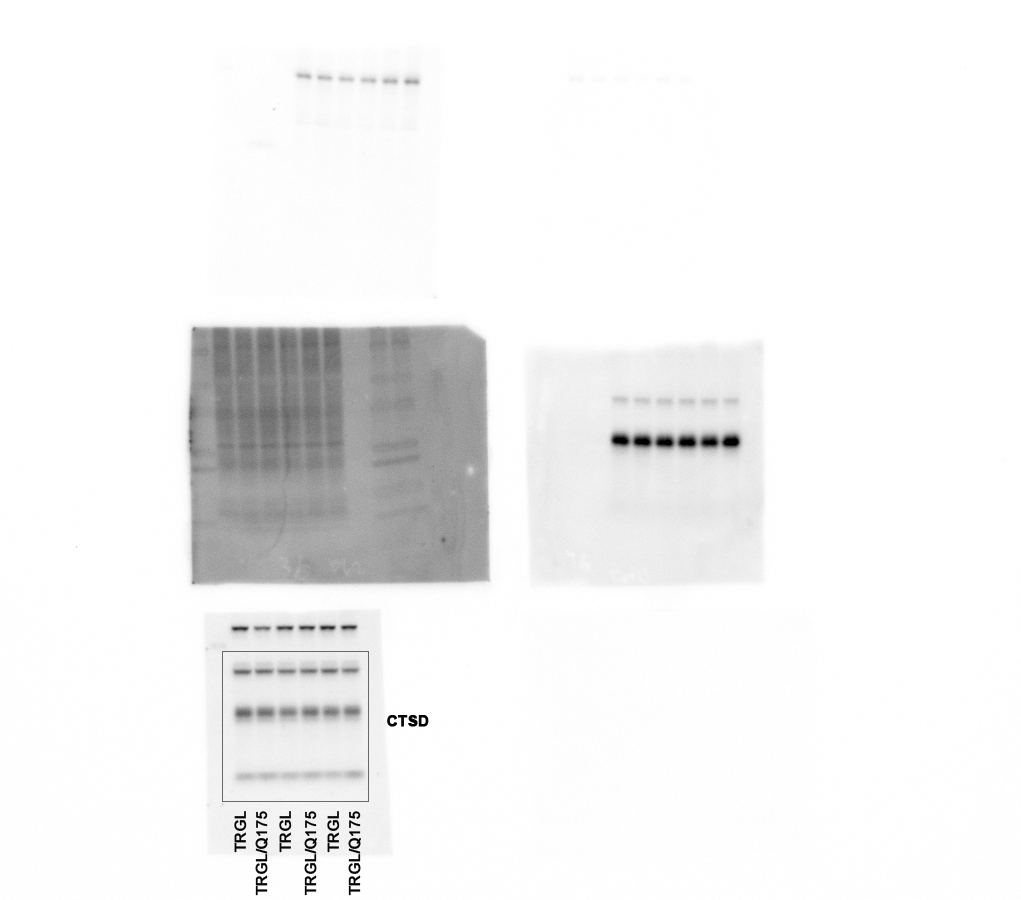

Supplement: Figure 3—figure supplement 4—source data 2. [file elife-104979-fig3-figsupp4-data2.zip › Figure 3-Figure supplement 4-Source Data 2/CTSB & CTSD/Anti-CTSD (bot-left, used for display-only)_Short expos & Labeled.tif]

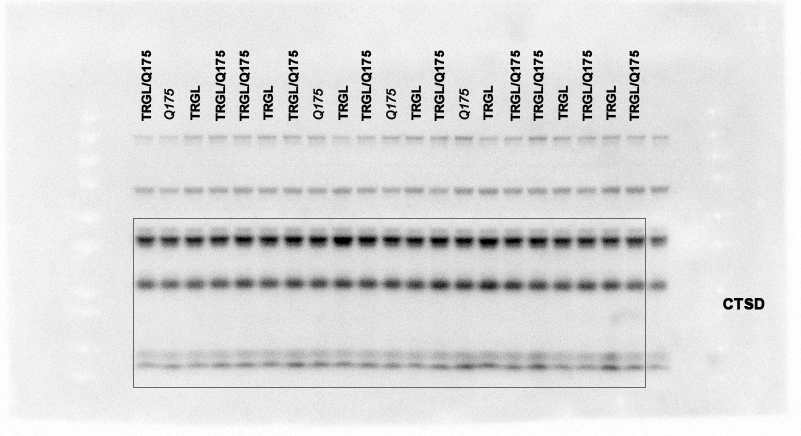

Supplement: Figure 3—figure supplement 4—source data 2. [file elife-104979-fig3-figsupp4-data2.zip › Figure 3-Figure supplement 4-Source Data 2/CTSB & CTSD/Anti-CTSD (used for quantitation-only)_Short expos & Labeled.tif]

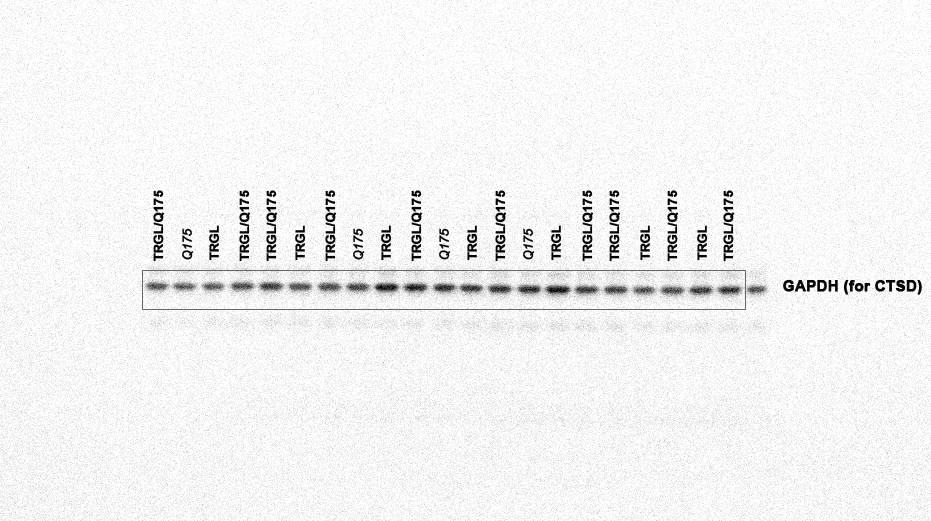

Supplement: Figure 3—figure supplement 4—source data 2. [file elife-104979-fig3-figsupp4-data2.zip › Figure 3-Figure supplement 4-Source Data 2/CTSB & CTSD/Anti-GAPDH (for CTSD used for quantitation-only)_Short expos & Labeled.tif]

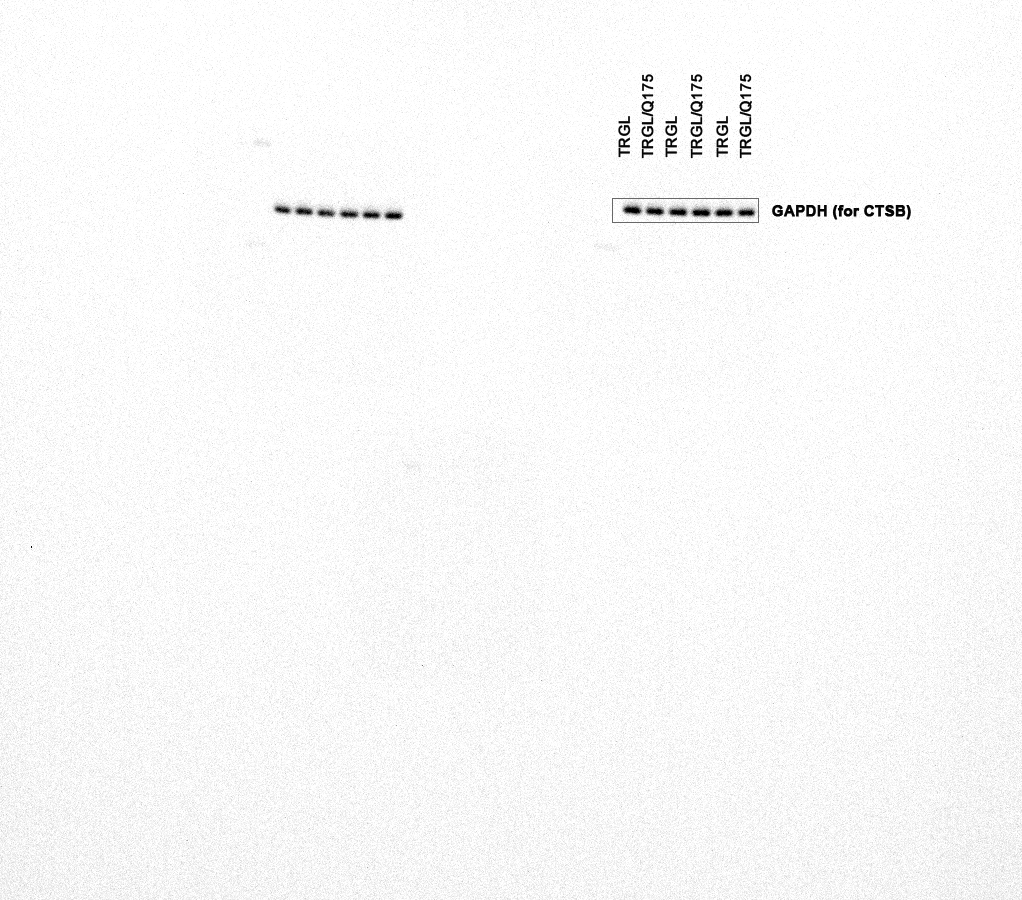

Supplement: Figure 3—figure supplement 4—source data 2. [file elife-104979-fig3-figsupp4-data2.zip › Figure 3-Figure supplement 4-Source Data 2/CTSB & CTSD/Anti-GAPDH (right, for CTSB used for display-only)_Short expos & Labeled.tif]

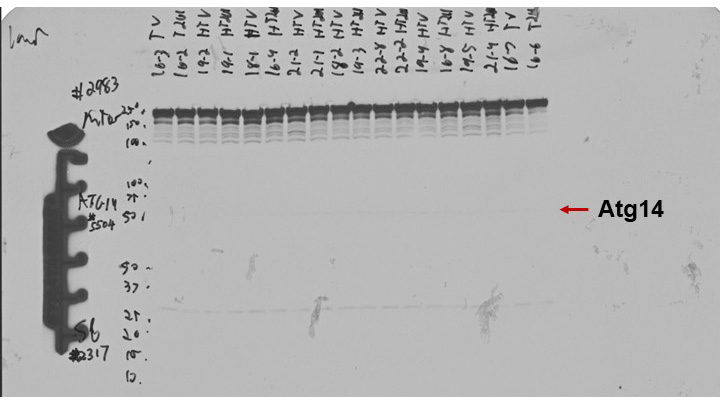

Supplement: Figure 5—source data 1. [file elife-104979-fig5-data1.zip › Figure 5A-Source Data 1/Fig 5A_Anti-Atg14 (middle)(too weak)_see long expos on the Labeled file.jpg]

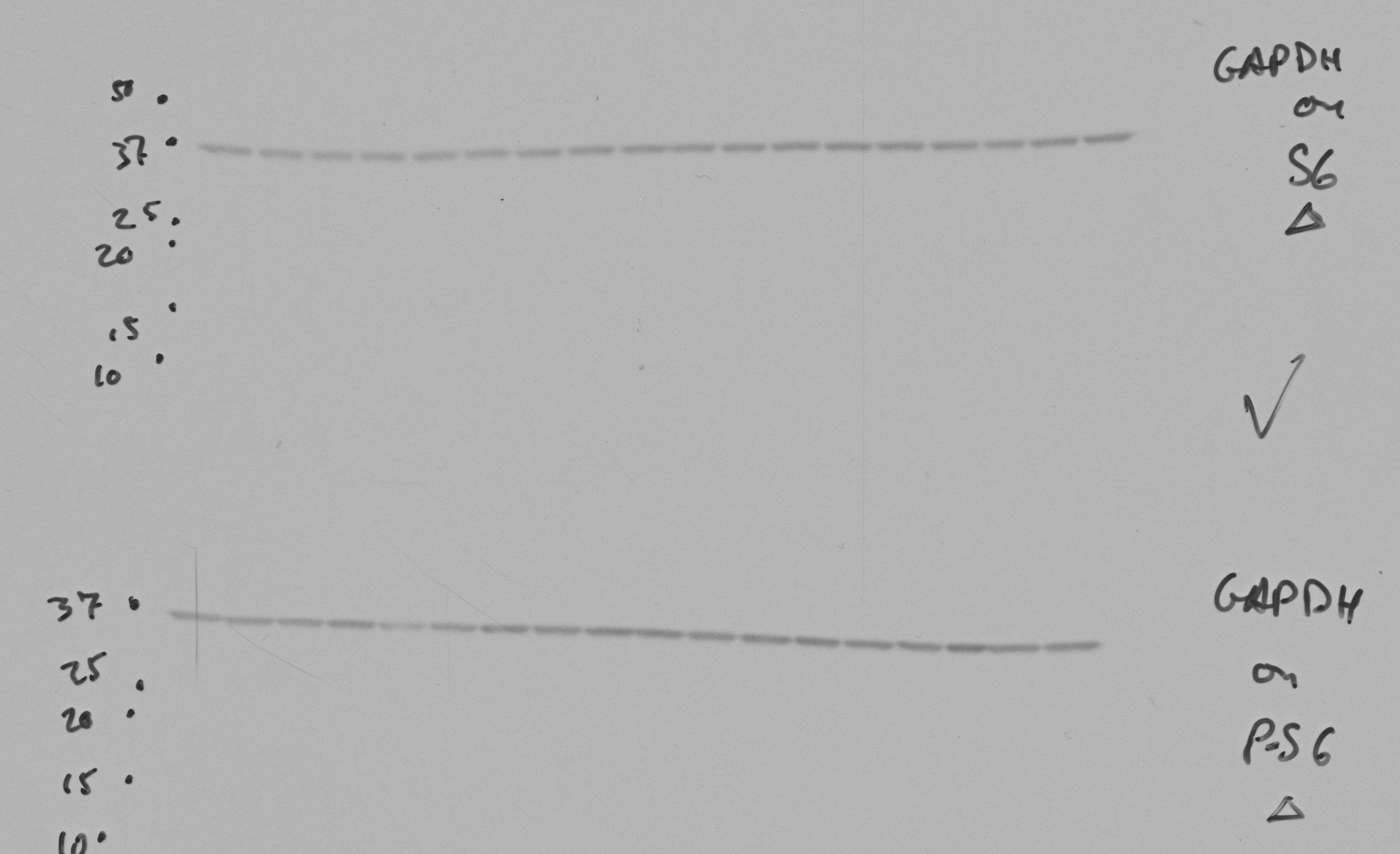

Supplement: Figure 5—source data 1. [file elife-104979-fig5-data1.zip › Figure 5A-Source Data 1/Fig 5A_Anti-GAPDH)_top for Atg14 (& S6), bottom for p-Atg14 (& p-S6).jpg]

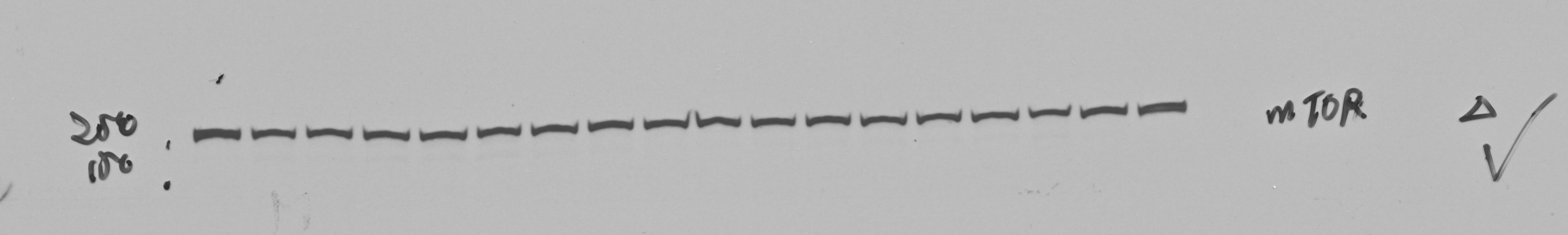

Supplement: Figure 5—source data 1. [file elife-104979-fig5-data1.zip › Figure 5A-Source Data 1/Fig 5A_Anti-mTOR.jpg]

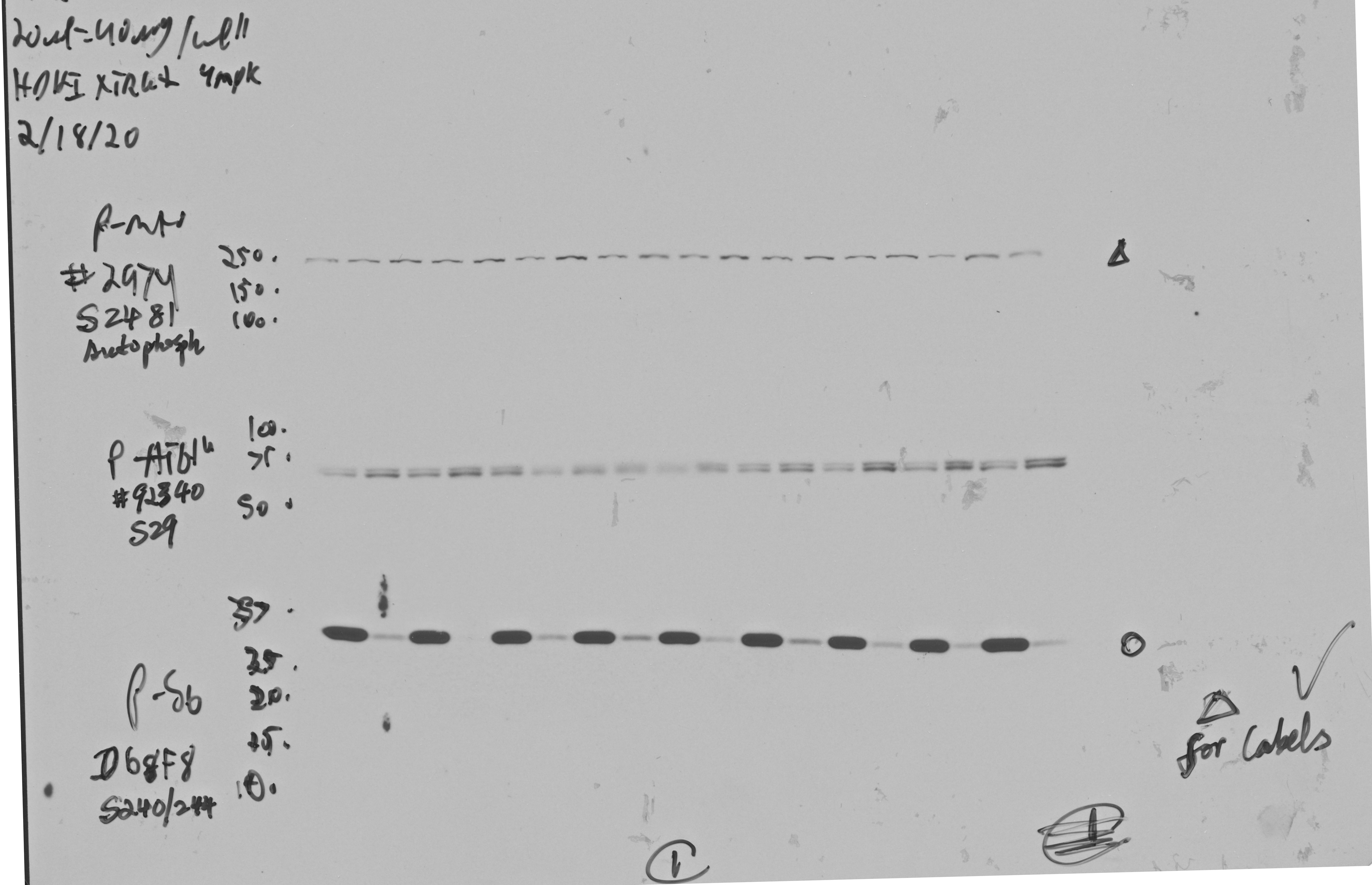

Supplement: Figure 5—source data 1. [file elife-104979-fig5-data1.zip › Figure 5A-Source Data 1/Fig 5A_Anti-p-mTOR (top), anti-p-Atg14 (middle) & anti-p-S6 (bottom).jpg]

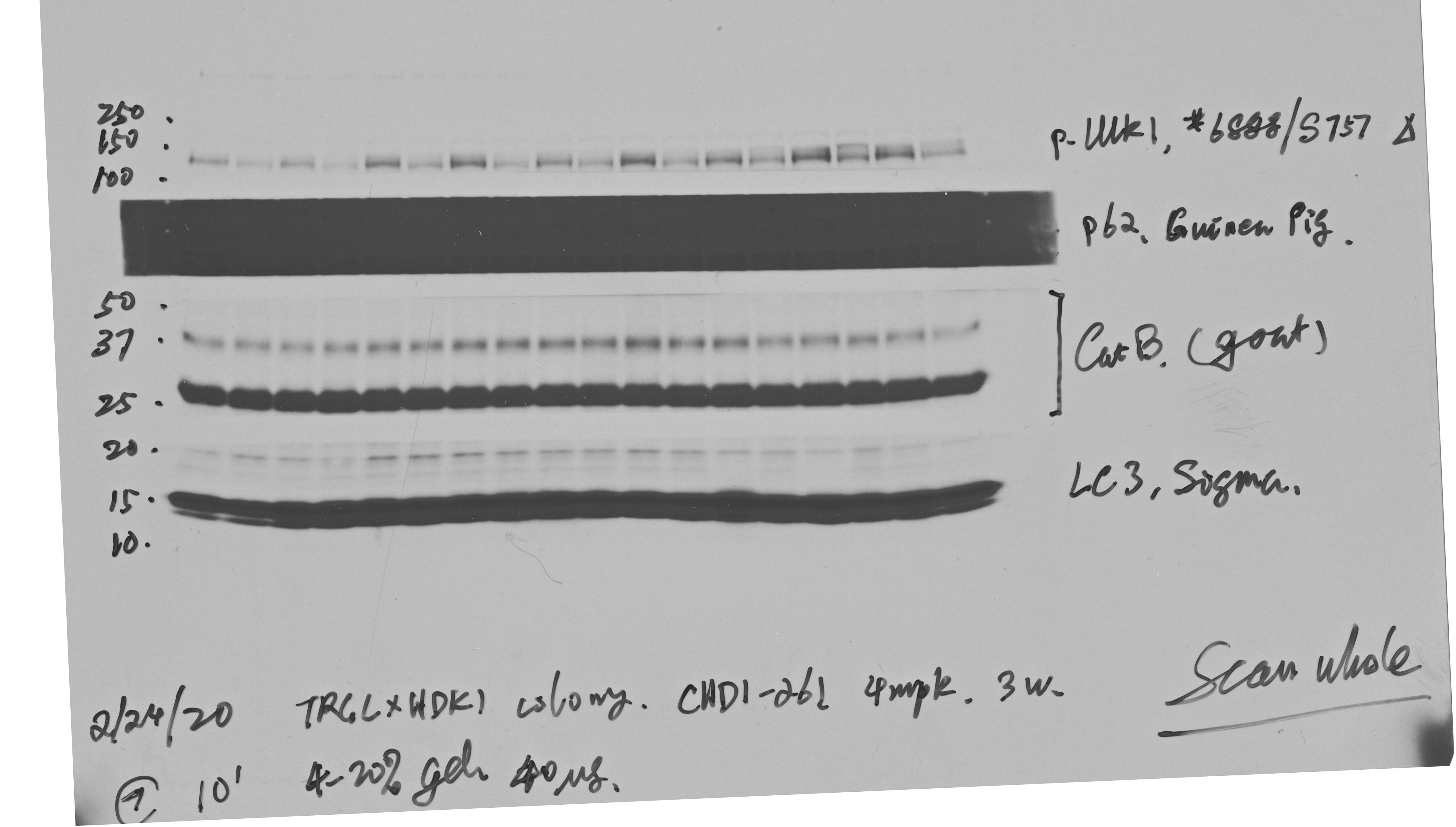

Supplement: Figure 5—source data 1. [file elife-104979-fig5-data1.zip › Figure 5A-Source Data 1/Fig 5A_Anti-p-ULK1 (top).jpg]

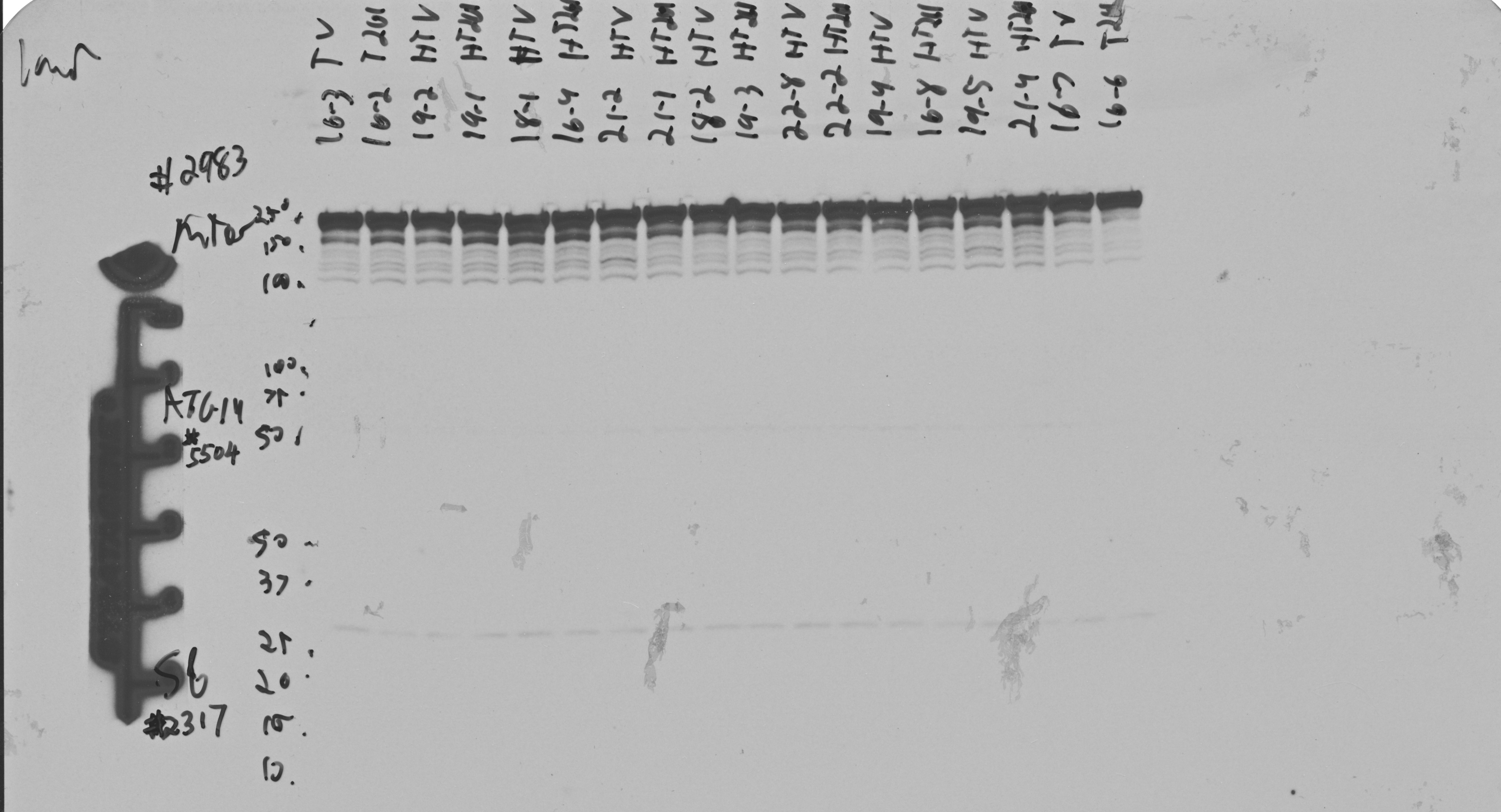

Supplement: Figure 5—source data 1. [file elife-104979-fig5-data1.zip › Figure 5A-Source Data 1/Fig 5A_Anti-S6 (bottom)(too weak)_see long expos on the Labeled file.jpg]

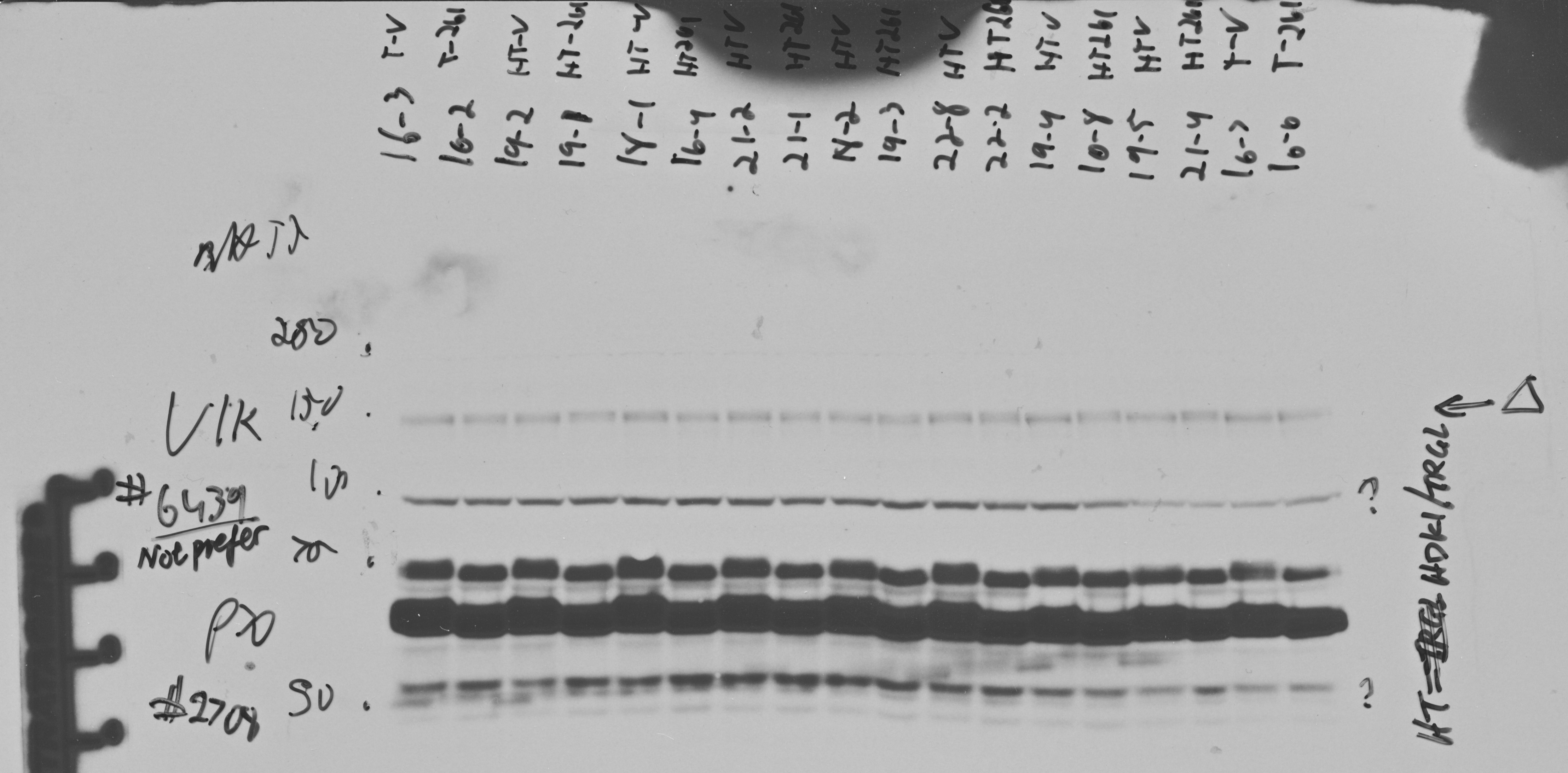

Supplement: Figure 5—source data 1. [file elife-104979-fig5-data1.zip › Figure 5A-Source Data 1/Fig 5A_Anti-ULK1 (top).jpg]

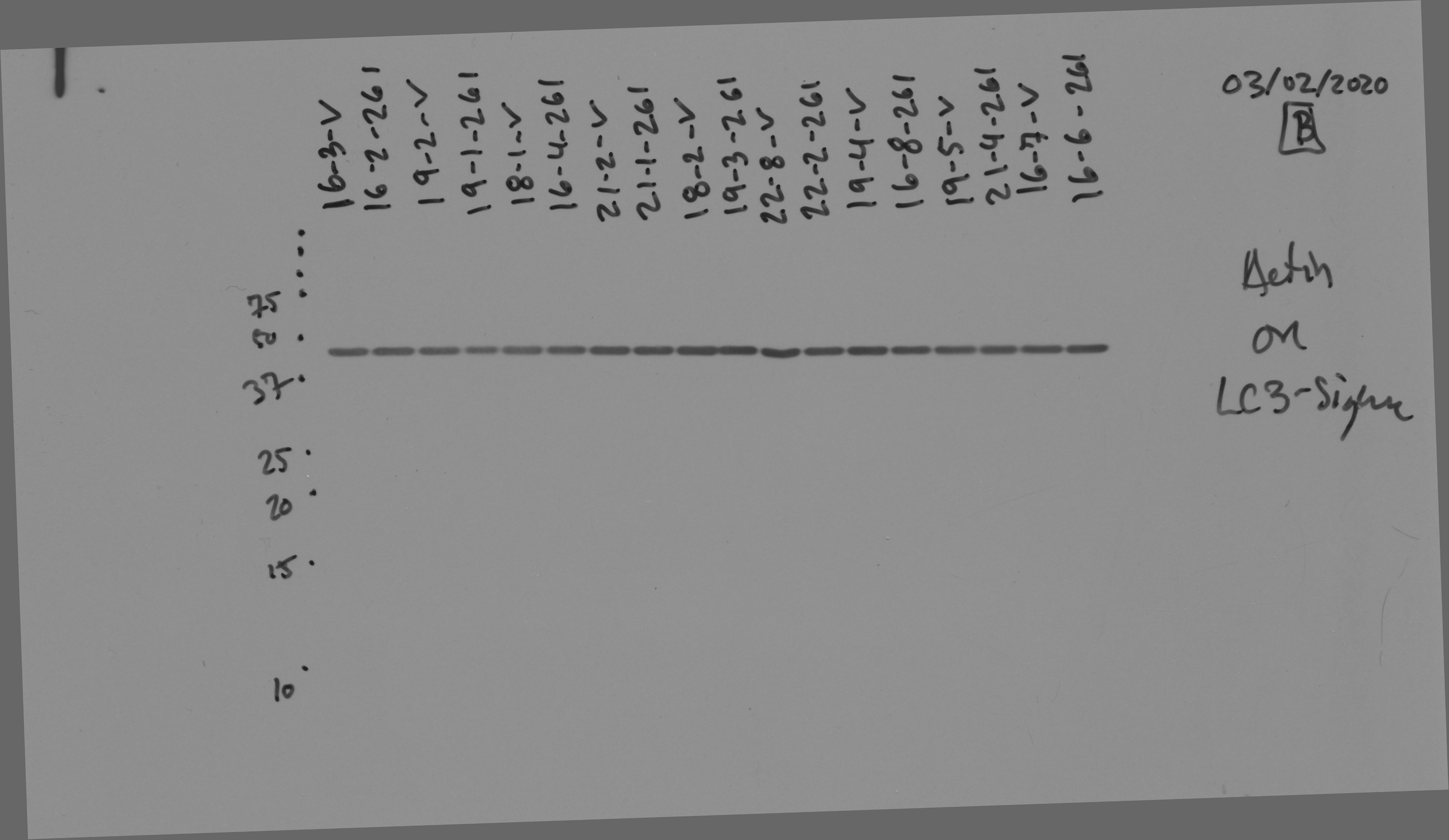

Supplement: Figure 5—source data 1. [file elife-104979-fig5-data1.zip › Figure 5A-Source Data 1/LC3 only/Fig 5A_15% gel_Anti-actin.jpg]

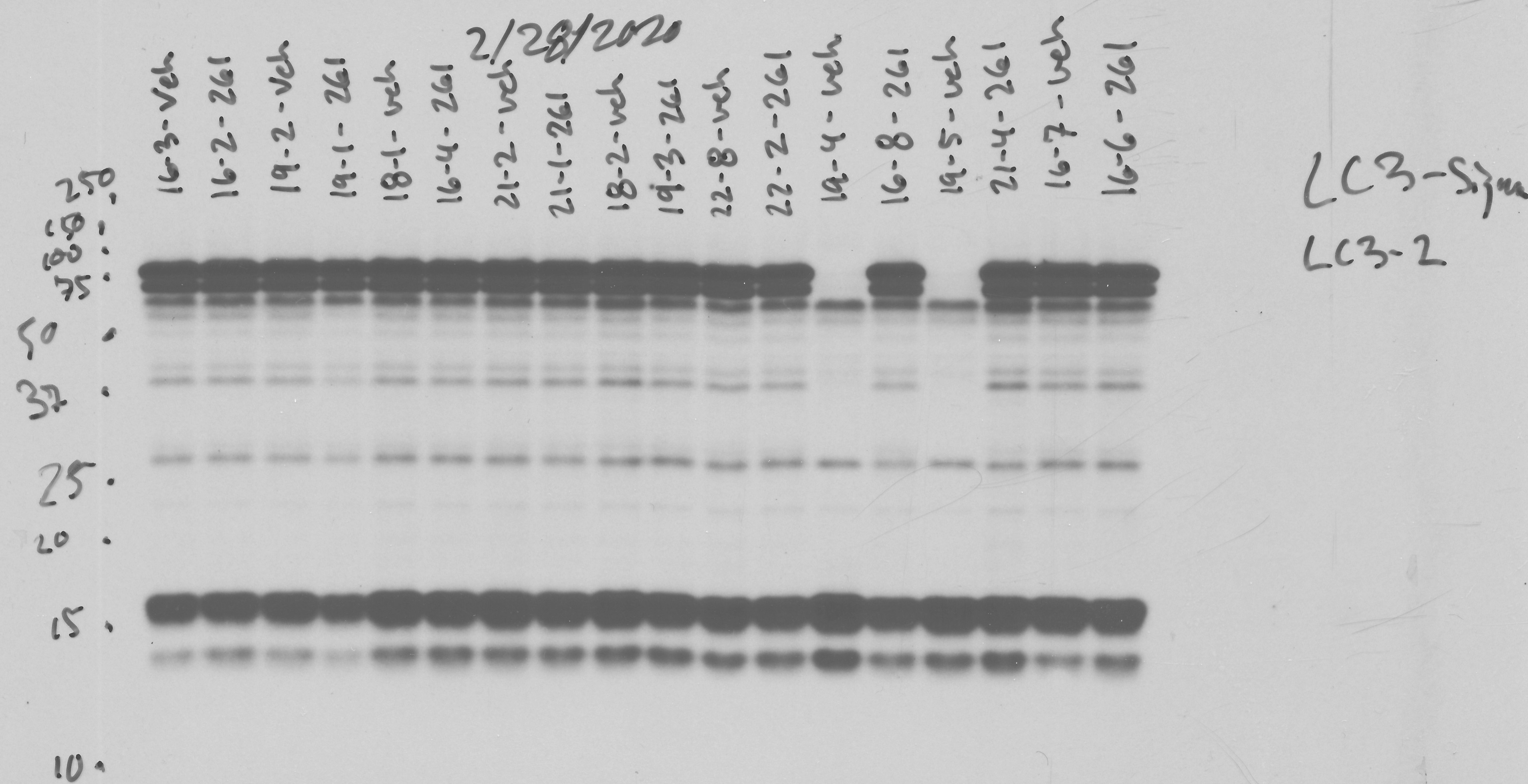

Supplement: Figure 5—source data 1. [file elife-104979-fig5-data1.zip › Figure 5A-Source Data 1/LC3 only/Fig 5A_15% gel_Anti-LC3_long expos.jpg]

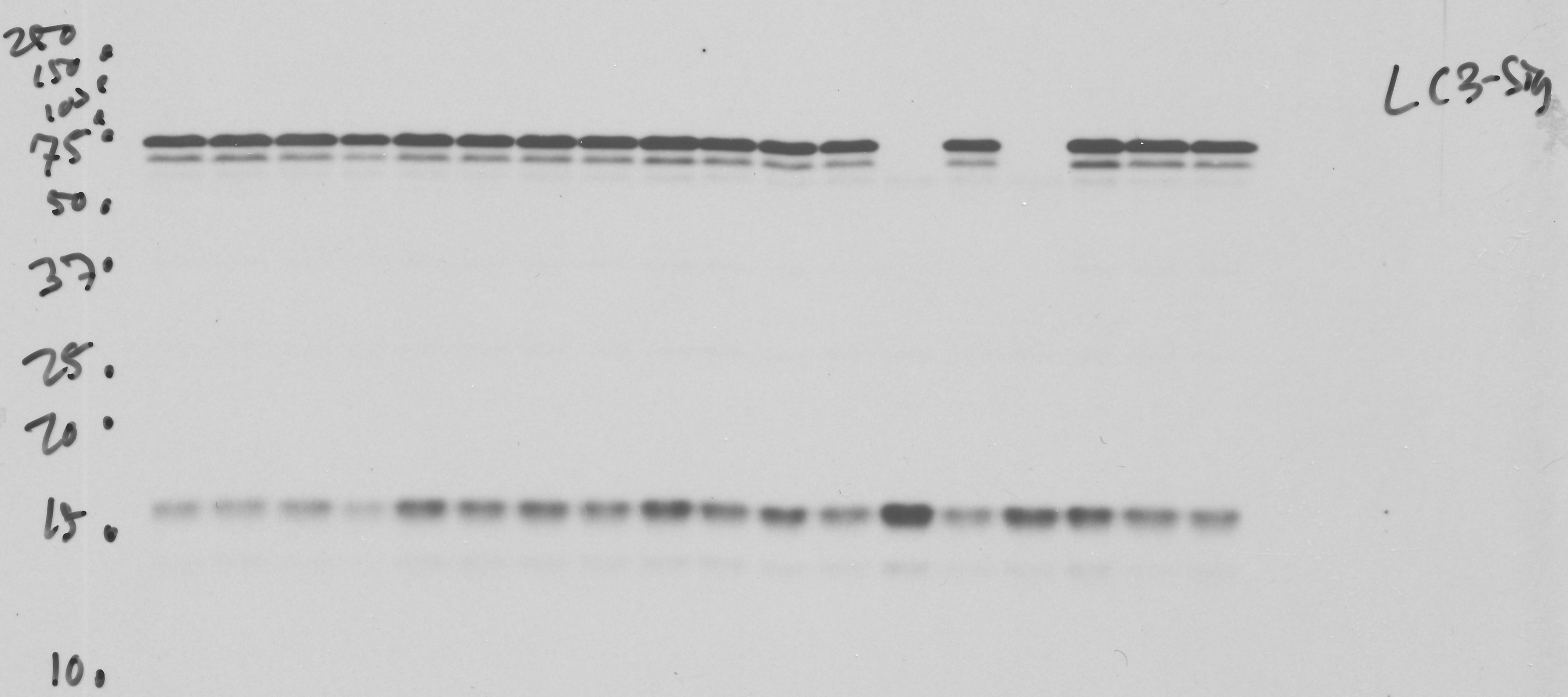

Supplement: Figure 5—source data 1. [file elife-104979-fig5-data1.zip › Figure 5A-Source Data 1/LC3 only/Fig 5A_15% gel_Anti-LC3_short expos.jpg]

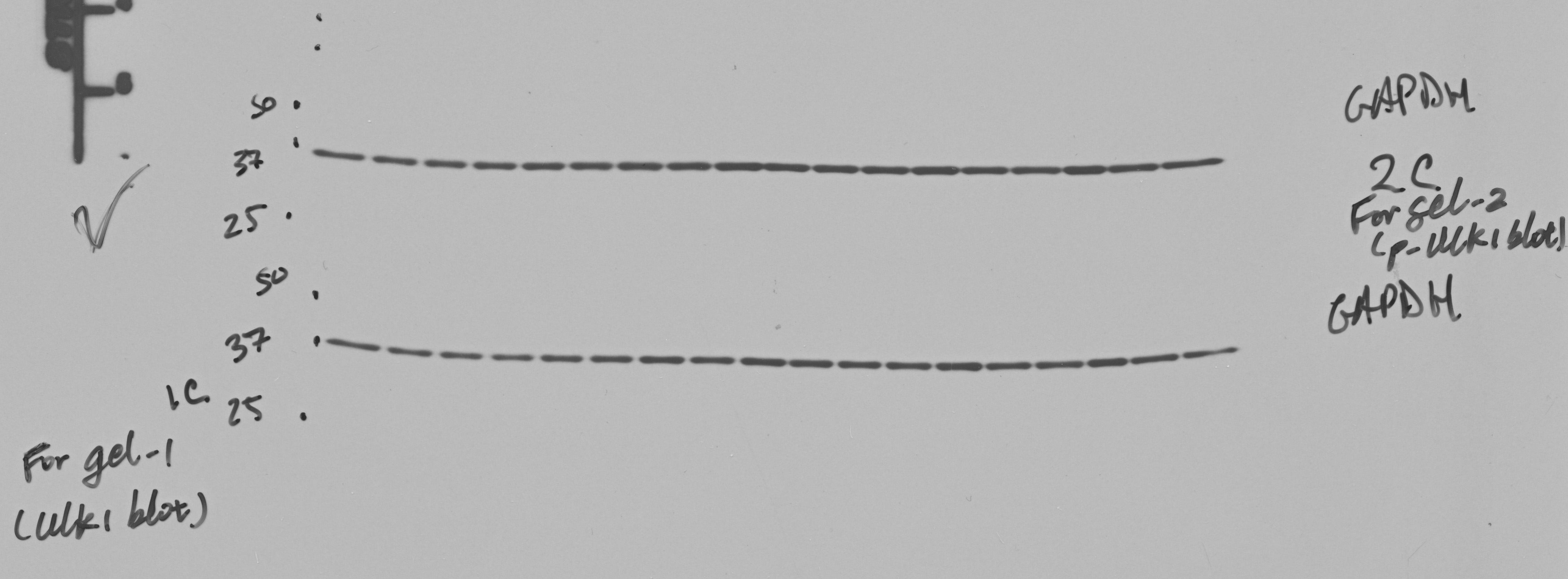

Supplement: Figure 5—source data 1. [file elife-104979-fig5-data1.zip › Figure 5A-Source Data 1/LC3 only/Fig 5A_4-20% gel_Anti-GAPDH_top.jpg]

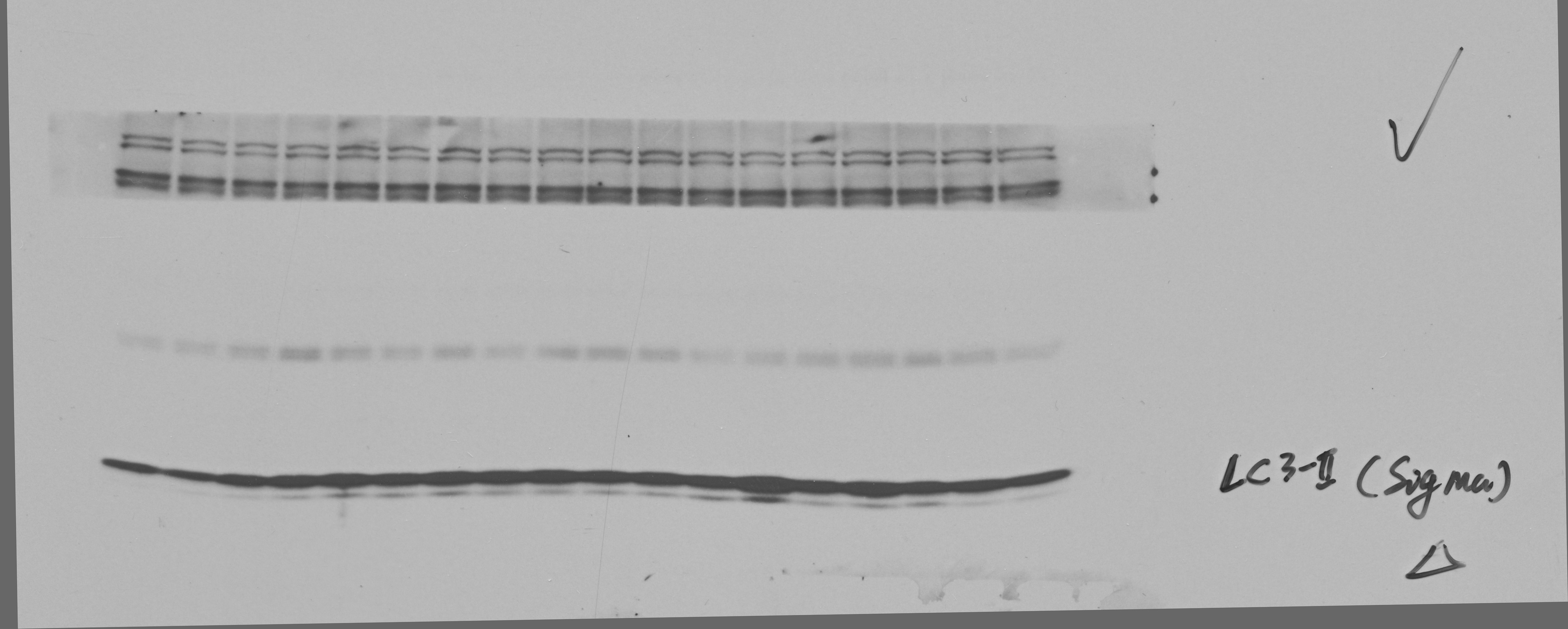

Supplement: Figure 5—source data 1. [file elife-104979-fig5-data1.zip › Figure 5A-Source Data 1/LC3 only/Fig 5A_4-20% gel_Anti-LC3_long expos.jpg]

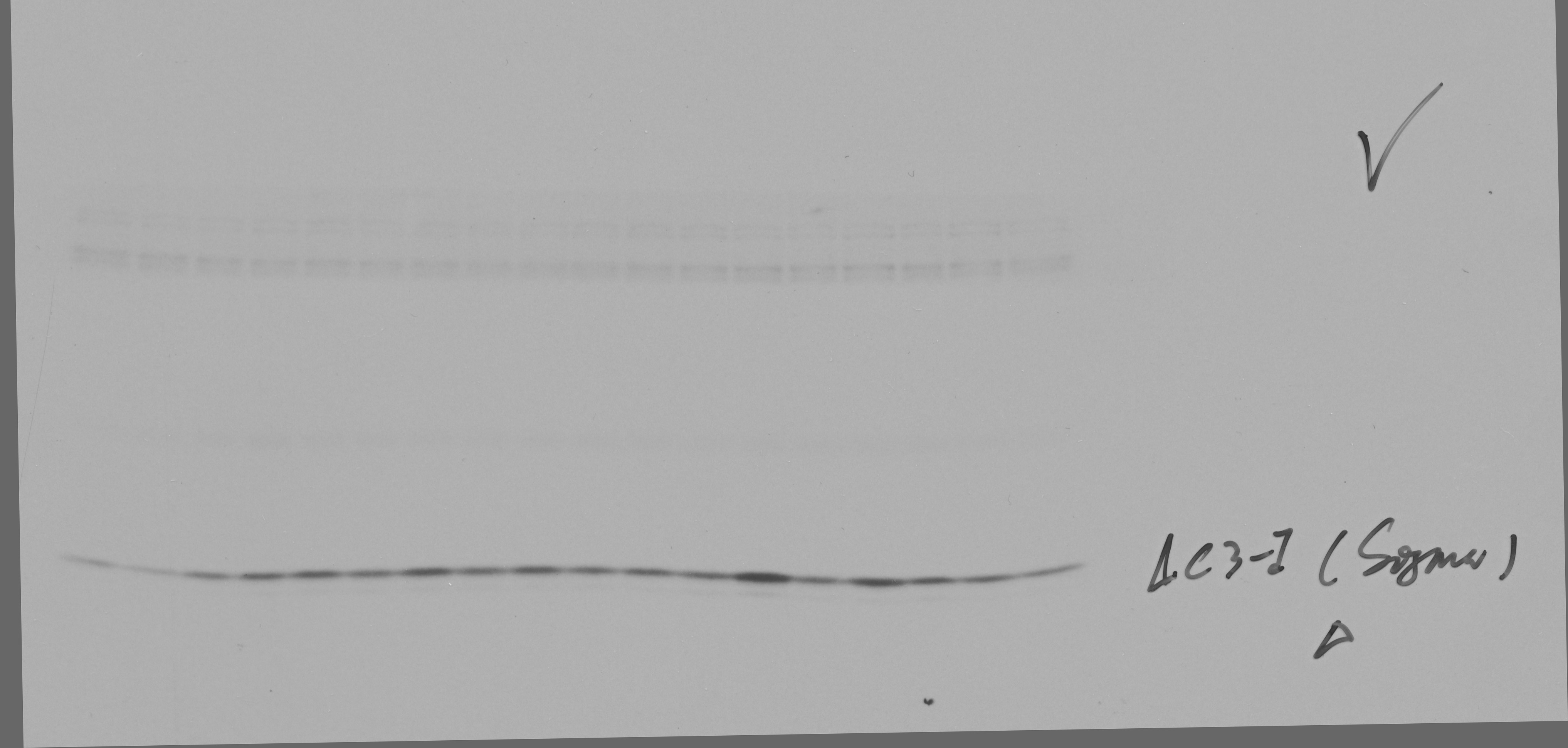

Supplement: Figure 5—source data 1. [file elife-104979-fig5-data1.zip › Figure 5A-Source Data 1/LC3 only/Fig 5A_4-20% gel_Anti-LC3_short expos.jpg]

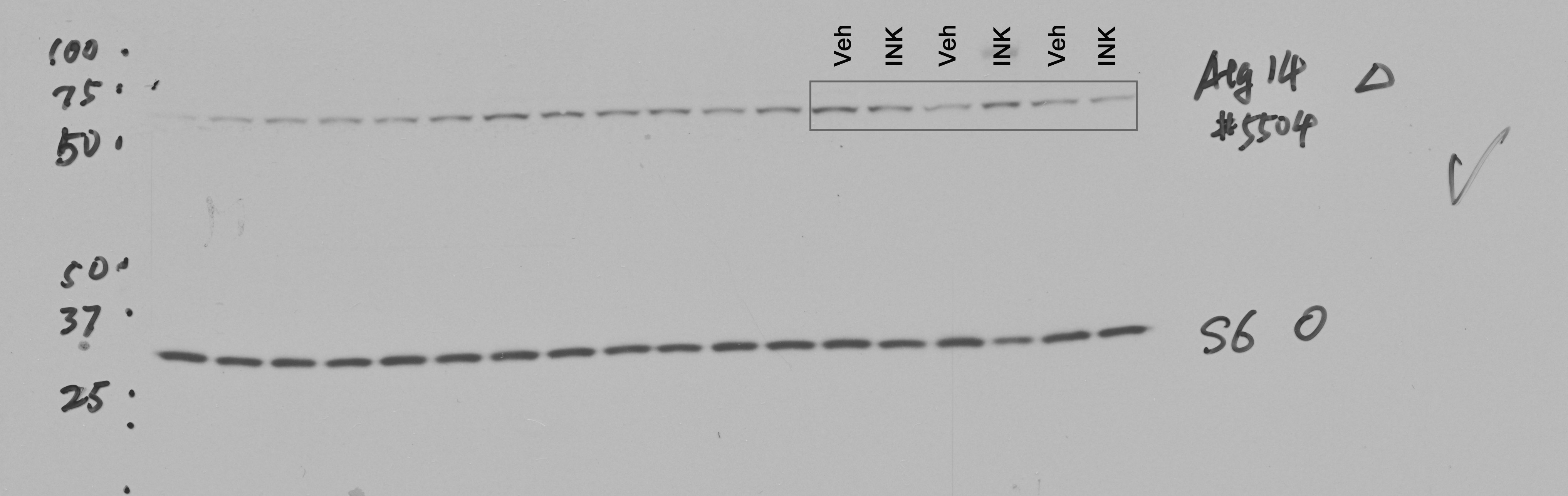

Supplement: Figure 5—source data 2. [file elife-104979-fig5-data2.zip › Figure 5A-Source Data 2/Fig 5A_Anti-Atg14_Long expos & Labeled.jpg]

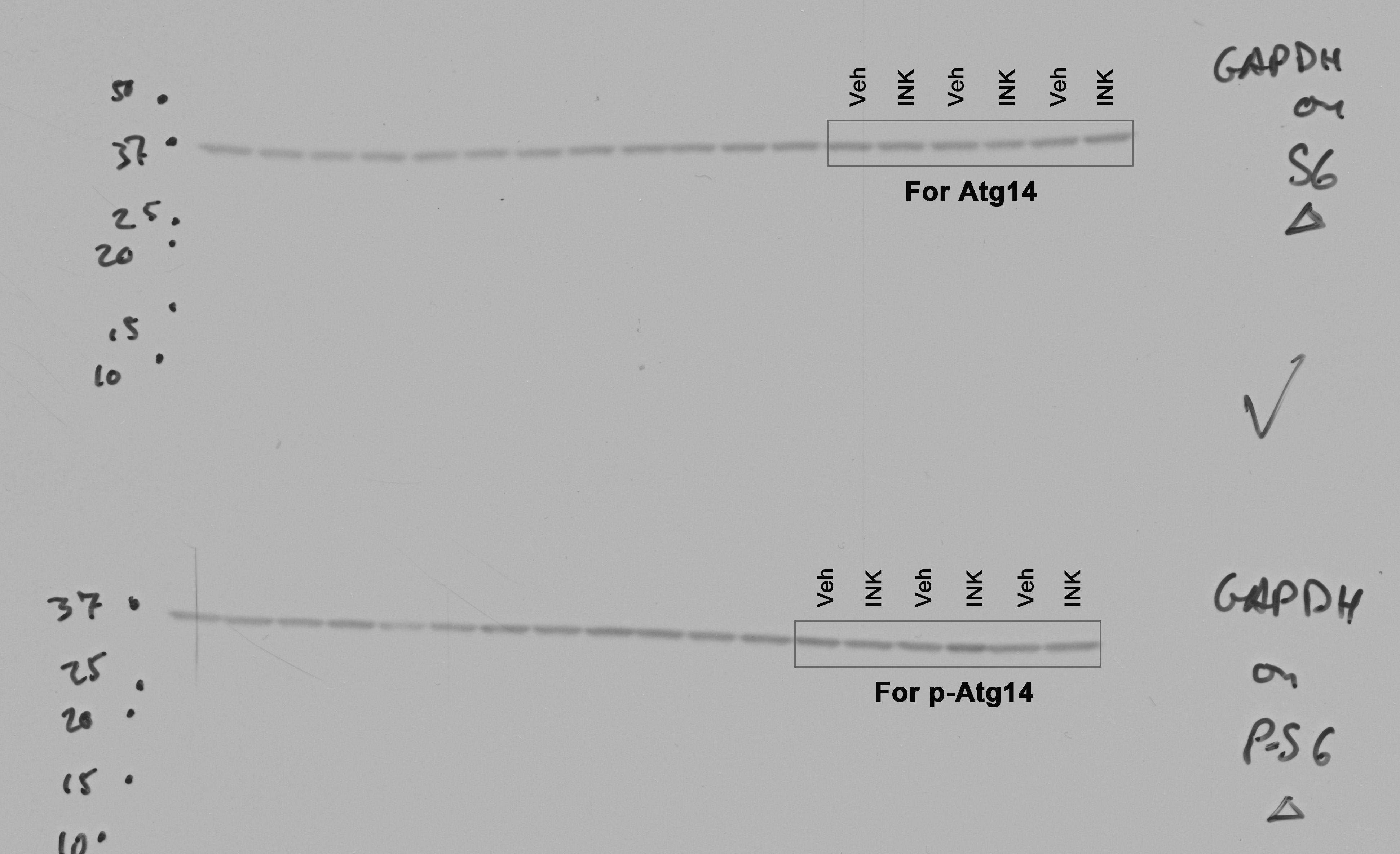

Supplement: Figure 5—source data 2. [file elife-104979-fig5-data2.zip › Figure 5A-Source Data 2/Fig 5A_Anti-GAPDH)_top for Atg14 (& S6), bottom for p-Atg14 (& p-S6)_Labeled.jpg]

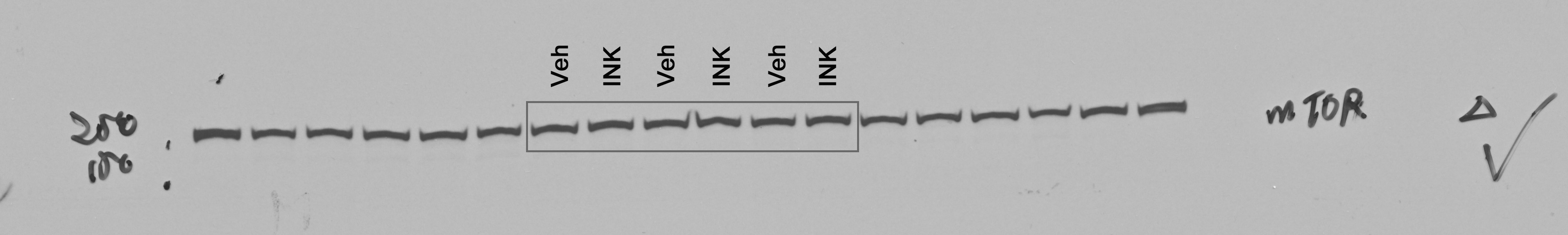

Supplement: Figure 5—source data 2. [file elife-104979-fig5-data2.zip › Figure 5A-Source Data 2/Fig 5A_Anti-mTOR_Labeled.jpg]

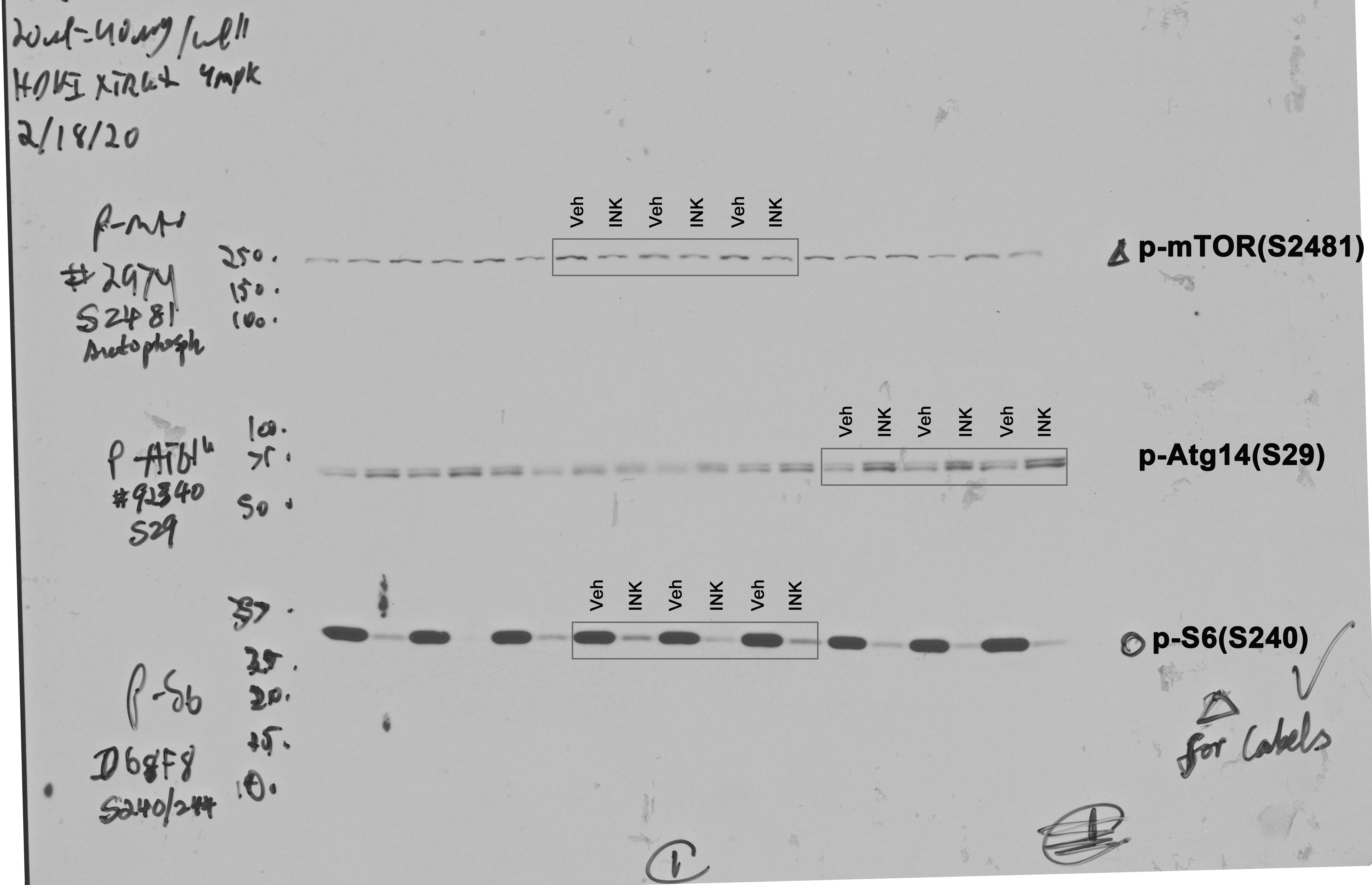

Supplement: Figure 5—source data 2. [file elife-104979-fig5-data2.zip › Figure 5A-Source Data 2/Fig 5A_Anti-p-mTOR (top), anti-p-Atg14 (middle) & anti-p-S6 (bottom)_Labeled.jpg]

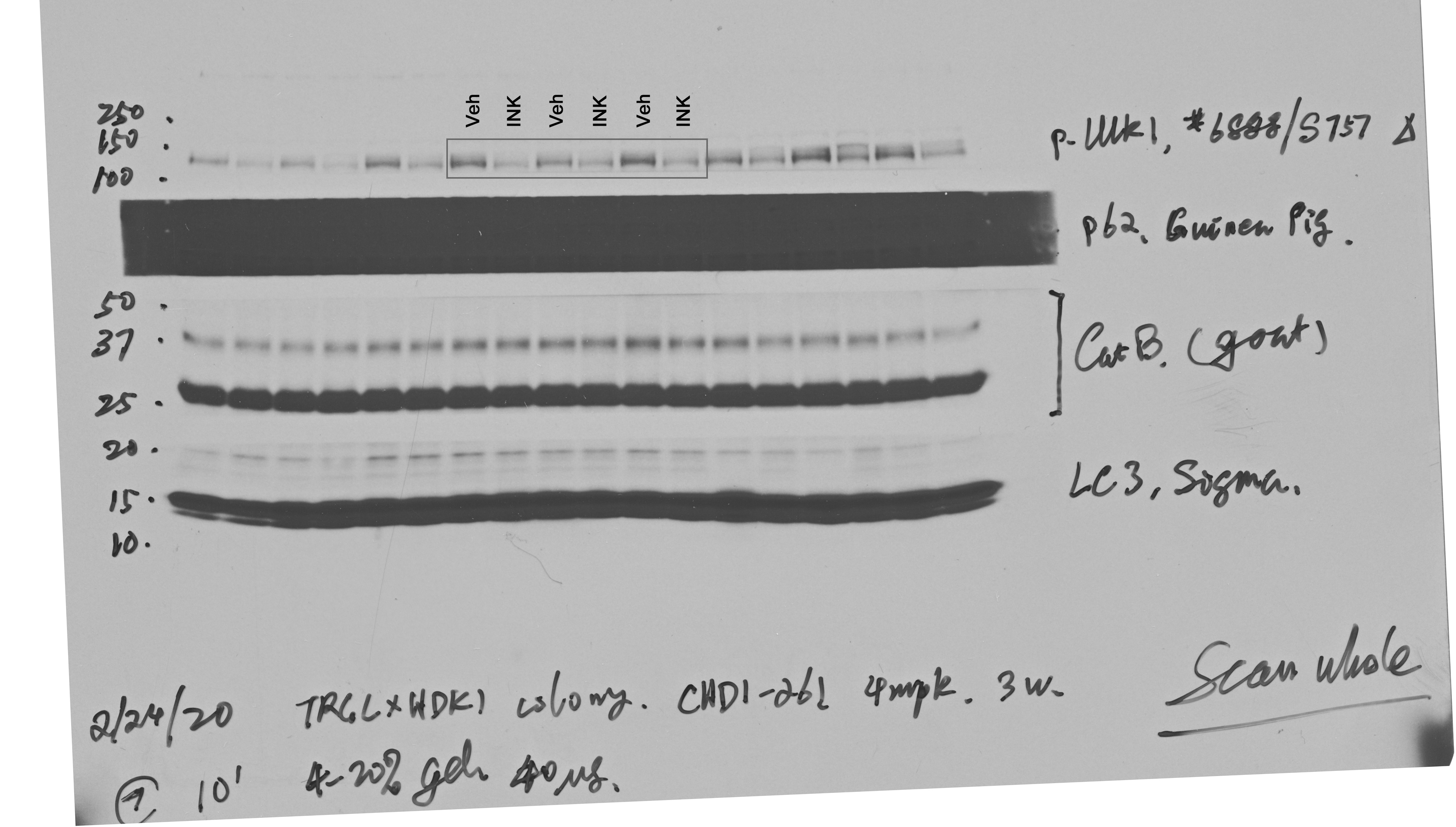

Supplement: Figure 5—source data 2. [file elife-104979-fig5-data2.zip › Figure 5A-Source Data 2/Fig 5A_Anti-p-ULK1 (top)_Labeled.jpg]

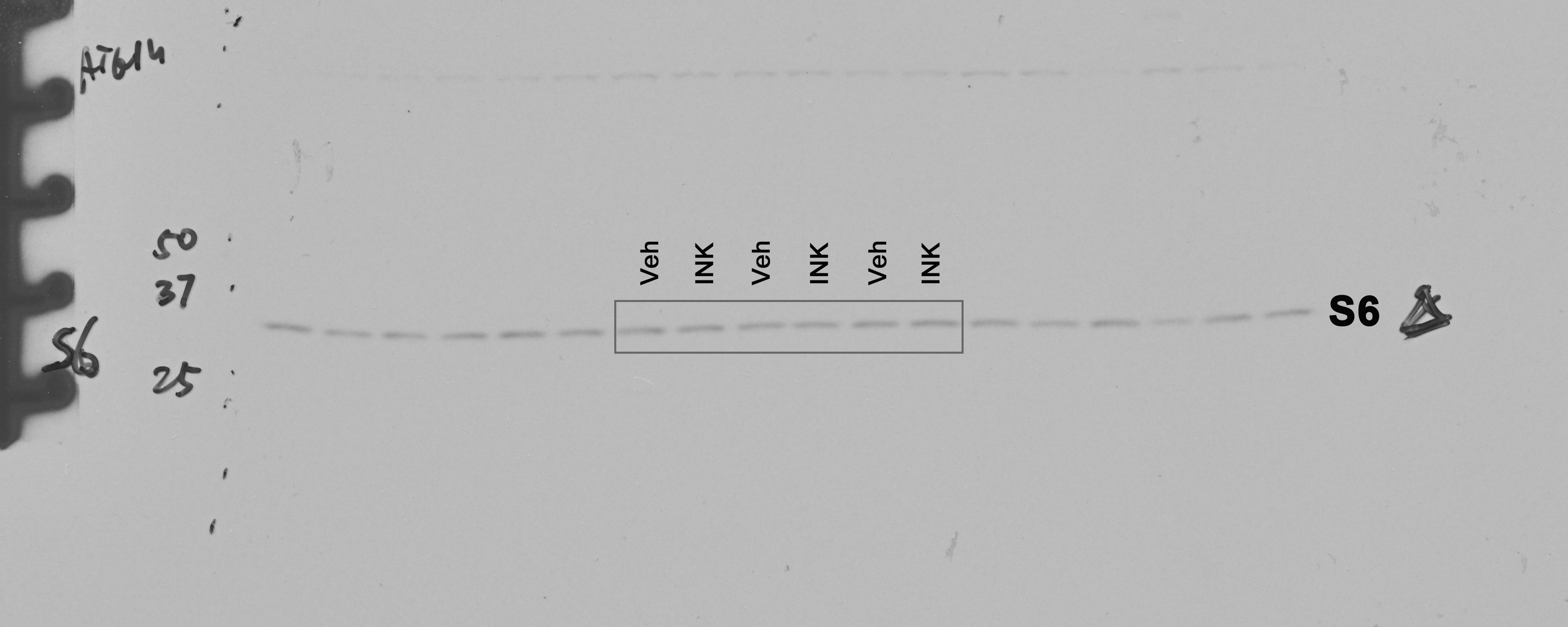

Supplement: Figure 5—source data 2. [file elife-104979-fig5-data2.zip › Figure 5A-Source Data 2/Fig 5A_Anti-S6 (bottom)_Long expos & Labeled.jpg]

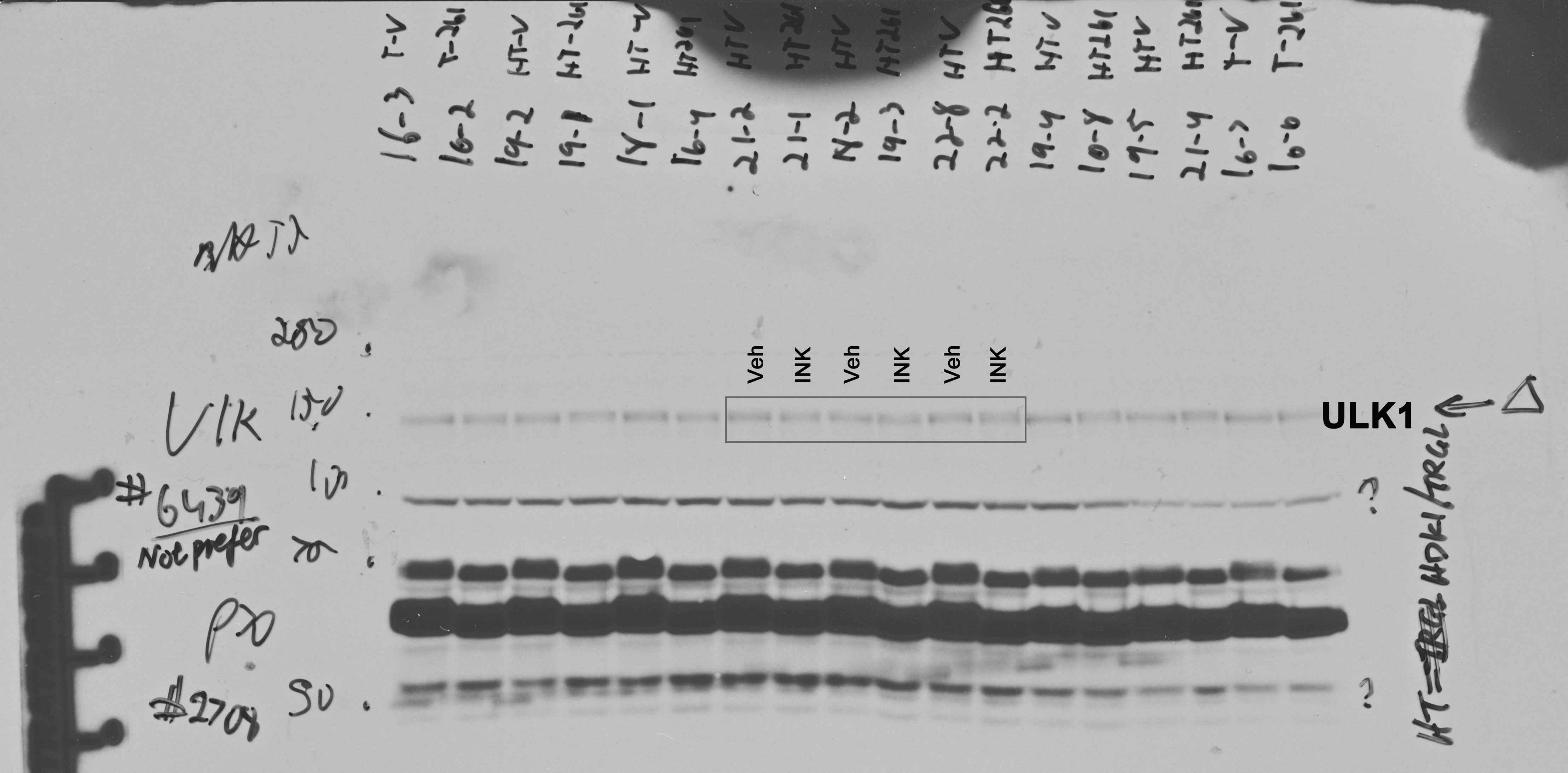

Supplement: Figure 5—source data 2. [file elife-104979-fig5-data2.zip › Figure 5A-Source Data 2/Fig 5A_Anti-ULK1 (top)_Labeled.jpg]

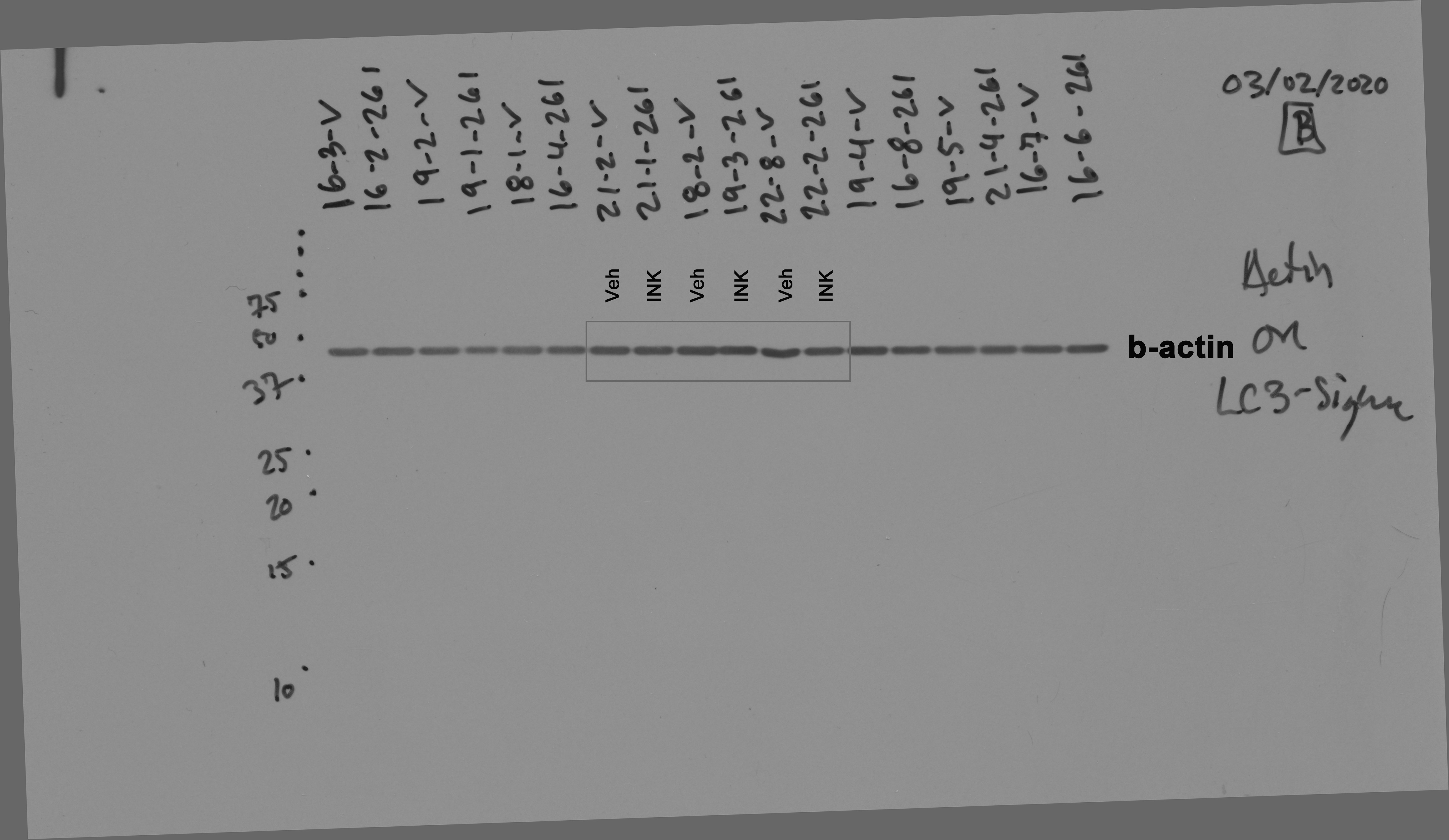

Supplement: Figure 5—source data 2. [file elife-104979-fig5-data2.zip › Figure 5A-Source Data 2/LC3 only/Fig 5A_15% gel_Anti-actin_Labeled.jpg]

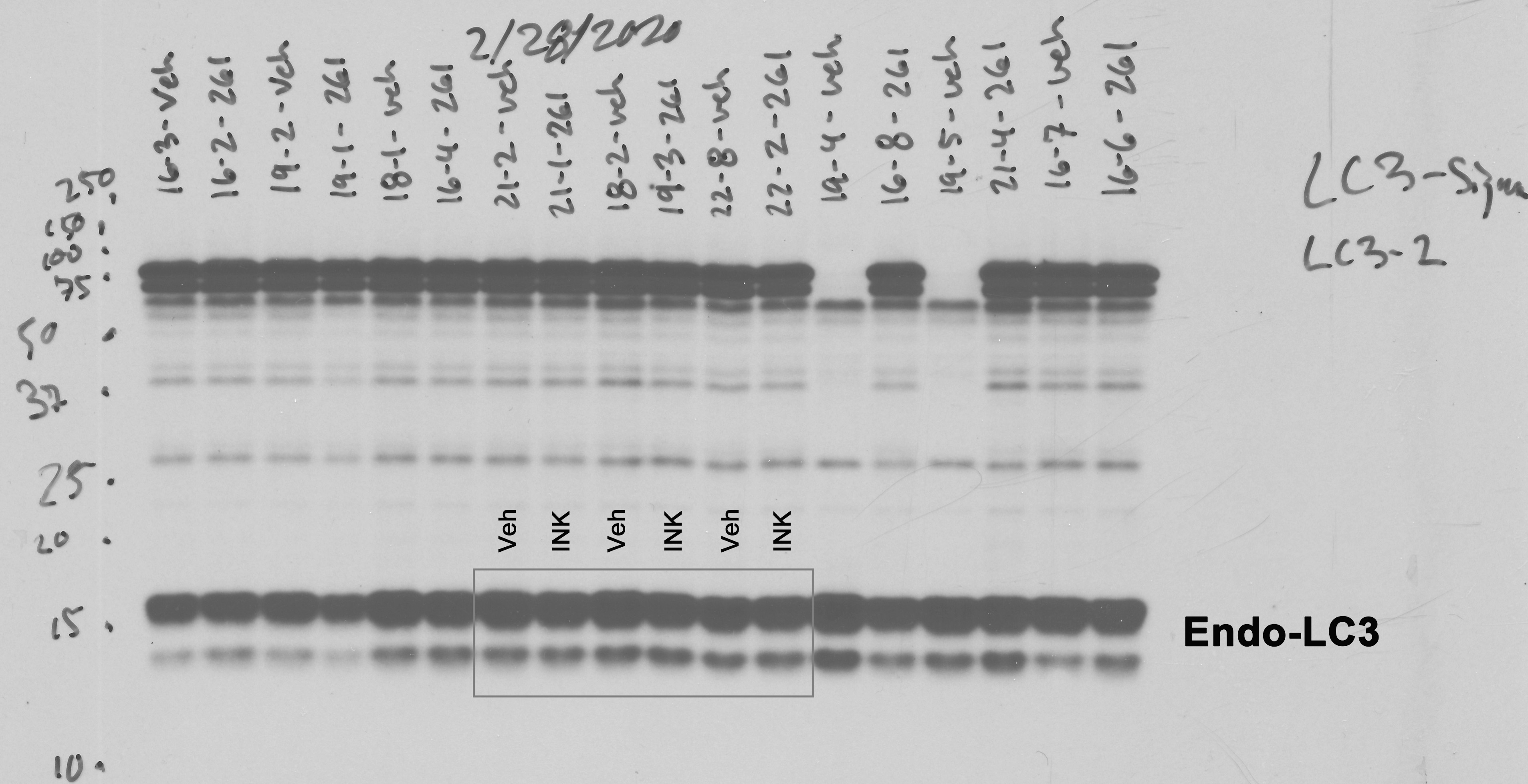

Supplement: Figure 5—source data 2. [file elife-104979-fig5-data2.zip › Figure 5A-Source Data 2/LC3 only/Fig 5A_15% gel_Anti-LC3_long expos_Labeled.jpg]

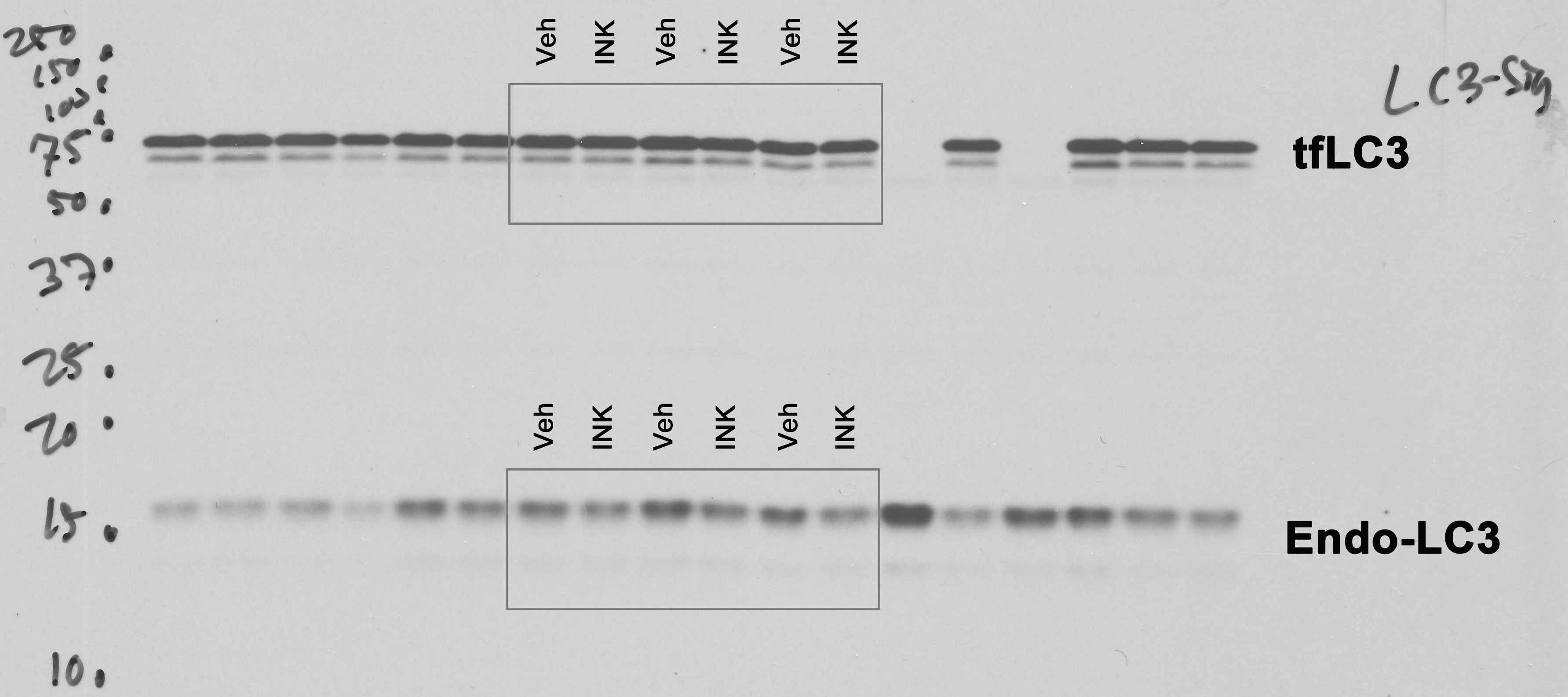

Supplement: Figure 5—source data 2. [file elife-104979-fig5-data2.zip › Figure 5A-Source Data 2/LC3 only/Fig 5A_15% gel_Anti-LC3_short expos_Labeled.jpg]

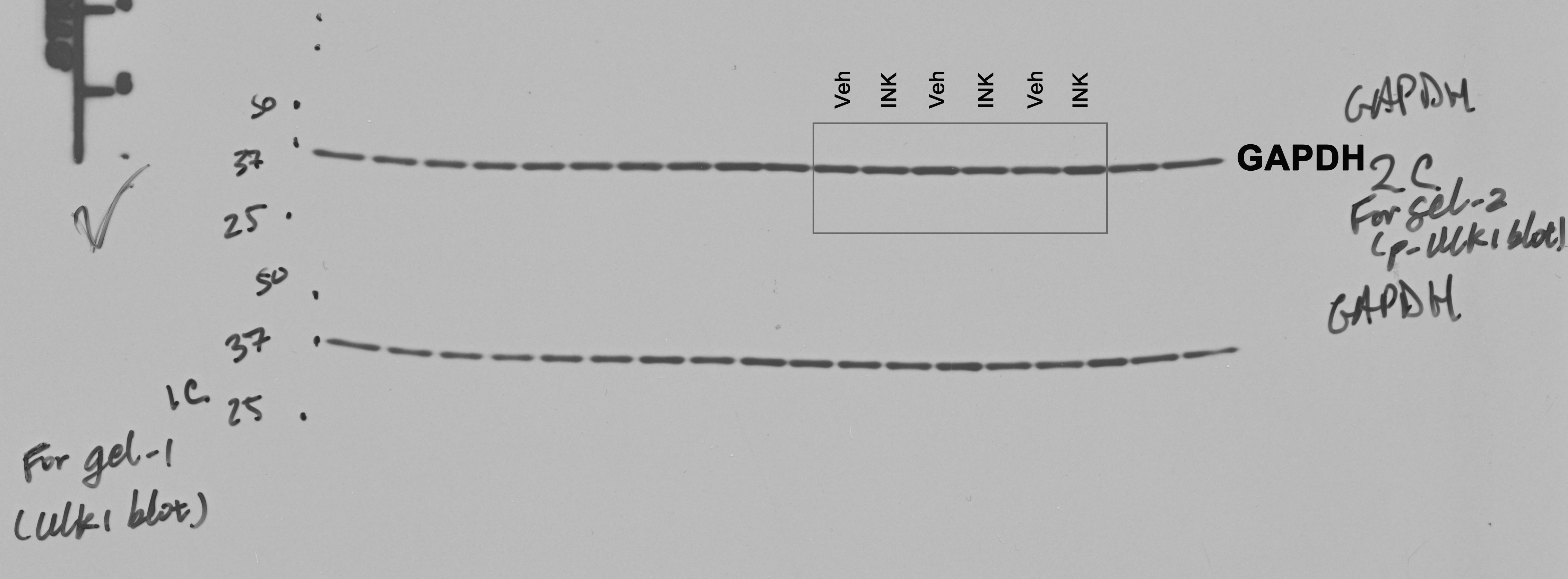

Supplement: Figure 5—source data 2. [file elife-104979-fig5-data2.zip › Figure 5A-Source Data 2/LC3 only/Fig 5A_4-20% gel_Anti-GAPDH_top_Labeled.jpg]

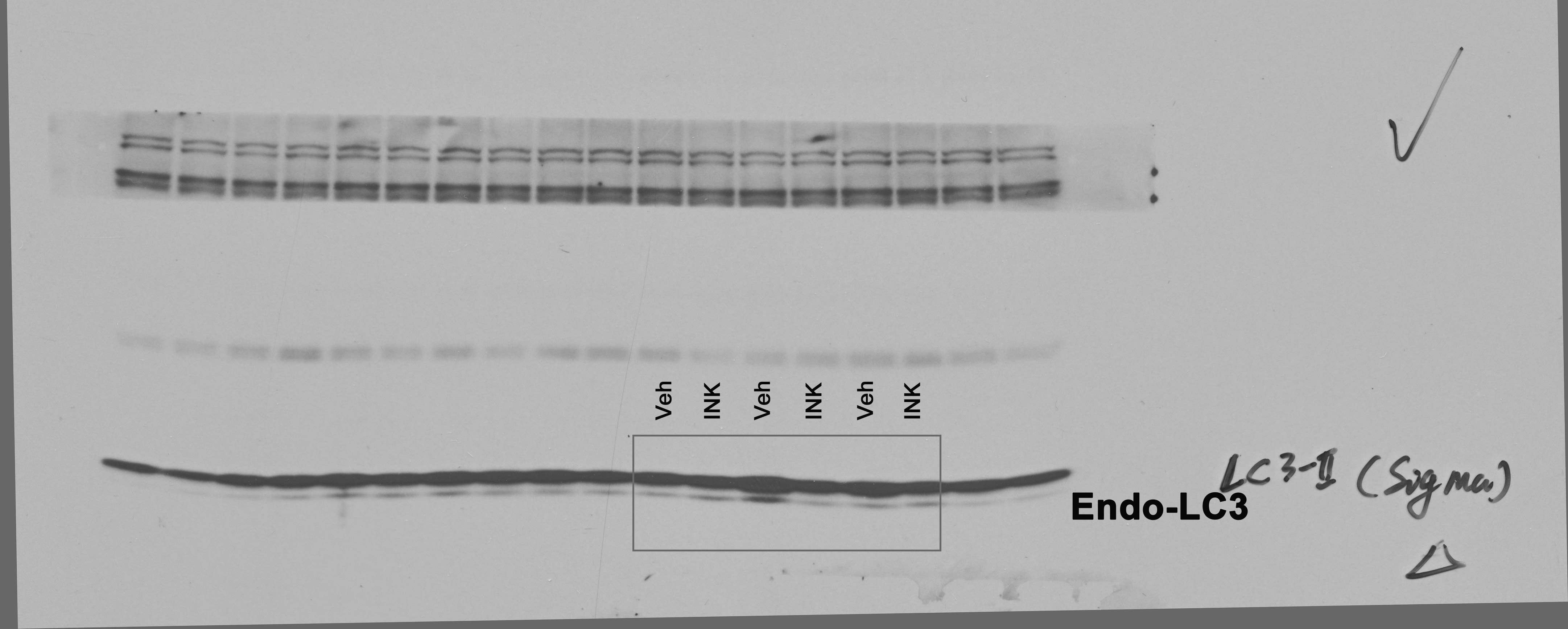

Supplement: Figure 5—source data 2. [file elife-104979-fig5-data2.zip › Figure 5A-Source Data 2/LC3 only/Fig 5A_4-20% gel_Anti-LC3_long expos_Labeled.jpg]

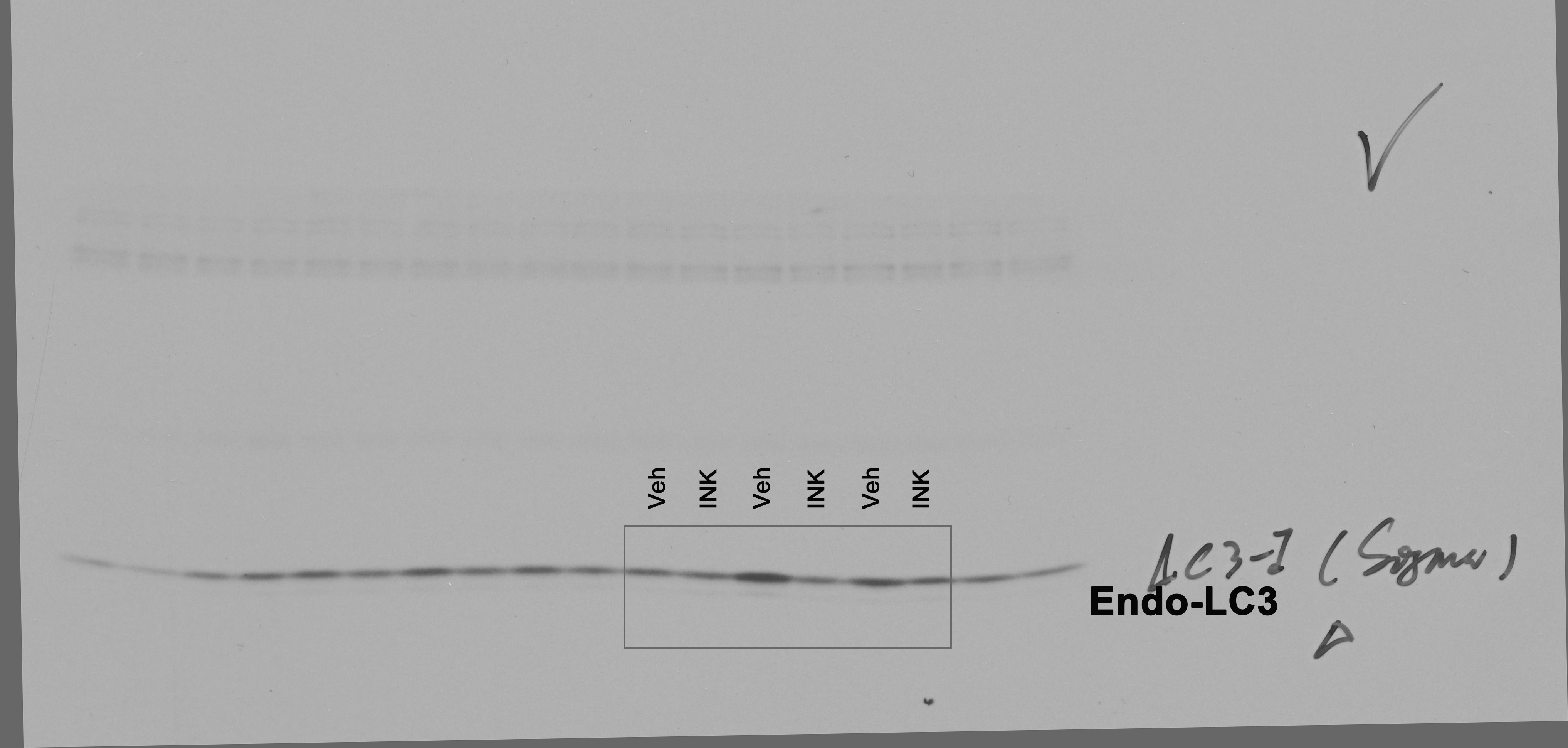

Supplement: Figure 5—source data 2. [file elife-104979-fig5-data2.zip › Figure 5A-Source Data 2/LC3 only/Fig 5A_4-20% gel_Anti-LC3_short expos_Labeled.jpg]

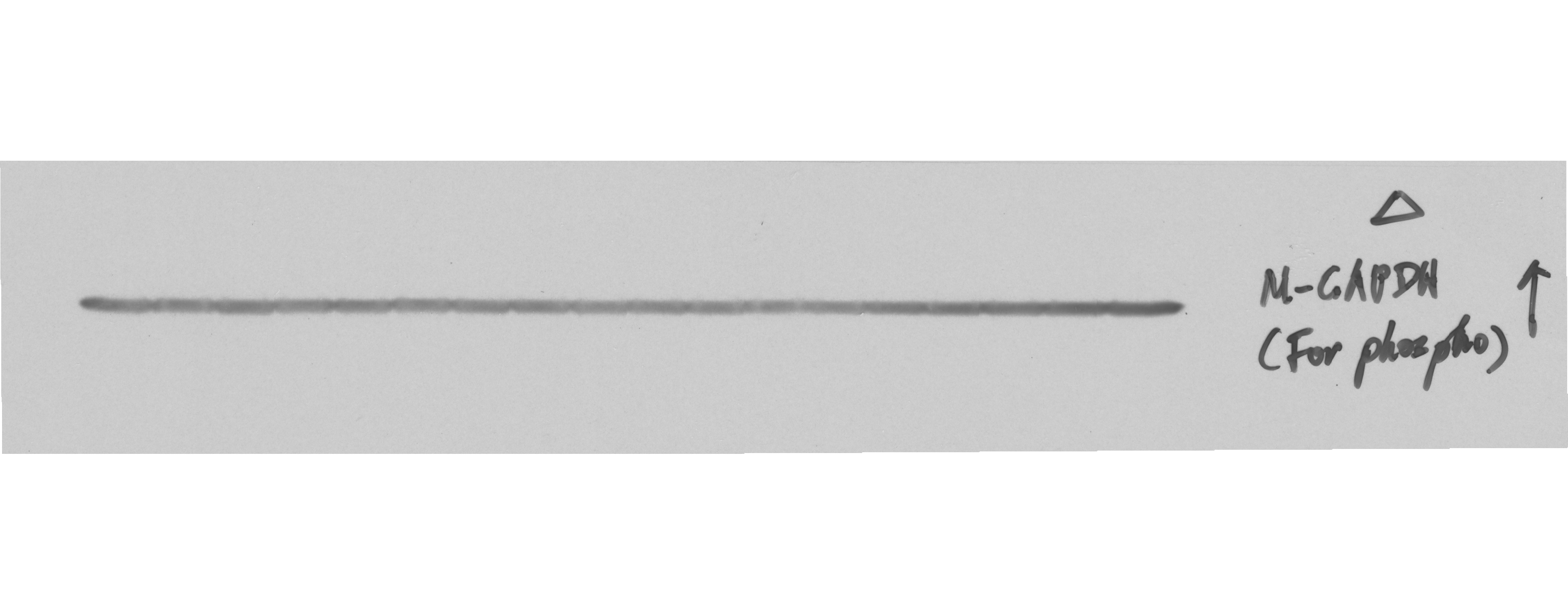

Supplement: Figure 5—figure supplement 1—source data 1. [file elife-104979-fig5-figsupp1-data1.zip › Figure 5-Figure supplement 1-Source Data 1/Blots on the left column/Anti-GAPDH (for p-ULK1 & p-S6).tif]

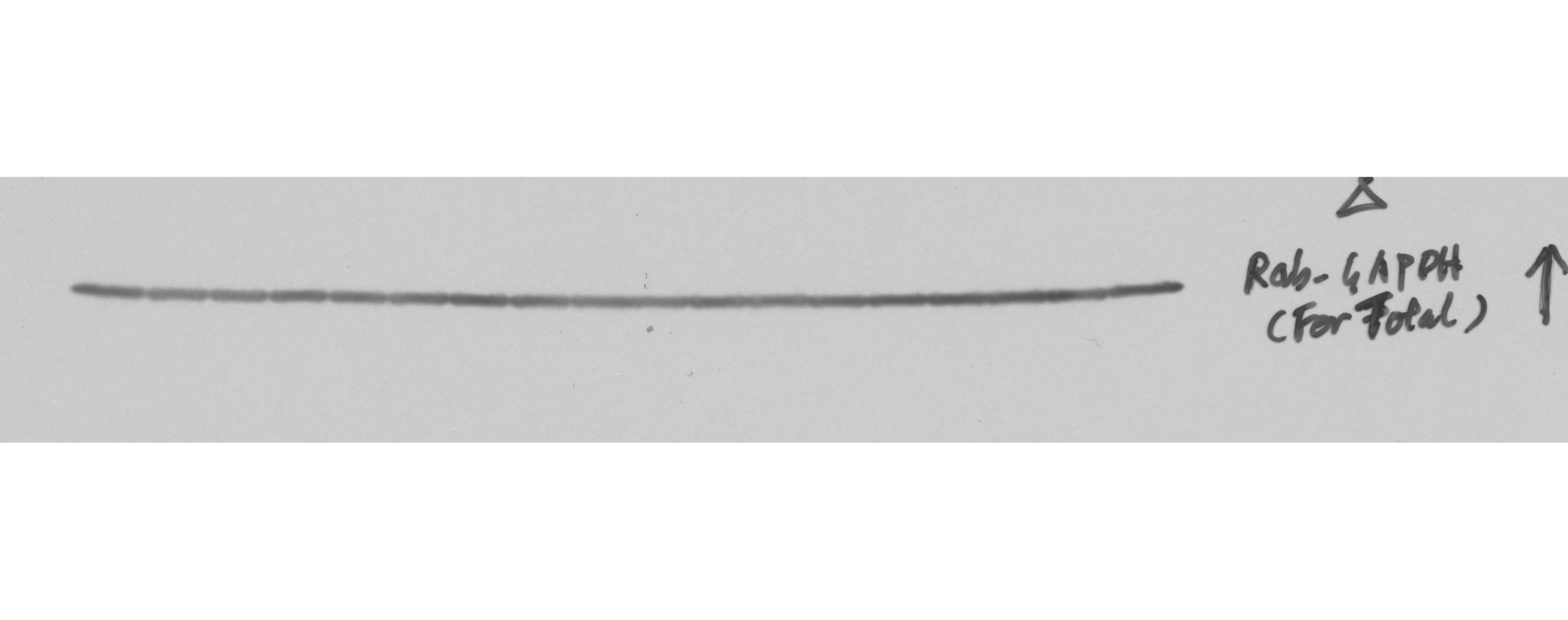

Supplement: Figure 5—figure supplement 1—source data 1. [file elife-104979-fig5-figsupp1-data1.zip › Figure 5-Figure supplement 1-Source Data 1/Blots on the left column/Anti-GAPDH (for ULK1 & S6).tif]

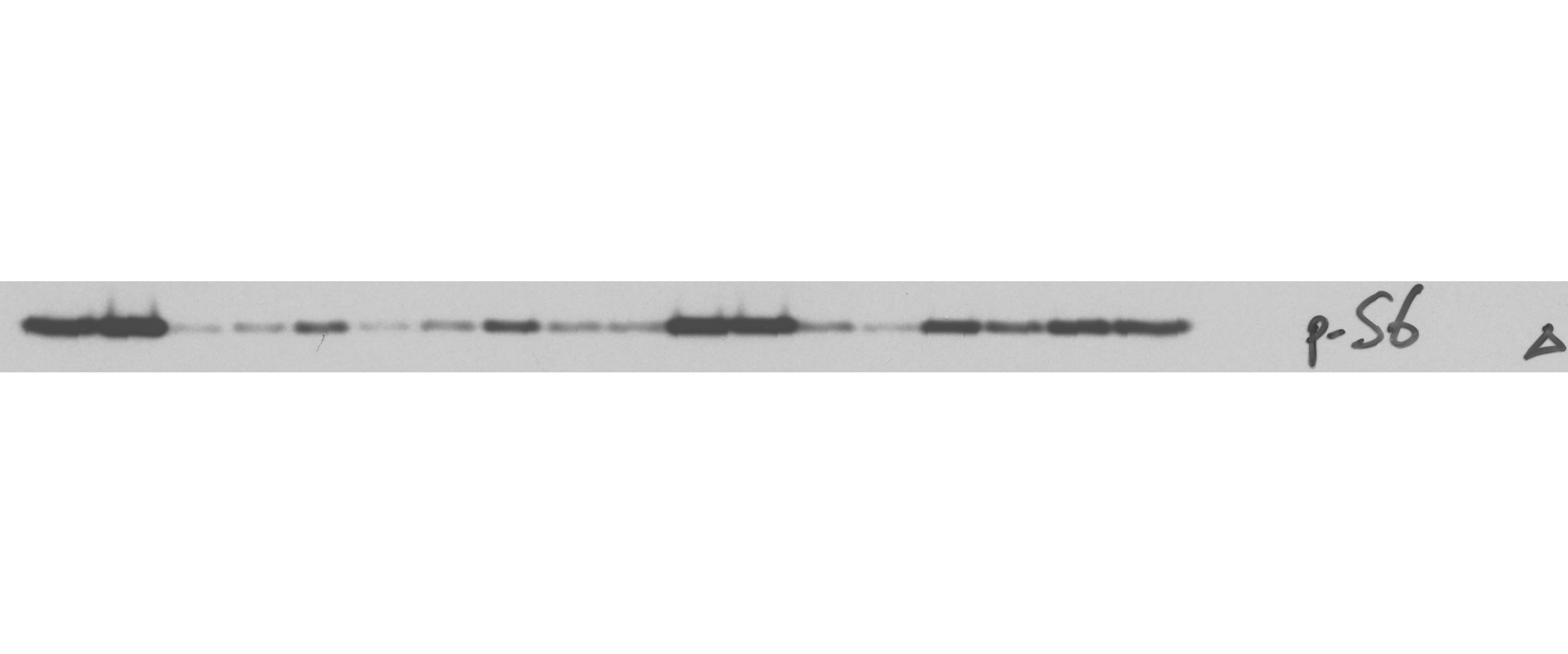

Supplement: Figure 5—figure supplement 1—source data 1. [file elife-104979-fig5-figsupp1-data1.zip › Figure 5-Figure supplement 1-Source Data 1/Blots on the left column/Anti-p-S6 (short expos).tif]

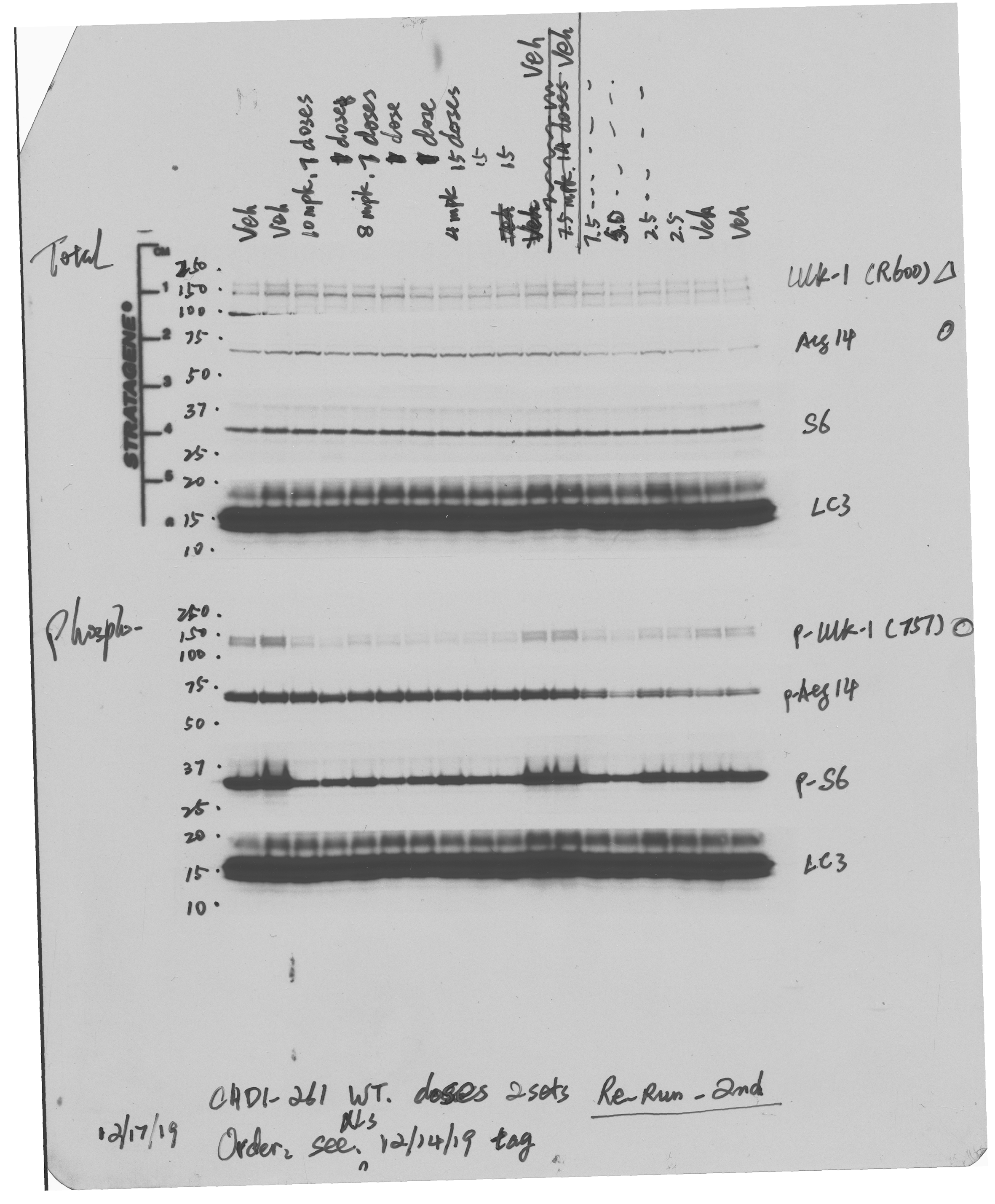

Supplement: Figure 5—figure supplement 1—source data 1. [file elife-104979-fig5-figsupp1-data1.zip › Figure 5-Figure supplement 1-Source Data 1/Blots on the left column/Anti-ULK1, S6, p-ULK1, p-S6.tif]

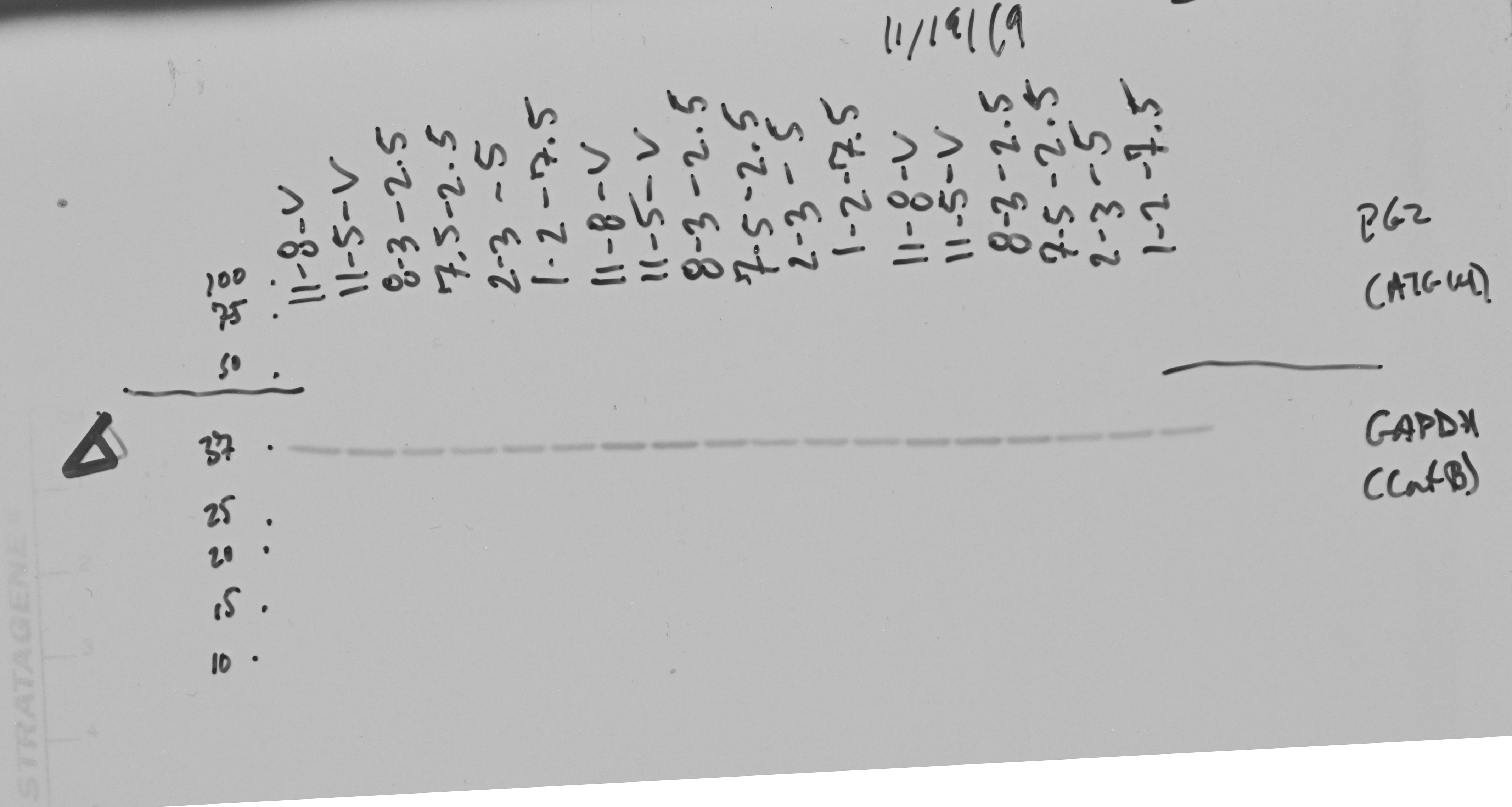

Supplement: Figure 5—figure supplement 1—source data 1. [file elife-104979-fig5-figsupp1-data1.zip › Figure 5-Figure supplement 1-Source Data 1/Blots on the right column/Anti-GAPDH (for ULK1).tif]

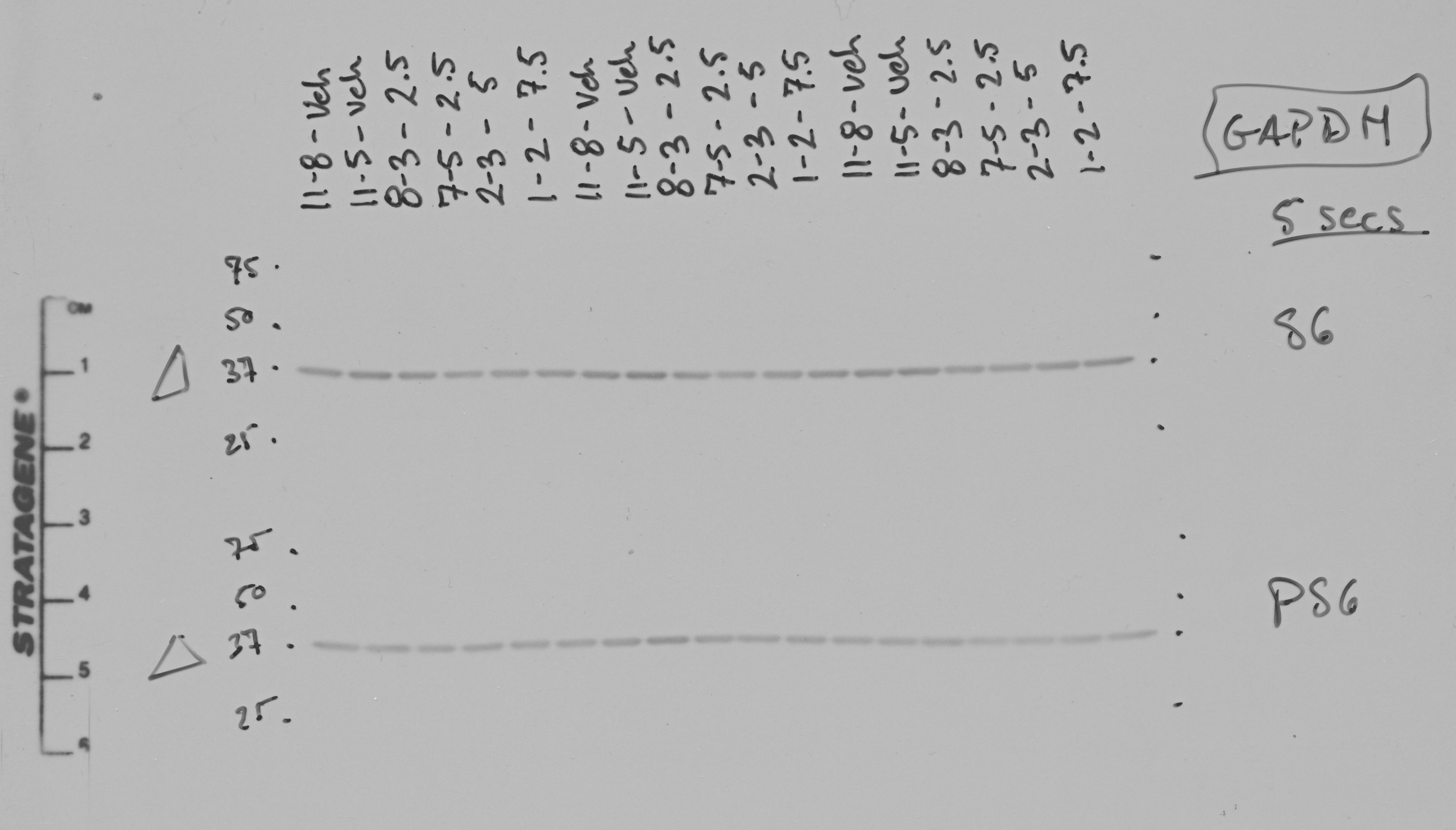

Supplement: Figure 5—figure supplement 1—source data 1. [file elife-104979-fig5-figsupp1-data1.zip › Figure 5-Figure supplement 1-Source Data 1/Blots on the right column/Anti-GAPDH (top for S6, bottom for p-ULK1 & p-S6).tif]

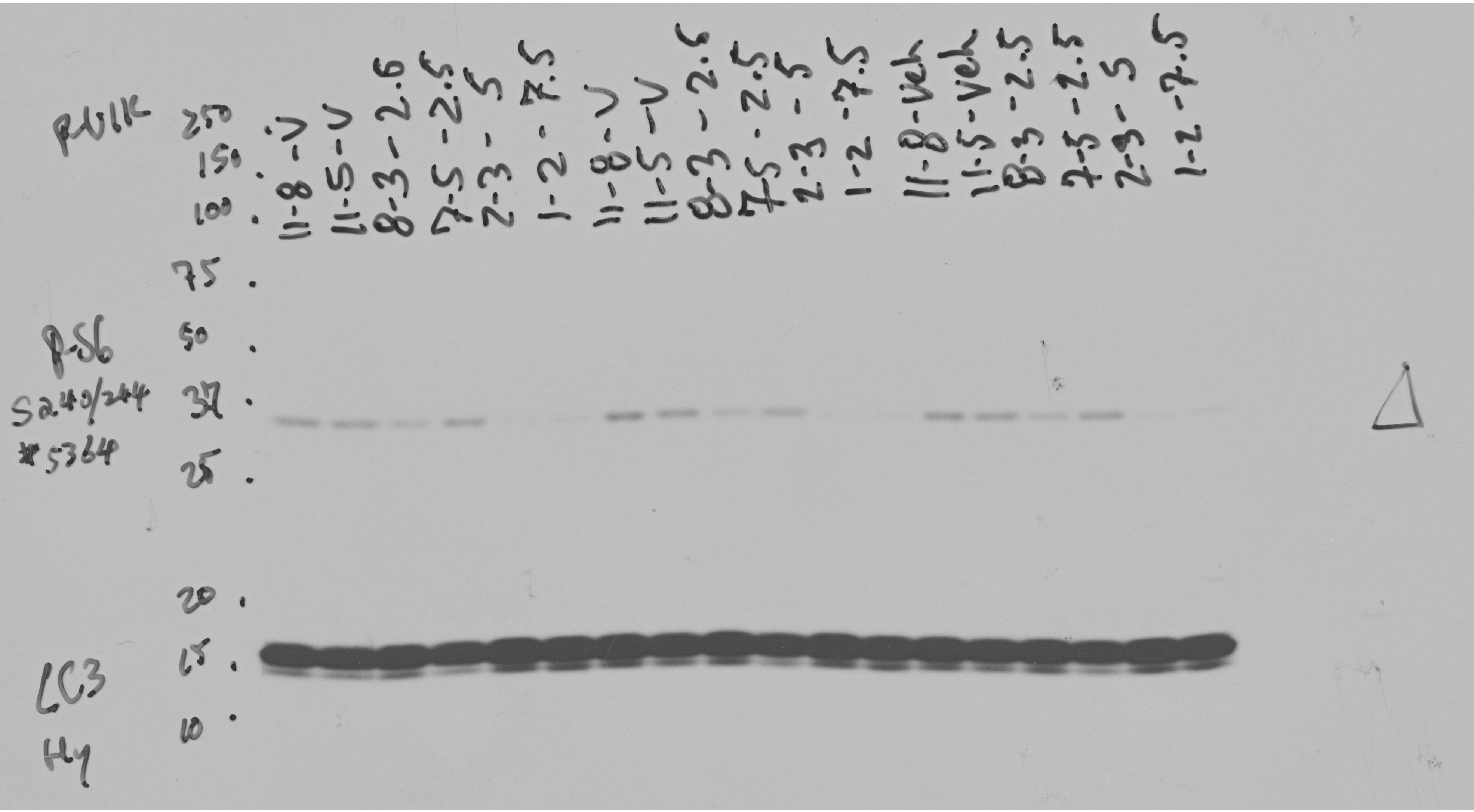

Supplement: Figure 5—figure supplement 1—source data 1. [file elife-104979-fig5-figsupp1-data1.zip › Figure 5-Figure supplement 1-Source Data 1/Blots on the right column/Anti-p-S6 (middle).tif]

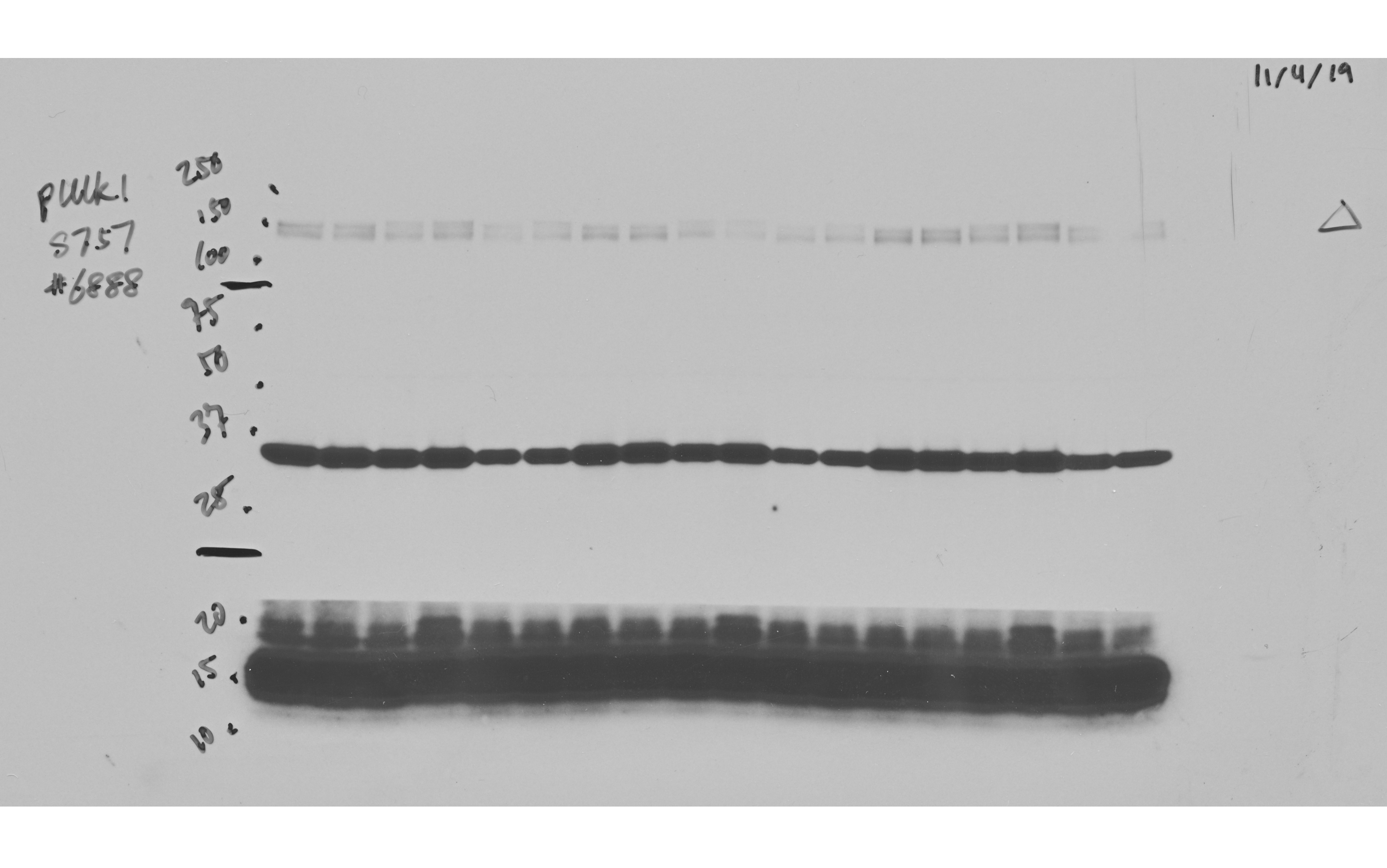

Supplement: Figure 5—figure supplement 1—source data 1. [file elife-104979-fig5-figsupp1-data1.zip › Figure 5-Figure supplement 1-Source Data 1/Blots on the right column/Anti-p-ULK1 (top).tif]

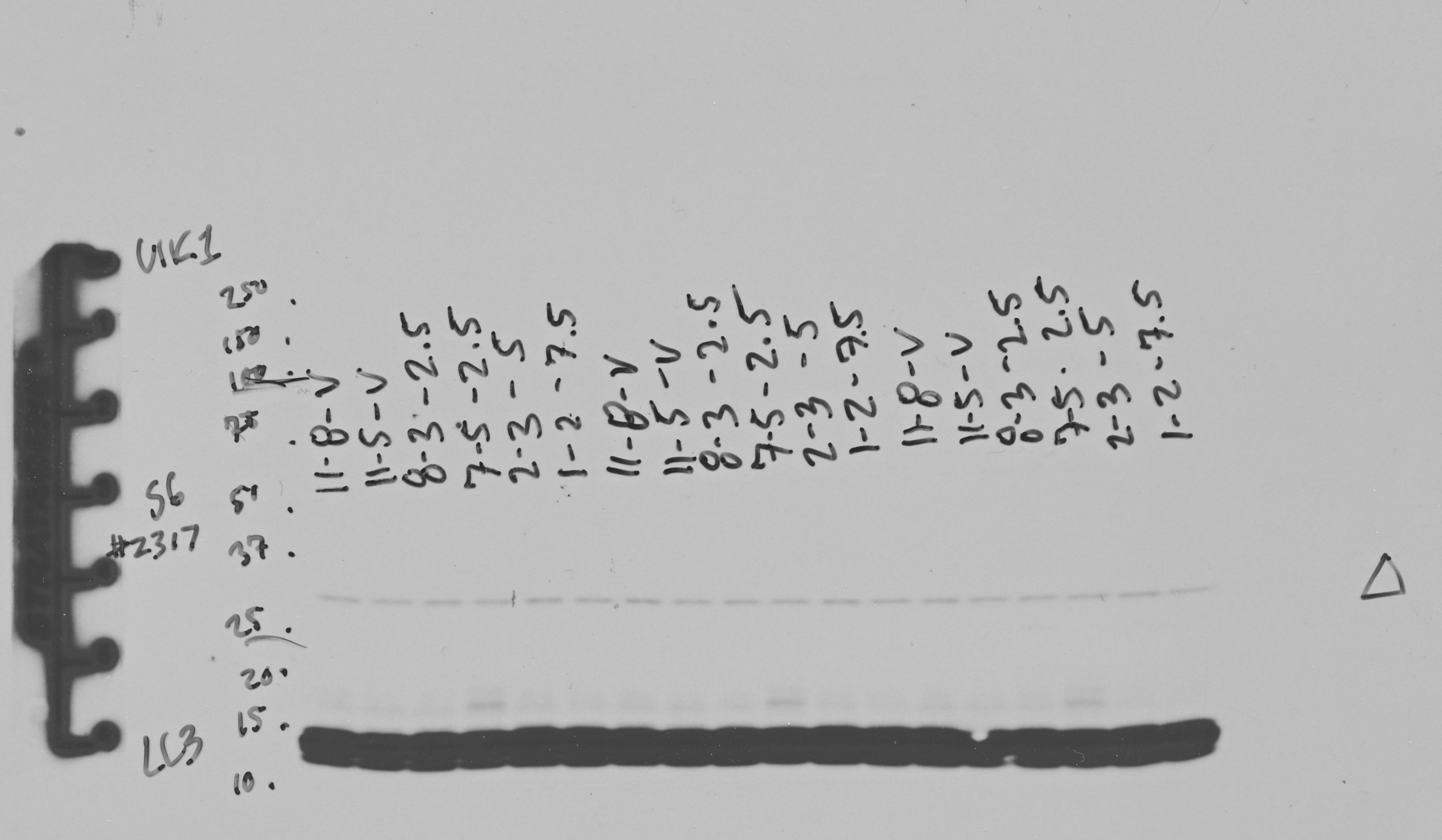

Supplement: Figure 5—figure supplement 1—source data 1. [file elife-104979-fig5-figsupp1-data1.zip › Figure 5-Figure supplement 1-Source Data 1/Blots on the right column/Anti-S6 (middle).tif]

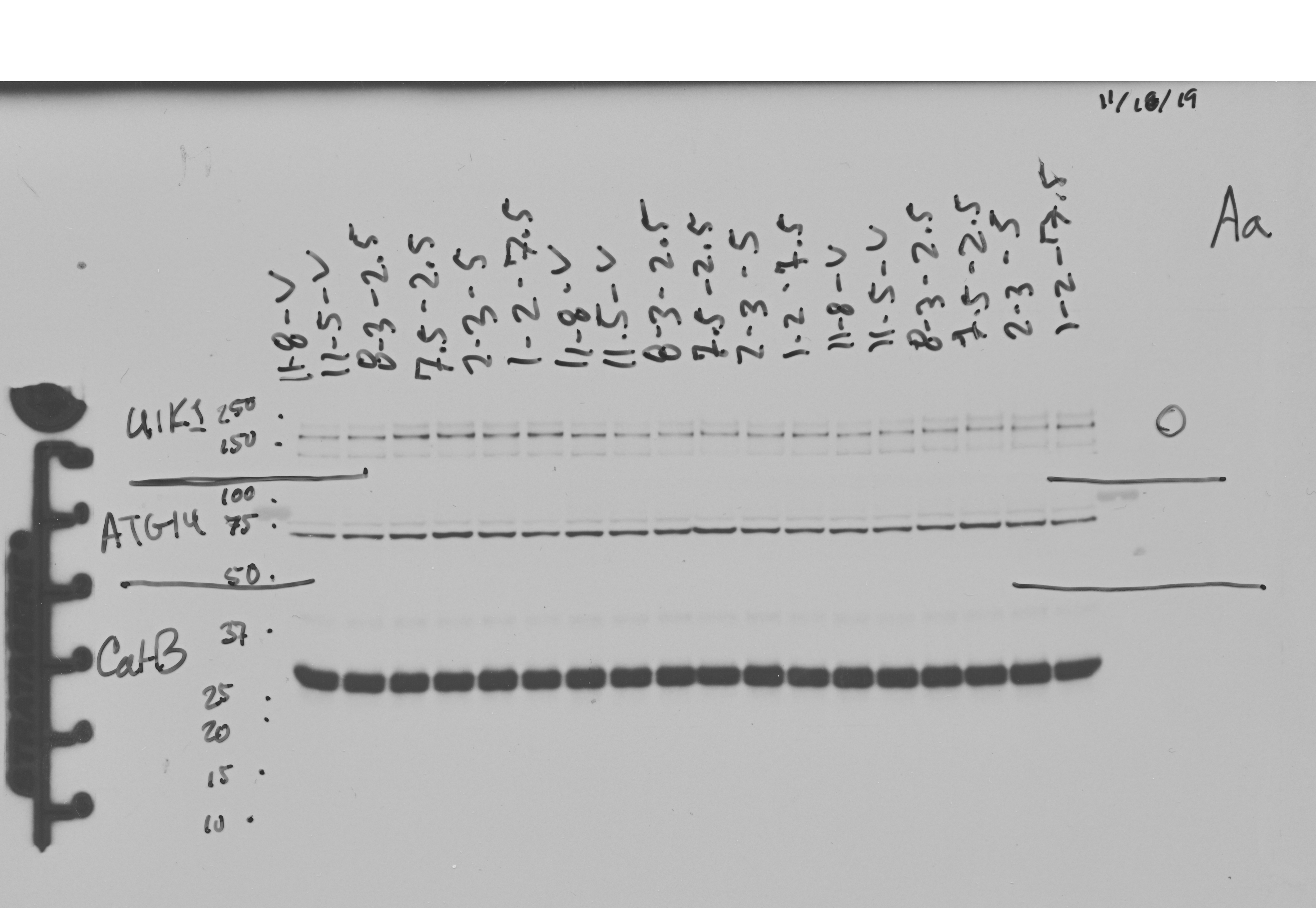

Supplement: Figure 5—figure supplement 1—source data 1. [file elife-104979-fig5-figsupp1-data1.zip › Figure 5-Figure supplement 1-Source Data 1/Blots on the right column/Anti-ULK1 (top).tif]

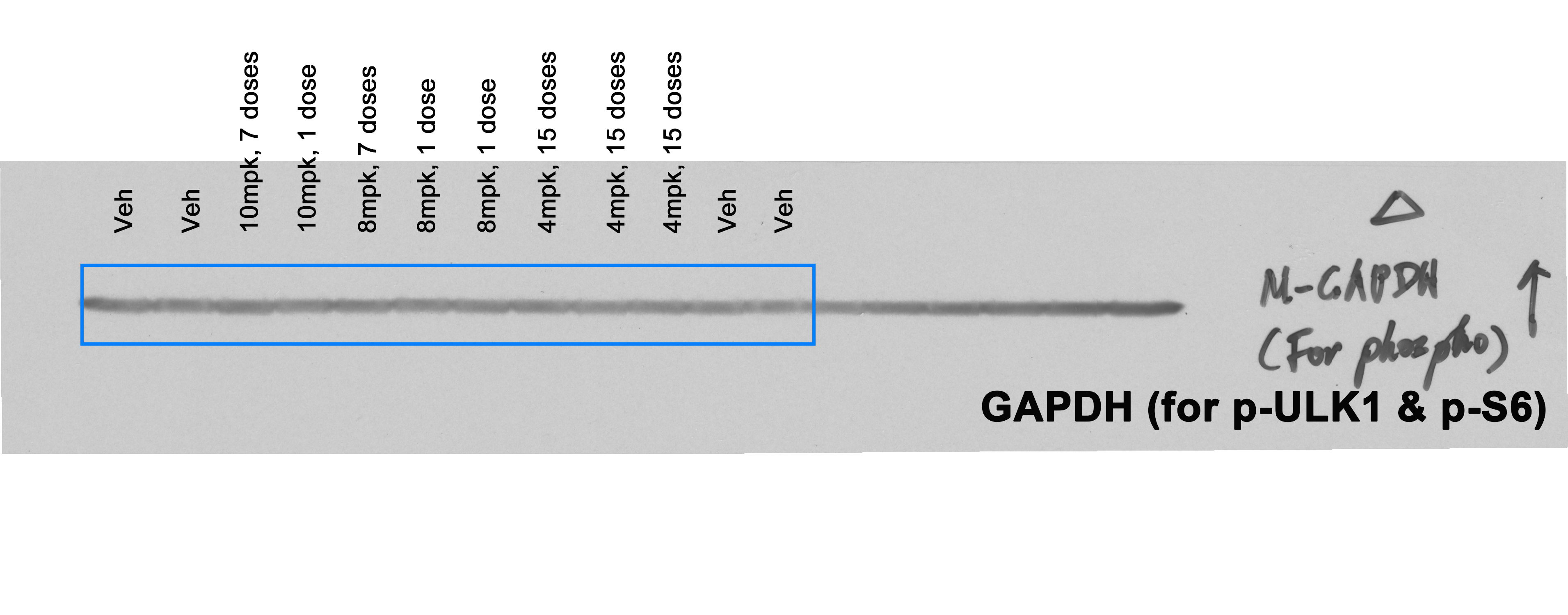

Supplement: Figure 5—figure supplement 1—source data 2. [file elife-104979-fig5-figsupp1-data2.zip › Figure 5-Figure supplement 1-Source Data 2/Blots on the left column/Anti-GAPDH (for p-ULK1 & p-S6)_Labeled.tif]

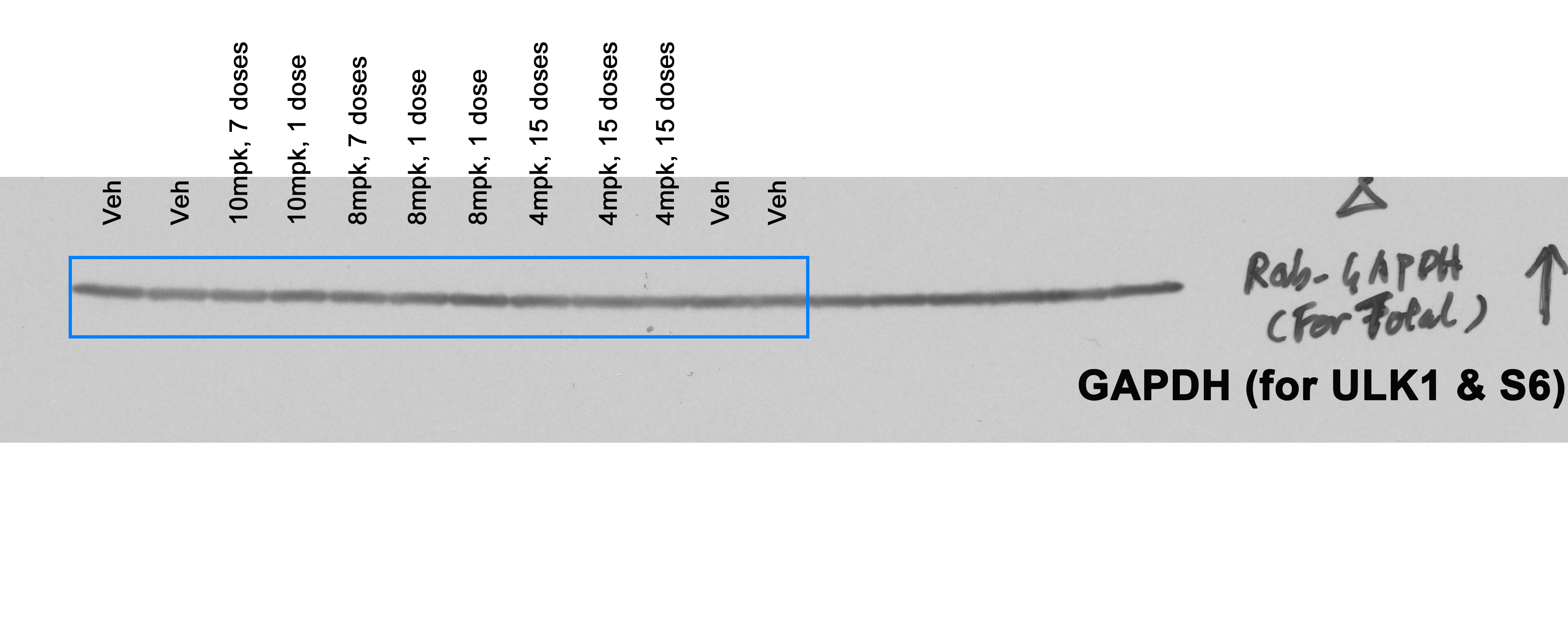

Supplement: Figure 5—figure supplement 1—source data 2. [file elife-104979-fig5-figsupp1-data2.zip › Figure 5-Figure supplement 1-Source Data 2/Blots on the left column/Anti-GAPDH (for ULK1 & S6)_Labeled.tif]

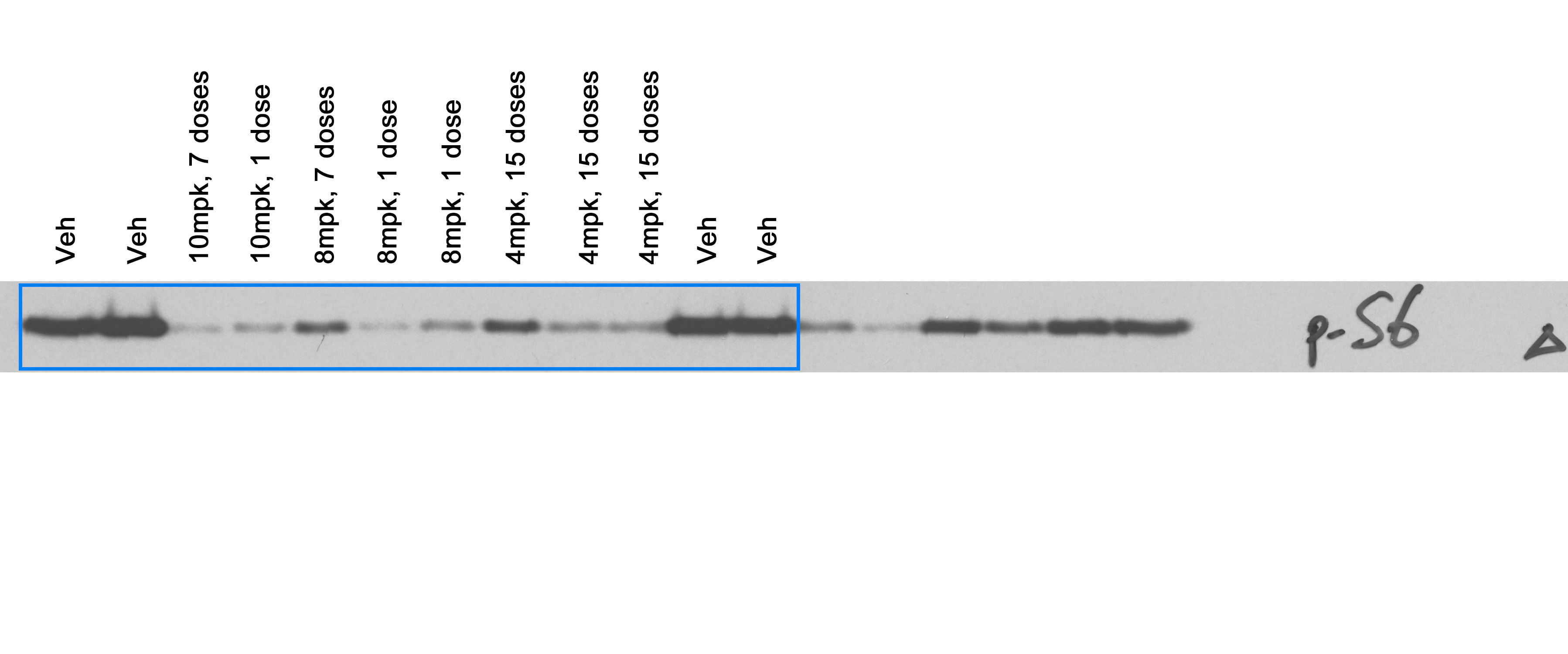

Supplement: Figure 5—figure supplement 1—source data 2. [file elife-104979-fig5-figsupp1-data2.zip › Figure 5-Figure supplement 1-Source Data 2/Blots on the left column/Anti-p-S6 (short expos)_Labeled.tif]

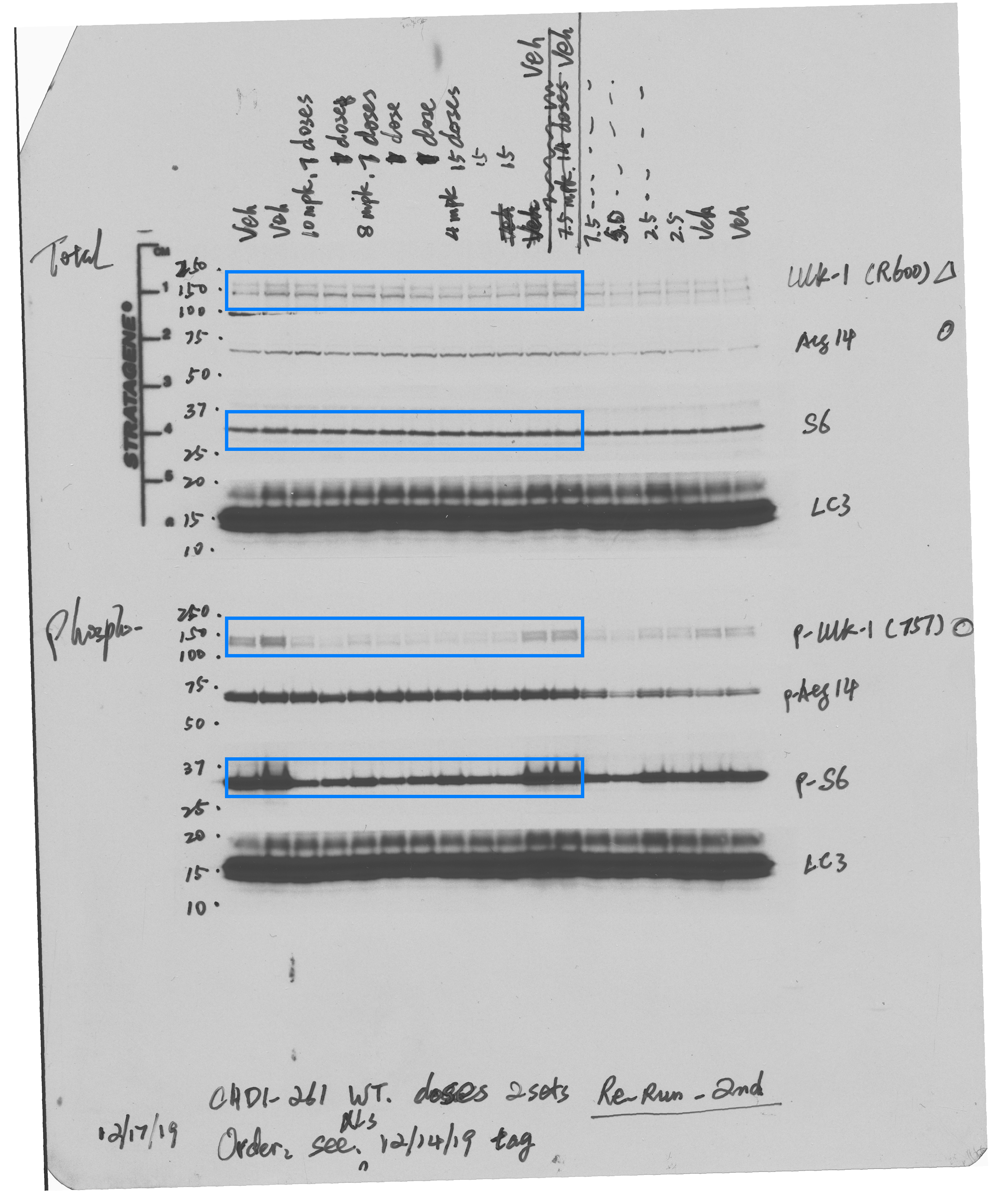

Supplement: Figure 5—figure supplement 1—source data 2. [file elife-104979-fig5-figsupp1-data2.zip › Figure 5-Figure supplement 1-Source Data 2/Blots on the left column/Anti-ULK1, S6, p-ULK1, p-S6_Labeled.tif]

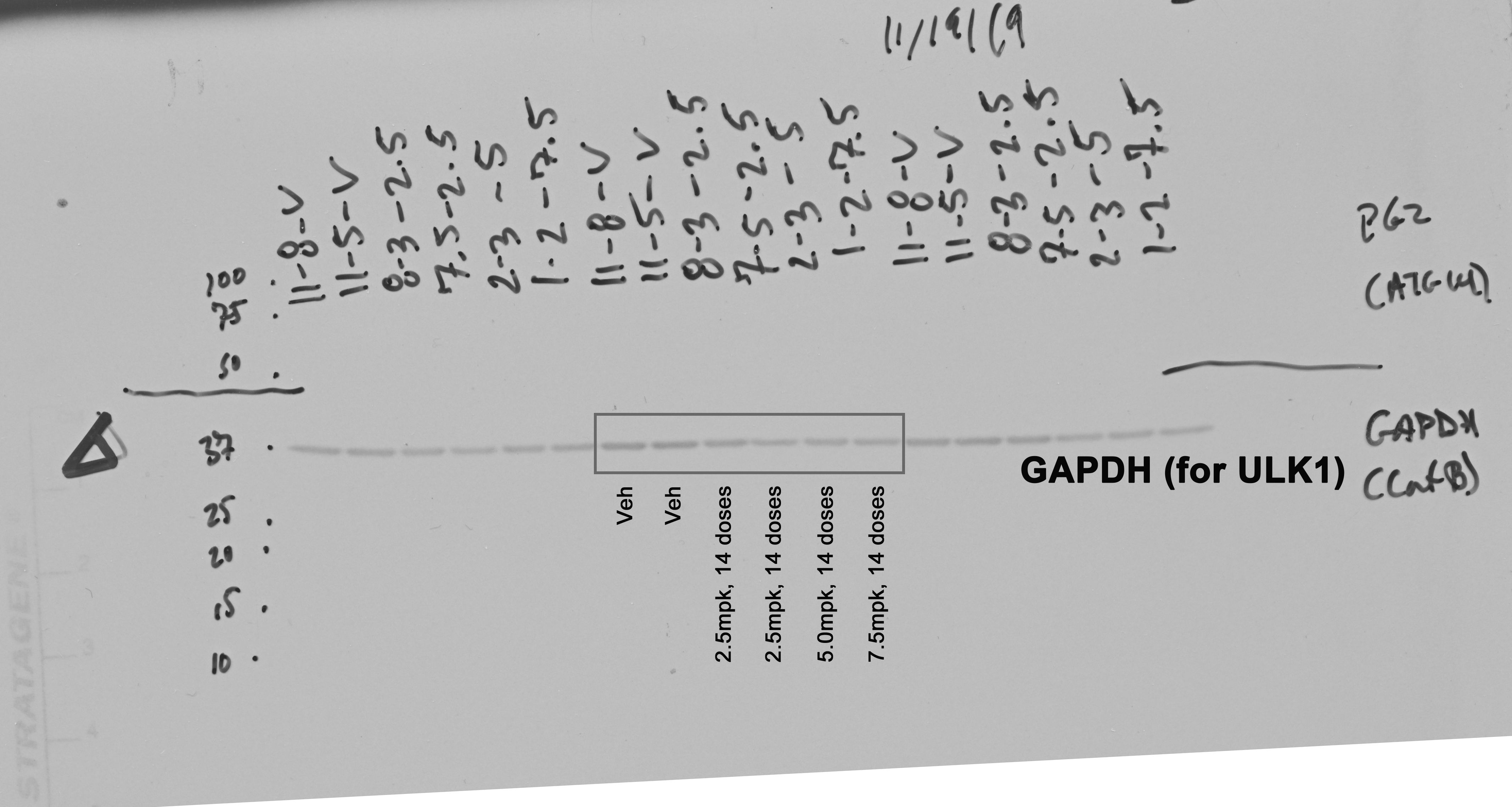

Supplement: Figure 5—figure supplement 1—source data 2. [file elife-104979-fig5-figsupp1-data2.zip › Figure 5-Figure supplement 1-Source Data 2/Blots on the right column/Anti-GAPDH (for ULK1)_Labeled.tif]

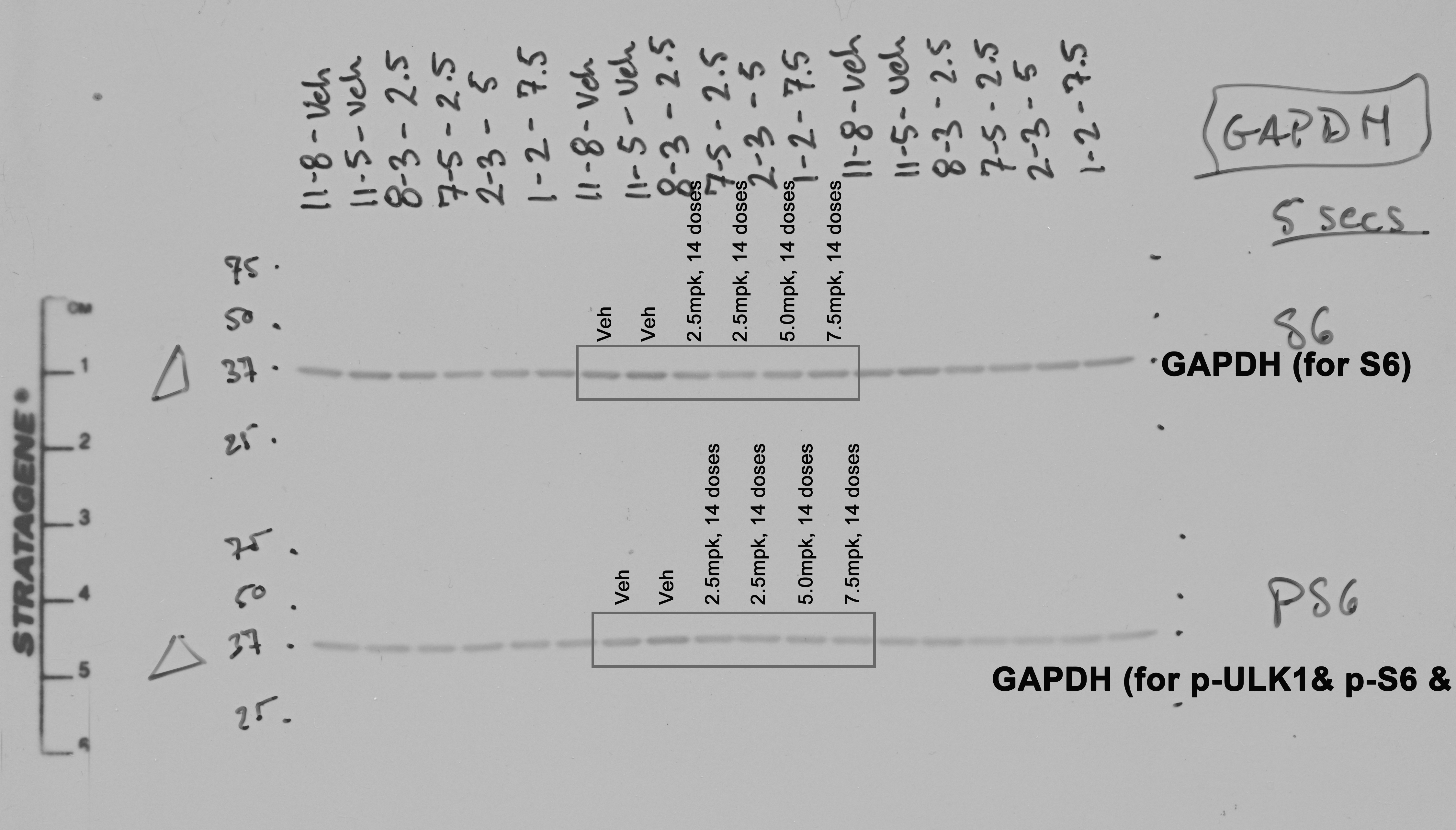

Supplement: Figure 5—figure supplement 1—source data 2. [file elife-104979-fig5-figsupp1-data2.zip › Figure 5-Figure supplement 1-Source Data 2/Blots on the right column/Anti-GAPDH (top for S6, bottom for p-ULK1 & p-S6)_Labeled.tif]

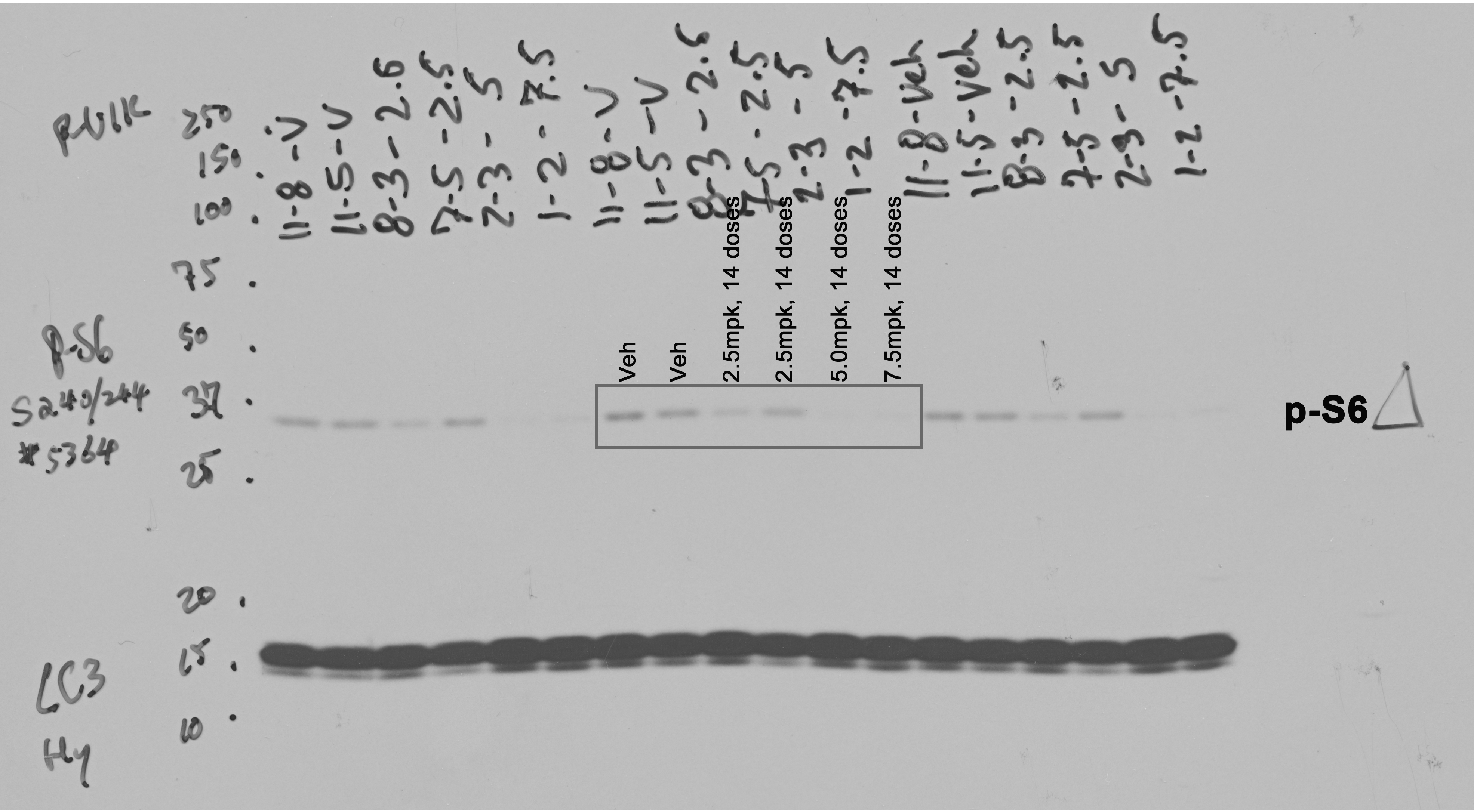

Supplement: Figure 5—figure supplement 1—source data 2. [file elife-104979-fig5-figsupp1-data2.zip › Figure 5-Figure supplement 1-Source Data 2/Blots on the right column/Anti-p-S6 (middle)_Labeled.tif]

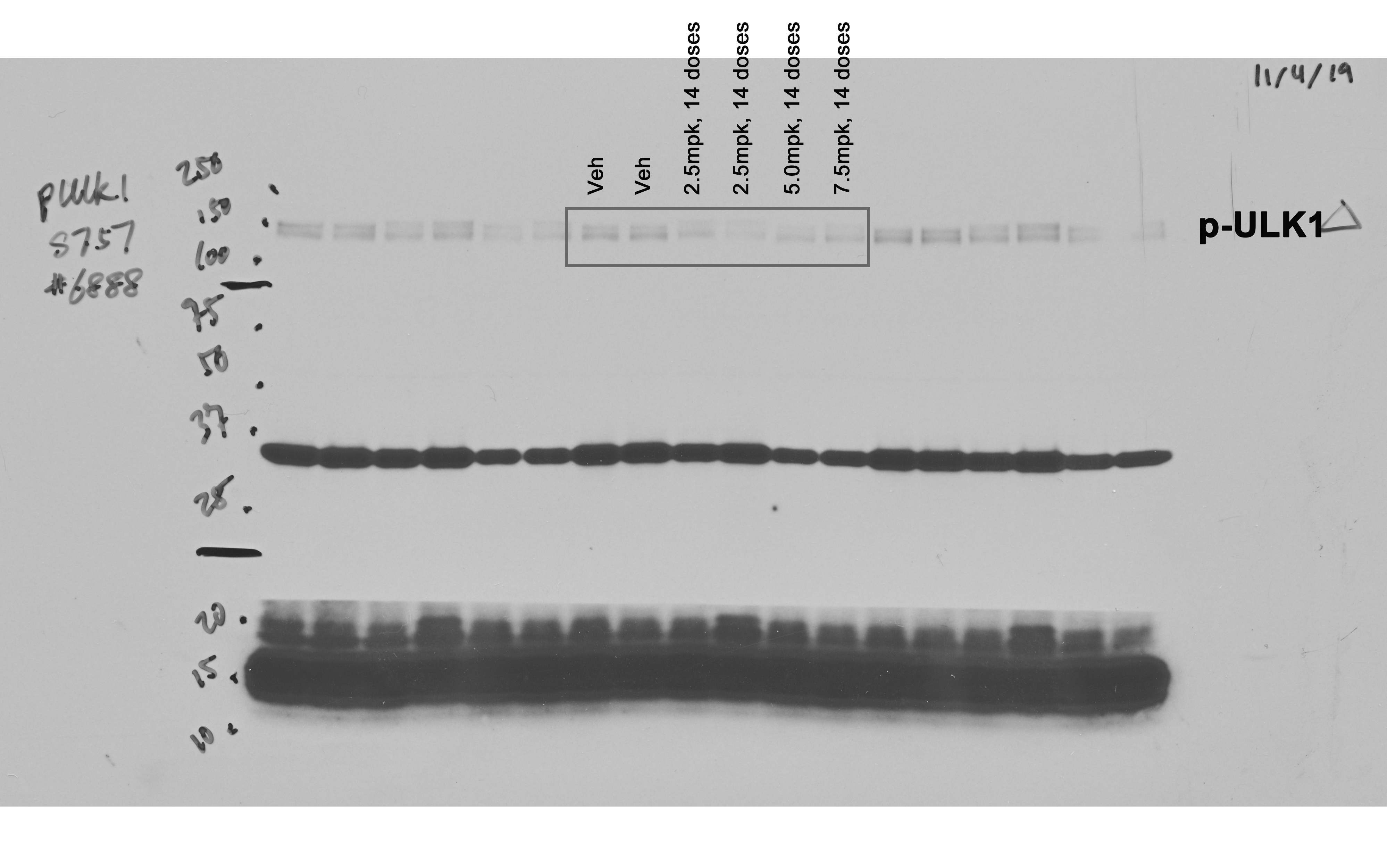

Supplement: Figure 5—figure supplement 1—source data 2. [file elife-104979-fig5-figsupp1-data2.zip › Figure 5-Figure supplement 1-Source Data 2/Blots on the right column/Anti-p-ULK1 (top)_Labeled.tif]

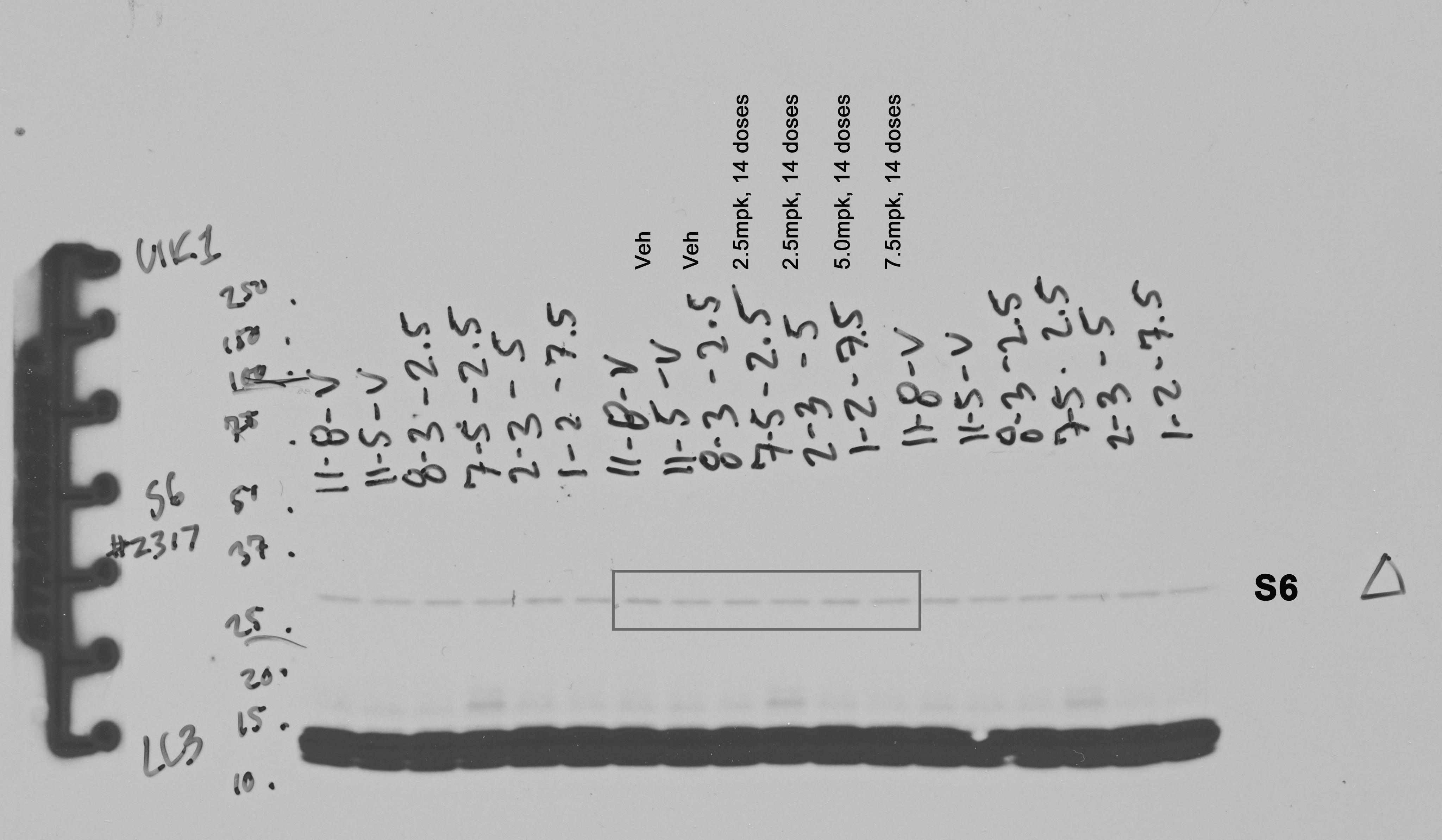

Supplement: Figure 5—figure supplement 1—source data 2. [file elife-104979-fig5-figsupp1-data2.zip › Figure 5-Figure supplement 1-Source Data 2/Blots on the right column/Anti-S6 (middle)_Labeled.tif]

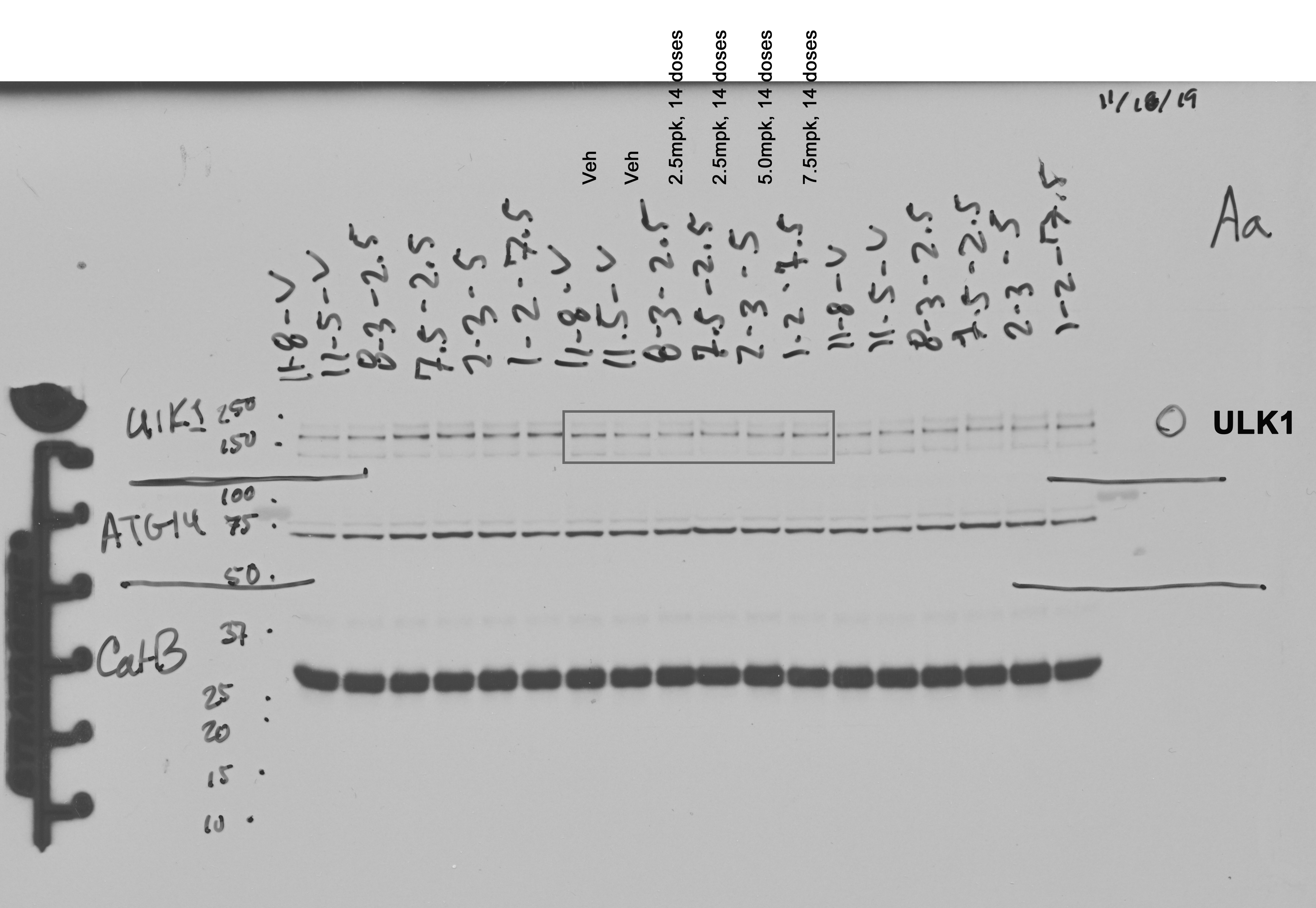

Supplement: Figure 5—figure supplement 1—source data 2. [file elife-104979-fig5-figsupp1-data2.zip › Figure 5-Figure supplement 1-Source Data 2/Blots on the right column/Anti-ULK1 (top)_Labeled.tif]
